# Upcycling of polyethylene to gasoline through a self-supplied hydrogen strategy in a layered self-pillared zeolite

**DOI:** 10.1038/s41557-024-01506-z

**Published:** 2024-04-09

**Authors:** Ziyu Cen, Xue Han, Longfei Lin, Sihai Yang, Wanying Han, Weilong Wen, Wenli Yuan, Minghua Dong, Zhiye Ma, Fang Li, Yubin Ke, Juncai Dong, Jin Zhang, Shuhu Liu, Jialiang Li, Qian Li, Ningning Wu, Junfeng Xiang, Hao Wu, Lile Cai, Yanbo Hou, Yongqiang Cheng, Luke L. Daemen, Anibal J. Ramirez-Cuesta, Pilar Ferrer, David C. Grinter, Georg Held, Yueming Liu, Buxing Han

**Affiliations:** 1 Beijing National Laboratory for Molecular Sciences, CAS Laboratory of Colloid and Interface and Thermodynamics, CAS Research/Education Center for Excellence in Molecular Sciences, Center for Carbon Neutral Chemistry, Institute of Chemistry, Chinese Academy of Sciences, Beijing, China; 2 School of Chemical Sciences, University of Chinese Academy of Sciences, Beijing, China; 3 College of Chemistry, Beijing Normal University, Beijing, China; 4 College of Chemistry and Molecular Engineering, Beijing National Laboratory for Molecular Sciences, Peking University, Beijing, China; 5 Department of Chemistry, University of Manchester, Manchester, UK; 6 Shanghai Key Laboratory of Green Chemistry and Chemical Processes, State Key Laboratory of Petroleum Molecular and Process Engineering, School of Chemistry and Molecular Engineering, East China Normal University, Shanghai, China; 7 China Spallation Neutron Source, Institute of High Energy Physics, Dongguan, China; 8 Institute of High Energy Physics, Chinese Academy of Sciences, Beijing, China; 9 Center for Physicochemical Analysis Measurements, Institute of Chemistry, Chinese Academy of Sciences, Beijing, China; 10 SINOPEC Research Institute of Petroleum Processing, Beijing, China; 11 Neutron Scattering Division, Neutron Sciences Directorate, Oak Ridge National Laboratory, Oak Ridge, TN USA; 12 Diamond Light Source, Harwell Science and Innovation Campus, Didcot, UK; 13 Institute of Eco-Chongming, Shanghai, China

**Keywords:** Heterogeneous catalysis, Solid-state chemistry, Green chemistry, Materials chemistry

## Abstract

Conversion of plastic wastes to valuable carbon resources without using noble metal catalysts or external hydrogen remains a challenging task. Here we report a layered self-pillared zeolite that enables the conversion of polyethylene to gasoline with a remarkable selectivity of 99% and yields of >80% in 4 h at 240 °C. The liquid product is primarily composed of branched alkanes (selectivity of 72%), affording a high research octane number of 88.0 that is comparable to commercial gasoline (86.6). In situ inelastic neutron scattering, small-angle neutron scattering, solid-state nuclear magnetic resonance, X-ray absorption spectroscopy and isotope-labelling experiments reveal that the activation of polyethylene is promoted by the open framework tri-coordinated Al sites of the zeolite, followed by β-scission and isomerization on Brönsted acids sites, accompanied by hydride transfer over open framework tri-coordinated Al sites through a self-supplied hydrogen pathway to yield selectivity to branched alkanes. This study shows the potential of layered zeolite materials in enabling the upcycling of plastic wastes.

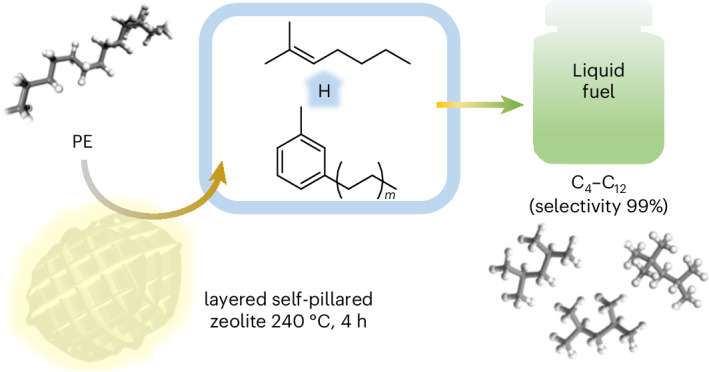

## Abstract (web summary)

The development of new methodologies to convert plastics into fuels without relying on noble metal-based catalysts is desirable. Now it is shown that a layered self-pillared zeolite enables the conversion of polyethylene to gasoline with a selectivity of 99% and yields of >80% without the need to use external hydrogen.

## Main

**Fig. 1:**
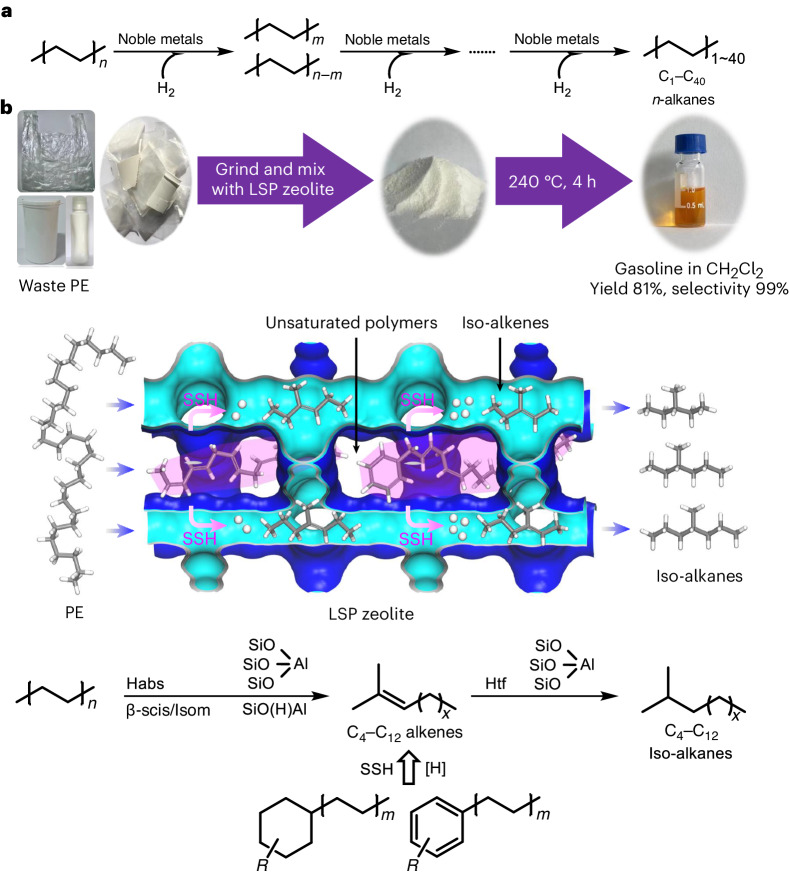
Representative routes of chemical conversion of polyolefins. **a**, Conversion of PE to *n*-alkanes through hydrogenolysis over noble metal catalysts under high-pressure H_2_. **b**, Conversion of PE to gasoline on LSP zeolites under mild conditions via an SSH strategy and the resultant gasoline shows an unprecedented selectivity of 99% for direct use as a fuel. Habs, hydride abstraction; β-scis, β-scission; Isom, isomerization; Htf, hydride transfer.

The plastic wastes are projected to exceed 25 billion tonnes by 2050^1–5^, requiring urgent developments of approaches to chemically recycling plastic wastes^6–10^. The key to convert polyolefins is to break their inert C(*sp*^3^)–C(*sp*^3^) bonds and to place effective control of selectivity to desirable products. A state-of-the-art toolbox for generating single products from polyolefins includes partial dehydrogenation and tandem isomerizing ethenolysis to yield propylene^11^, an electrified spatio-temporal heating approach to generate monomers in far-from-equilibrium state^12^ and pairing chemical oxidation and biological funnelling to form polyhydroxyalkanoates^13^. However, these strategies critically rely on the use of organometallic complexes, noble metal catalysts, a complex reactor and/or down-stream processing^14^. Emerging approaches to transform polyolefins into the fuel-range hydrocarbons offer great industrial potentials. However, fuel-range alkanes have a higher H/C ratio than polyolefins (~2.2–2.3 and 2.0, respectively). Therefore, an external H_2_ source or hydrogen enriched co-reactants is required to promote the conversion. For example, with high-pressure H_2_, liquid fuels can be generated from polyolefins through hydrogenolysis or hydrocracking on noble metal catalysts^15–21^ (Fig. 1a and Supplementary Table 1). Likewise, the addition of hexane (H/C ratio 2.33)^22^ or isopentane (H/C ratio 2.40)^23^ in depolymerization of polyolefins makes fuels by alkane metathesis or tandem cracking and alkylation. The absence of external H_2_ or co-reactants in the reaction system is highly attractive to industrial applications. Without the participation of external hydrogen source, only long-chain aromatics^24^ are formed over noble metal catalysts, and strategies using zeolites suffer from low polyethylene (PE) conversion^25^ and generate mixtures of volatile hydrocarbons^26–28^. Thus, powerful drivers exist to develop efficient, robust and economic processes for the conversion of polyolefins to transportation fuels.

In this Article, we report a self-supplied hydrogen (SSH) strategy to convert PE directly into gasoline with a selectivity of 99% and yield of 81% over a unique layered self-pillared zeolite (LSP-Z100) at 240 °C without using noble metals or any external hydrogen source (Fig. 1b). The layered structure endows LSP zeolites with extensive open framework tri-coordinated Al sites (oFTAl) as strong Lewis acid sites, resulting in superior activity to activate the inert C–H bonds of PE to supply hydrogen internally. Time-resolved analysis, solid-state nuclear magnetic resonance (NMR), an isotope-labelling technique, X-ray absorption spectroscopy and in situ inelastic neutron spectroscopy (INS) have revealed the SSH mechanism, where hydrogen is transferred from PE (or oligomers) to iso-alkenes formed by hydride abstraction, β-scission and isomerization, resulting in commercial-grade gasoline (alkenes <2 wt%) with a high research octane number of 88.0 (Fig. 1b). The inexpensive LSP-Z100 zeolites show excellent catalytic stability, consolidating its great industrial potential.

## Results and discussion

### Synthesis and characterization

**Fig. 2:**
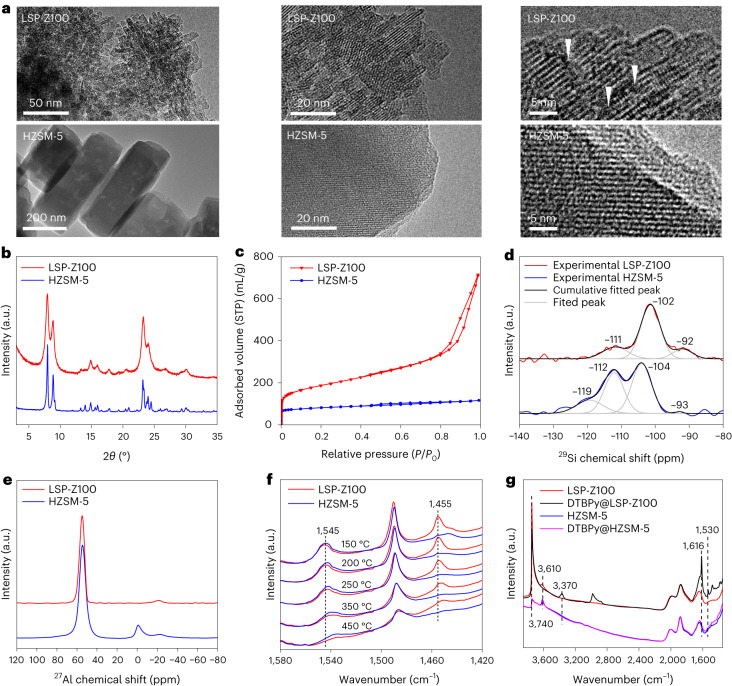
Characterization of the catalysts. **a**, High-resolution transmission electron microscopy images of LSP-Z100 and HZSM-5, showing layered self-pillared structure of LSP-Z100. Intermittent lattice fringes (white arrowheads) suggest that mesopores exist throughout LSP-Z100. Note that three sets of transmission electron microscopy images were taken at different magnifications. **b**, X-ray diffraction patterns of LSP-Z100 and HZSM-5. **c**, N_2_ adsorption/desorption isotherms of LSP-Z100 and HZSM-5. **d,** ^1^H–^29^Si cross-polarization/Magic Angle Spinning Nuclear Magnetic Resonance (MAS NMR) spectra of LSP-Z100 and HZSM-5, and de-convolution of ^29^Si-NMR spectra by Gaussian line shapes. **e**, Solid-state ^27^Al NMR spectra of LSP-Z100 and HZSM-5. **f**, IR spectra before and after adsorption of pyridine at variable temperatures on LSP-Z100 and HZSM-5. The dashed lines indicate vibrational peaks of pyridine molecules adsorbed on Brönsted acid sites (1545 cm^–^^1^) and Lewis acid sites (1455 cm^–^^1^). **g**, IR spectra before and after adsorption of DTBPy at 150 °C on LSP-Z100 and HZSM-5. The dashed lines indicate vibrational peaks of zeolite silanol group (3740 cm^–^^1^), Brönsted acid site (3160 cm^–1^) and adsorbed DTBPy molecule (3370, 1616 and 1530 cm^−1^). a.u., arbitrary units. Source data

A facile one-step hydrothermal reaction was conducted to synthesise LSP-Z100 using tetra(*n*-butyl)ammonium hydroxide as structure-directing agent (Methods, LSP-ZX refers to LSP zeolite with Si/Al ratio of *X*). It adopts MFI/MEL intergrowth structure with self-pillared layers, which feature a large external surface area and a series of mesopores (Fig. 2a–c, Supplementary Figs. 1 and 2 and Supplementary Table 2). A similar structure was found with LSP-Z75 but with reduced surface area and mesoporous volume (Supplementary Figs. 1 and 2 and Supplementary Table 2). A further decrease in the Si/Al ratio prevents the formation of LSP structures. Previous literature shows that strong acid sites and shape selectivity of zeolites are important features for conversion of PE^29^, but conventional zeolites such as HZSM-5 and HY alone showed little activity due to the narrow pores and limited acid sites on the external surface. Mesoporous materials, such as MCM-41 and SAB-15, have only weak acid sites, which are incapable of cleaving the C(*sp*^3^)–C(*sp*^3^) bonds under mild conditions. Two-dimensional structured materials are emerging catalysts showing unique advantages, such as high external surface area, but have not been used for PE upcycling. Conventional two-dimensional materials can hardly afford shape selectivity due to unrestricted surface. LSP zeolites have uniquely pillared structures and accessible acid sites for bulky molecules, distinctly different from conventional zeolites. Compared with the commercial HZSM-5, LSP-Z100 showed much higher N_2_ adsorption (Fig. 2c) and higher content of Q^3^ [Si(OSi)_3_(OH)] (−102 ppm) and Q^2^ [Si(OSi)_2_(OH)_2_] (−92 ppm) Si species (Fig. 2d and Supplementary Table 3), due to its distinct LSP network and the presence of mesopores (Fig. 2a). LSP-Z100 exhibits trace extra-framework Al sites (Fig. 2e) and a similar amount of acid sites to HZSM-5 (Supplementary Fig. 3 and Supplementary Tables 4 and 5), but shows higher amounts of strong Lewis acid sites (Fig. 2f, Supplementary Fig. 4 and Supplementary Table 5). Moreover, the acid sites of LSP-Z100 are significantly more accessible to bulky molecules as confirmed by 2,6-di-*tert*-butylpyridine (DTBPy) infra-red (IR) spectroscopy (Fig. 2g, Supplementary Fig. 5 and Supplementary Discussion 1). This is in contrast to the conventional porous materials (for example, HZSM-5, HY, MCM-41 and SBA-15), which have either limited accessibilities of acid sites or no strong acid sites.

### Catalytic performance

**Extended Data Fig. 1:**
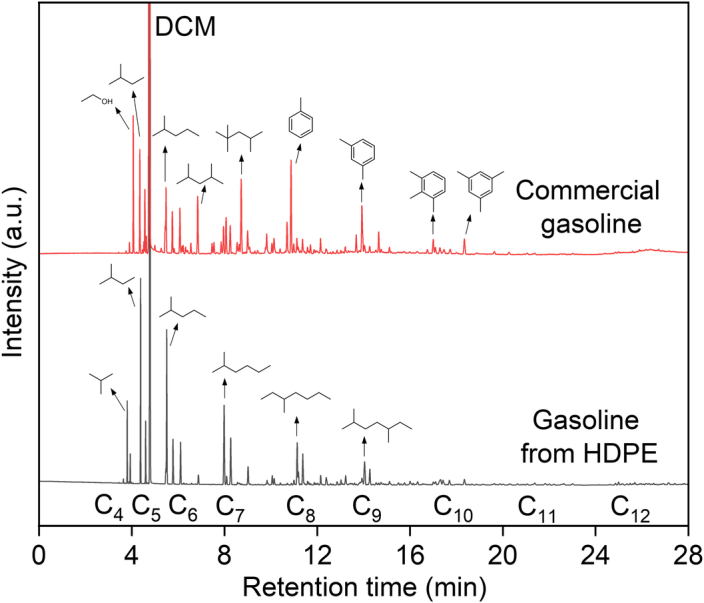
GC trace for commercial gasoline and gasoline obtained from conversion of HDPE. Reaction conditions: LSP-Z100, 0.09 g; HDPE, 0.45 g; temperature, 240 °C; reaction time, 4 h; N_2_ atmosphere, 0.1 MPa. The ethanol in commercial gasoline is an additive.

For a typical reaction, high-density PE (HDPE) was mixed with the catalyst in a mass ratio of 5:1 and the mixture was loaded into an autoclave. After purging with N_2_, the autoclave was heated to 240 °C for 4 h. The reaction was then ceased by cooling to room temperature, and the products collected for analysis. Among all the tested microporous (HZSM-5, HY and USY) and mesoporous (LSP, meso-HY, MCM-41 and SBA-15) catalysts, only LSP zeolites exhibited substantial catalytic activities (Table 1, entries 7–9, and Supplementary Table 6). Importantly, LSP-Z100 gave a high conversion of HDPE of 81.8% (Table 1, entry 8) and an unprecedented selectivity of >99% to C_4_–C_12_ compounds (gasoline range) with negligible C_1_–C_3_ compounds (<1%) (Supplementary Tables 7 and 8). This is attributed to the advantage that the formation of C_4+_ compounds, particularly branched C_4+_ compounds through A-type (tertiary–tertiary) or B-type (secondary–tertiary) β-scission, is energetically more favourable^30,31^ and can rapidly take place at mild reaction temperatures (Supplementary Discussion 2). With a prolonged reaction time of 24.5 h, the yield of gasoline increased to 87.2% (Table 1, entry 9). Compared with commercial gasoline, the liquid product from this reaction is of premium quality in terms of higher content of branched alkanes (44.1% versus 72.5%), comparable research octane number (86.6 versus 88.0, Supplementary Table 9) and notably lower contents of contaminating aromatics (20.3% versus 10.1%) and olefins (7.4% versus 1.4%) (Table 1, entries 8 and 10, and Extended Data Fig. 1). The reduced olefin content is probably related to the shortened diffusion path in LSP-Z100. This suggests that intermediate alkenes diffuse more rapidly within the catalyst, facilitating adequate interaction with active sites in a confined system. Consequently, this promotes the transformation of intermediate alkenes into alkanes (that is, the main component of gasoline). For instance, reducing the particle size of HZSM-5 from 4 μm to 200 nm resulted in a decrease in selectivity for alkenes (Supplementary Table 10, entries 1 and 2). A series of reported best-behaving catalysts (for example, fresh fluid catalytic cracking (FCC) catalysts^27^, spent FCC catalysts^27^, short *b*-axis ZSM-5 (ref. ^28^), [C_4_Py]Cl–AlCl_3_(ref. ^23^), Ru/C (ref. ^16^), Pt/γ–Al_2_O_3_ (ref. ^24^) and Pt/WO_3_/ZrO_2_ + HY(30) (ref. ^21^)) have also been tested in our system (Table 1, entries 11–17), but they show regrettable activity due to the lack of external hydrogen source or additives.

**Table 1:** Summary of the HDPE conversion and product yields over various catalysts^a^

Entry	Catalysts	Conversion (%)	Yield (%)			Components of gasoline range products (%)				
			C_1_–C_3_	C_4_–C_12_ (gasoline range)	>C_12_^b^	*n*-Alkanes	*i*-Alkanes	Alkene	Cycloalkanes	Aromatics
1	HZSM-5	35.1	0.6	34.5	0.0	16.0	43.0	27.8	5.7	7.5
2	HY	3.6	0.1	3.5	0.0	3.3	78.3	3.9	4.1	10.4
3	USY	8.6	0.1	8.5	0.0	3.8	77.2	1.0	6.1	11.9
4	Meso-HY	19.7	0.4	19.3	0.0	5.2	79.5	4.7	5.3	5.3
5	MCM-41	9.0	0.3	8.7	0.0	8.1	78.5	5.2	3.1	5.1
6	SBA-15	<1	–	–	–	–	–	–	–	–
7	LSP-Z75	78.3	1.8	76.5	0.0	10.1	71.7	3.2	4.5	10.5
8	LSP-Z100	81.8	0.6	81.2	0.0	11.7	72.5	1.4	4.3	10.1
9	LSP-Z100^c^	90.2	3.0	87.2	0.0	14.6	68.7	0.8	4.2	11.7
10	Commercial gasoline^d^	–	–	–	–	9.8	44.1	7.4	6.2	20.3
11	Fresh FCC catalyst	5.8	0.2	5.6	0.0	8.5	86.7	4.4	0.4	0.0
12	Spent FCC catalyst	<1	–	–	–	–	–	–	–	–
13	Short *b*-axis ZSM-5	12.0	0.2	11.8	0.0	10.1	21.4	53.4	6.7	8.5
14	[C_4_Py]Cl–AlCl_3_	<1	–	–	–	–	–	–	–	–
15	Ru/C	<1	–	–	–	–	–	–	–	–
16	Pt/γ–Al_2_O_3_	<1	–	–	–	–	–	–	–	–
17	Pt/WO_3_/ZrO_2_ + HY(30)	12.5	0.3	12.2	0.0	7.9	81.2	1.2	0.4	9.3

^a^Reaction conditions, catalyst, 0.09 g; HDPE, 0.45 g; temperature, 240 °C; reaction time, 4 h; N_2_ atmosphere, 0.1 MPa. ^b^C_12+_ compounds in liquid products were not detected by GC. ^c^Reaction time is 24.5 h. ^d^Commercial gasoline with 12.2% ethanol as an additive.

### SSH pathway

**Fig. 3:**
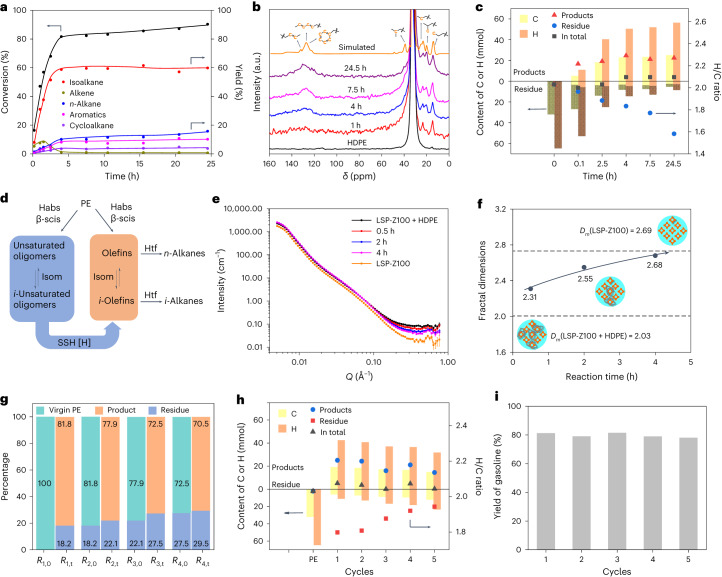
Time course of conversion of HDPE over LSP-Z100 and stability of LSP zeolites. **a**, The trends of conversion of PE and yield of products. Reaction conditions: catalyst, 0.09 g; HDPE powder, 0.45 g; reaction temperature, 240 °C; N_2_, 0.1 MPa. **b**, Solid-state ^13^C NMR spectra of the reaction residue, showing the presence of unsaturated species and methylene group in residue (32 ppm, Supplementary Fig. 6 and Supplementary Table 12). a.u., arbitrary units. **c**, Variation of C and H contents and H/C ratio of short chain products and solid residues as a function of reaction time. Bars with dots are attributed to unreacted PE. **d**, Scheme of PE depolymerization pathway and SSH pathway. Habs, hydride abstraction; β-scis, β-scission; Isom, isomerization; Htf, hydride transfer. **e**, SANS spectra for HDPE and reacted HDPE at 190 °C. All SANS spectra shown are after the subtraction of SANS spectrum of the empty cell. **f**, Mass fractal dimensions of LSP-Z100, mixture of LSP-Z100 and HDPE, and reaction of mixture at different reaction time obtained from SANS spectra. Simplified models of LSP-Z100 and HDPE are shown based on SANS results (LSP-Z100, orange; HDPE, grey; argon, cyan). **g**, A comparison of reactions 1–4 over LSP zeolites at 240 °C for 4 h. After each reaction, the catalyst was used directly after drying. Fresh PE was added to maintain the same mass of hydrocarbons in *R*_*x*,0_ for each reaction. *R*_*x*,0_, before reaction; *R*_*x*,*t*_, after reaction, where *x* = 1,2,3,4. Atomic utilization = (sum of products/total added virgin PE) × 100%. **h**, The variation of C and H content and H/C ratio of products and solid residue in reactions 1–4 over LSP zeolites at 240 °C for 4 h. **i**, Comparison of gasoline yield over five cycles of reactions over LSP zeolite at 240 °C for 4 h. After each cycle, the catalyst is calcined at 550 °C under air. Source data

**Extended Data Fig. 2:**
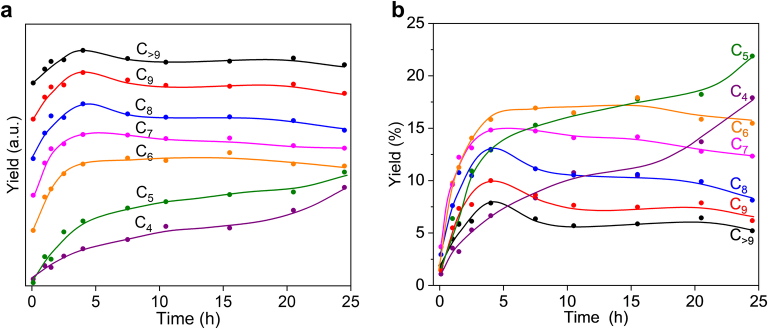
Trends of product yield in carbon numbers in the time course study. The profiles in (a) have been moved along Y axis, which only show trend rather than absolute value. The profiles in (b) are plotted from original data and show the actual yields of species with certain carbon numbers.

The time course of HDPE depolymerization over LSP-Z100 at 240 °C was studied by analysing the products using gas chromatography (GC), GC mass spectroscopy (MS) and by analysing the solid residues using elemental analysis, ^13^C MAS NMR, ^1^H NMR and glow discharge electrospray ionization (GD ESI) mass spectrometry. At 0.2 h (Fig. 3a), alkanes and alkenes were observed, indicating that LSP-Z100 is extremely active to crack PE. Between 0.2–4 h, the yield of alkenes experienced a slight increase and then decreased to <2%, whereas rapid formation of alkanes continued until the completion of reaction when the yield of alkanes reached 75%. From 4 h to 24.5 h, secondary cracking of the C_6_–C_9_ products took place (Extended Data Fig. 2), as well as cyclization and aromatization to produce only a small fraction of aromatics and cycloalkanes (Fig. 3a). The ^13^C NMR spectrum of the solid residue from the reaction showed a clear signal of methyl group (18 ppm), branched (21 ppm) and unsaturated species (120–150 ppm) (Fig. 3b, Supplementary Fig. 6, Supplementary Tables 11 and 12 and Supplementary Discussion 3). This indicates that part of PE undergoes partial cracking, isomerization and hydride transfer to form unsaturated oligomers (H/C <2) as internal hydrogen source. The SSH pathway is validated by elemental analysis of products and reaction residues. As shown in Fig. 3c, C and H content transfer from the solid residue to the product with the increase of reaction time. The H/C ratio of products raises from initially 2.03 to ~2.2 as the reaction starts, while the H/C ratio of residue decreases during the reaction, confirming the internal hydrogen transfer (Fig. 3d). The H/C ratio of total hydrocarbons (products and solid residues) maintained at ~2.03 throughout the reaction (Fig. 3c and Supplementary Table 13) and total mass balances for all hydrocarbons typically closed to within 1.8% (Supplementary Table 14). The SSH strategy inevitably generates a small amount (18.2%) of solid residues (H/C <2). The solid residues were treated with hydrofluoric acid (HF) to remove the zeolite, and the remaining hydrocarbons were separated through dichloromethane (DCM) extraction (Supplementary Table 15) for further characterization by ^1^H NMR, ^13^C NMR and GD ESI mass spectrometry. The fraction insoluble in DCM comprises unreacted PE (Supplementary Fig. 7). The DCM-soluble portion is predominantly composed of long-chain alkylaromatics and long-chain alkylcyclic compounds (unsaturated oligomers), with a molecular weight range between 100 and 1,000 (Supplementary Figs. 8–11 and Supplementary Tables 16 and 17). The observation of these unsaturated oligomers further corroborates the proposed SSH mode. Thermogravimetric analysis (TGA) shows that the yield of coke is only 0.58% (Supplementary Figs. 12 and 13 and Supplementary Discussion 4) and thus the residual comprises primarily unsaturated oligomers and uncracked PE, probably restricted by hindered diffusion.

In situ small-angle neutron scattering (SANS) was applied to investigate the diffusion of PE and intermediates to the catalysts during the reaction (Fig. 3e). The decrease on intensity of neutron scattering in the high *Q* region (>0.2 Å^−^^1^) is due to lower incoherent scattering caused by hydrogen atom, suggesting the cracking of HDPE and evaporation of generated light hydrocarbons (Fig. 3e). In addition, the change of mass fractal dimension in the *Q* region of 0.02–0.2 Å^−^^1^ reflects that the components of reaction mixture had altered during reaction (Fig. 3f and Supplementary Fig. 14). Before 4 h, the mass fractal dimension increased gradually, indicating the surface-assisted cracking of PE chains around active sites. In this period, the viscosity of the system decreases because the cracking of long-chain PE into shorter chain oligomers^32^. At 4 h, the mass fractal reached 2.68 (close to 2.69, the mass fractal dimension of LSP-Z100), indicating the approximate depletion of coil chain of PE upon reaction. This reveals the hindered diffusion of residual oligomers to the active sites, consistent with the catalysis and TGA results.

To promote the diffusion and atomic economy, fresh PE (81.8%) is mixed with the reaction residue (18.2%) after 4 h (*R*_1_) and undergoes a new cycle of reaction (*R*_2_) (Fig. 3g). The newly added PE acts as solvent to facilitate mass transport of residual oligomers as well as reaction substrate in the new reaction. Importantly, the result of the new cycle is comparable with the first reaction (Fig. 3g and Supplementary Table 18), suggesting that the previous reaction residues can be converted to products with fresh PE, although another solid residue (22.1%) was formed from the new cycle. This reveals the key role of promoted diffusion of residues, thus preserving the activity of LSP-Z100. After four cycles of reaction, the yield of gasoline is maintained at >70% and the total atomic utilization is as high as 91% (Fig. 3g). The H/C contents are balanced during the four reactions (Fig. 3h and Supplementary Table 19) and little coke was formed (~0.5%, Supplementary Table 20 and Supplementary Fig. 15) owing to the widely opened pores of LSP zeolites to prevent coke formation. The excellent stability of LSP-Z100 was further demonstrated by cycling testing (the residues were removed by calcination before next cycle), in which the yield of gasoline remained at 80% over five cycles (Fig. 3i). The characterization of used catalysts confirms little change to the structure and acidic sites, demonstrating the excellent structural stability of LSP-Z100 (Supplementary Figs. 16–18). Thus, LSP-Z100 enables the success of SSH mode for conversion of PE to gasoline with coke resistance and high atomic utilization.

### Identification of active sites

**Fig. 4:**
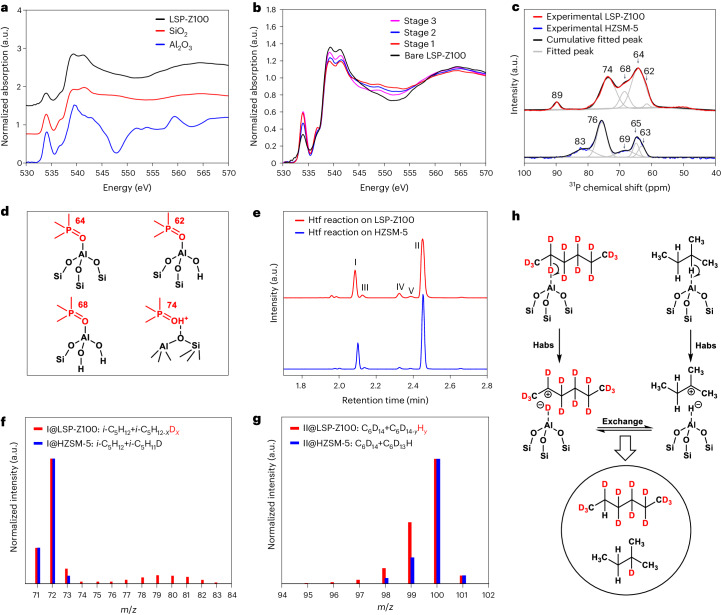
Identification of active sites of LSP-Z100 by NEXAFS, NMR and isotope-labelling technique. **a**, O *K*-edge NEXAFS for LSP-Z100, SiO_2_ and Al_2_O_3_. The absorption edges of LSP-Z100 were consistent with those of SiO_2_ and Al_2_O_3_. **b**, O *K*-edge NEXAFS spectra for LSP-Z100 before reaction and LSP-Z100 during the reaction. **c**, ^31^P MAS NMR spectra of LSP-Z100 and HZSM-5 loaded with TMPO. **d**, View of structure of Al sites with adsorption of TMPO. **e**, GC traces of *i*-C_5_H_12_/C_6_D_14_ hydride transfer reactions by LSP-Z100 and HZSM-5. I–V are mainly composed of *i*-C_5_H_11_D, C_6_D_13_H, *i*-C_5_D_11_H, *i*-C_6_D_13_H and C_6_D_12_H_2_, respectively (Extended Data Figs. 3 and 4). **f**, Mass spectra of 2-methylbutane after reaction on HZSM-5 and LSP-Z100. **g**, Mass spectra of hexane after reaction on HZSM-5 and LSP-Z100. **h**, Scheme of *i*-C_5_H_12_/C_6_D_14_ hydride transfer reactions. Habs, hydride abstraction; a.u., arbitrary units. Source data

The local environment of the acid sites Si–O–Al was interrogated through near-edge X-ray absorption fine structure (NEXAFS) analysis at the oxygen *K* edge. The absorption edges of LSP-Z100 were consistent with those of SiO_2_ and Al_2_O_3_ (Fig. 4a). The peak at 534 eV is due to adsorbed water in the materials^33^. The region between 538 eV and 543 eV (white line) in the O *K*-edge NEXAFS spectrum is attributed to transitions from O 1*s* to O 2*p* anti-bonding states hybridized with Si 3*s*, Si 3*p*, Al 3*s* or Al 3*p* character^34–36^. To understand the interaction between acid sites and guest molecules, the conversion of HDPE over LSP-Z100 was studied at different reaction stages. At stage 1 of reaction (190 °C for 2 h), LSP-Z100 showed decrease in the white line intensity (538–543 eV) relative to bare LSP-Z100 (Fig. 4b and Supplementary Fig. 19), suggesting a partial filling of the O 2*p* anti-bonding orbital. This confirms that the generated unsaturated species (for example, alkenes) during the reaction were bound to the Si–O–Al sites by donating electrons to the O 2*p* anti-bonding orbital. In addition, the increase in intensity in the region of 545–555 eV is due to scattering from the adsorbed unsaturated species on Si–O–Al sites. With the reaction ongoing (stage 2 of reaction, 230 °C for 1 h, Fig. 4b), the white line intensity increased, indicating the adsorbed alkenes are saturated because of hydride transfer reaction. Importantly, at the stage 3 of reaction (240 °C for 2 h), the intensity was closed to bare LSP-Z100, indicating the complete regeneration of Si–O–Al sites upon conversion of alkenes to alkanes that could desorb readily from the catalysts.

**Extended Data Fig. 3:**
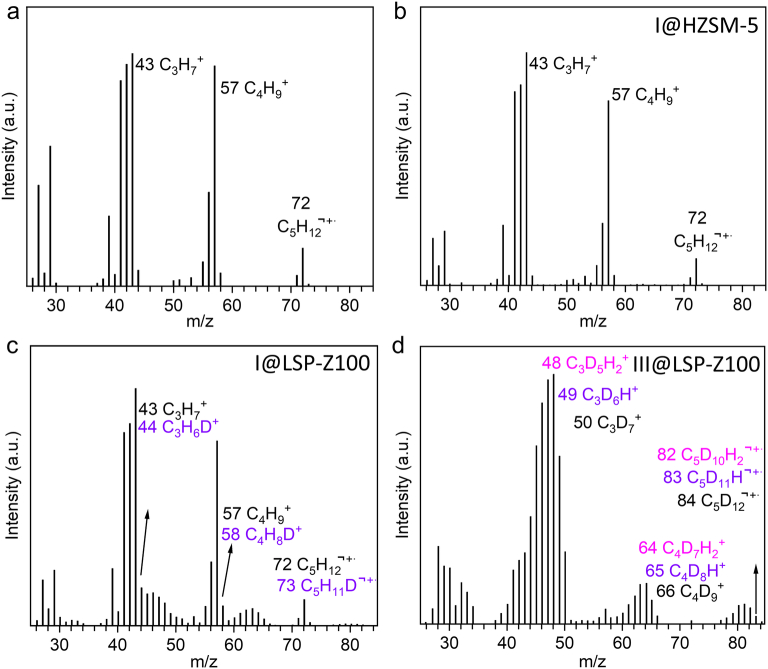
Standard mass spectrum of 2-methylbutane (a) and mass spectra of 2-methylbutane after the reaction on HZSM-5 (b) and LSP-Z100 (c, d). The Mass spectra of GC peaks I@LSP-Z100 and III@LSP-Z100 in GC traces show that I@LSP-Z100 is composed of i-C_5_H_12_ and i-C_5_H_11_D, and III@LSP-Z100 is composed of i-C_5_D_12_, i-C_5_D_11_H and i-C_5_D_10_H_2_. The generation of III@LSP-Z100 is because of dehydrogenation, oligomerisation of C_6_D_14_ and cleavage of the formed oligomers. Reaction conditions: catalyst, 0.075 g; 2-methylbutane, 0.075 g; n-hexane (d-14), 0.15 g; temperature, 240 °C; reaction time, 1 hour; N_2_ atmosphere, 0.1 MPa. Source data

**Extended Data Fig. 4:**
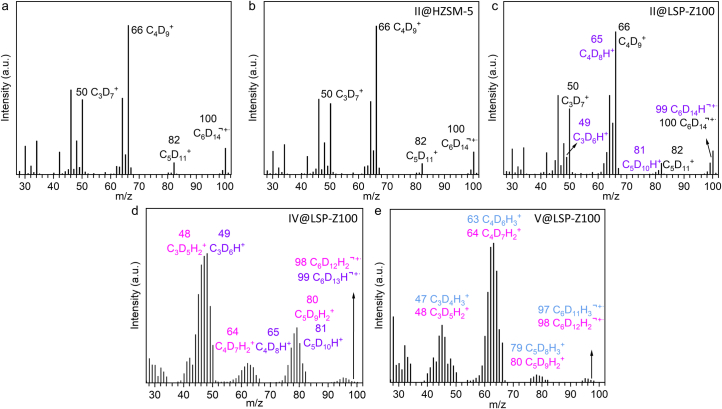
Standard mass spectrum of C_6_D_14_ (a) and mass spectra of deuterated hexane after reaction on HZSM-5 (b) and LSP-Z100 (c-e). The Mass spectra show that II@LSP-Z100 is composed of C_6_D_13_H and C_6_D_14_, and IV@LSP-Z100 is composed of i-C_6_D_13_H and i-C_6_D_12_H_2_, and V@LSP-Z100 is composed of C_6_D_12_H_2_ and C_6_D_11_H_3_. The base peak of IV@LSP-Z100 mass spectra at m/z = 48 demonstrates the deuterated hexane has isomerized into deuterated 2-methylbutane. Reaction conditions: catalyst, 0.075 g; 2-methylbutane, 0.075 g; n-hexane (d-14), 0.15 g; temperature, 240 °C; reaction time, 1 hour; N_2_ atmosphere, 0.1 MPa. Source data

The structures of active sites were further investigated by ^31^P NMR spectroscopy of trimethylphosphine oxide (TMPO) as a probe molecule^37^. LSP-Z100 possesses a large amount of FTAl sites, which act as Lewis acid sites to coordinate with lone pair electrons of basic probe molecule (64 ppm, TMPO ∙ ∙∙Al(OSi)_3_; 62 ppm, TMPO ∙ ∙∙Al(OSi)_2_(OH); 68 ppm, TMPO ∙ ∙∙Al(OSi)(OH)_2_) (Fig. 4c,d and Supplementary Table 21). In contrast, the spectrum of TMPO adsorbed on HZSM-5 shows higher intensity of TMPOH^+^ ions protonated by Brönsted acid sites (76 ppm) and less prominent signal of Lewis acid coordinated molecules (63–69 ppm). This result is consistent with the Py-IR experiment that shows higher L/B ratio of LSP-Z100 than HZSM-5 (Fig. 2f and Supplementary Table 5). In addition, the peaks related to FTAl sites of LSP-Z100 (62, 64 and 68 ppm) exhibit slight shifts compared with that of HZSM-5 (63, 65 and 69 ppm), since the Al sites on layered structure of LSP-Z100 are extensively open and have different chemical environment, consistent with the DTBPy IR spectroscopy analysis (Fig. 2g). Thus, the super-strong Lewis acid sites (strongly bound with pyridine even at 450 °C as shown in Fig. 2f) of LSP-Z100 originate from oFTAl. Moreover, the hydride transfer between 2-methylbutane (*i*-C_5_H_12_) and deuterated hexane (C_6_D_14_) has been studied at 240 °C to demonstrate the activity of oFTAl. On LSP-Z100, *i*-C_5_H_12-*x*_D_*x*_ (*x* = 1–10), C_6_D_14–*y*_H_y_ (*y* = 1–3) were observed (Fig. 4e–h, Extended Data Figs. 3 and 4, Supplementary Figs. 20 and 21, Supplementary Tables 22 and 23 and Supplementary Discussion 5), while HZSM-5 shows little activity, indicating that the hydride abstraction and hydride transfer occur primarily on oFTAl. By extending the reaction time for HZSM-5 to 8 h, a similar deuteration level of 2-methylbutane and selectivities of products were observed, compared with that of LZP-Z100 achieved in 2 h (Supplementary Figs. 22–25), thus confirming the higher efficiency of LSP-Z100 in hydride abstraction and transfer. Controlled experiments on LSP-Z100 with decreased accessible acid sites (poisoned by DTBPy) or oFTAl sites (partially removed by ammonium hexafluorosilicate) demonstrated a notable drop in PE conversion (Supplementary Fig. 26, Supplementary Tables 10 and 24 and Supplementary Discussion 6). Also, the contribution of silanol groups with weak Lewis acidity to the conversion of PE is limited (Supplementary Fig. 27 and Supplementary Table 10, entries 5–9). These results demonstrate that oFTAl and accessible Brönsted acid sites play the primary role in the PE conversion.

### Study of reaction mechanism

**Fig. 5:**
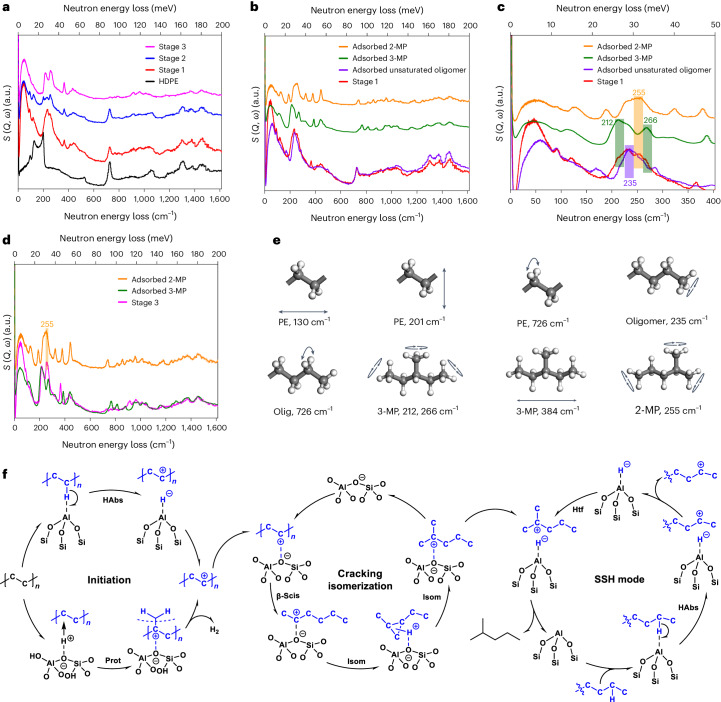
INS spectra for LSP-Z100 zeolite on the catalytic conversion of HDPE and proposed reaction mechanism. **a**, A comparison of INS spectra for solid HDPE and reacted HDPE over LSP-Z100. **b**, A comparison of INS spectra of unsaturated oligomers, adsorbed 2-methylpentane (2-MP), adsorbed 3-methylpentane (3-MP) and reacted HDPE (stage 1 of the reaction) over LSP-Z100. The INS spectrum of adsorbed unsaturated oligomer was generated by 1-butene adsorbed on LSP-Z100. The dosed 1-butene oligomerizes over LSP-Z100 and unsaturated bonds of oligomer bind to Brönsted acid site to form carbenium ions. **c**, Enlarged spectra of **b** at 0–400 cm^−^^1^. **d**, A comparison of INS spectra of adsorbed 2-MP, adsorbed 3-MP and reacted HDPE (stage 3 of the reaction) over LSP-Z100. All INS spectra shown here are after the subtraction of INS spectrum of the empty cell and zeolite. **e**, Selected vibrational modes of PE, oligomers, 3-MP and 2-MP (C, grey; H, white) observed by experiments. **f**, The proposed main reaction pathway for the conversion of PE to iso-alkane over LSP-Z100, including initiation, cracking/isomerization and hydride transfer. Habs, hydride abstraction; Prot, protonation; β-scis, β-scission; Isom, isomerization; Htf, hydrogen transfer. Source data

**Extended Data Fig. 5:**
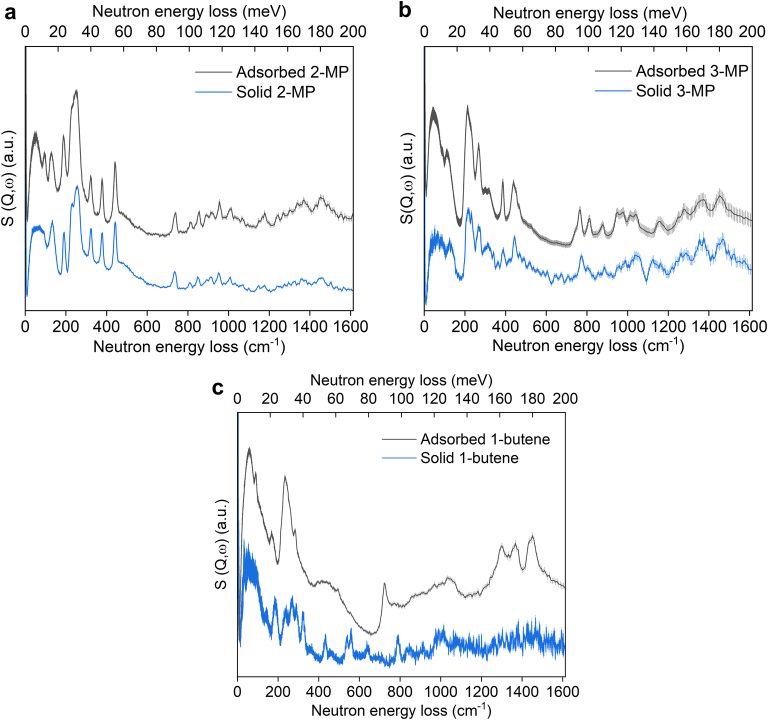
Comparison of INS spectra of adsorbed 2-methylpentane (2-MP), 3-methylpentane (3-MP) and 1-butene (unsaturated oligomer) with that of solid 2-MP, 3-MP and 1-butene. a, INS spectra of adsorbed 2-MP and solid 2-MP. b, INS spectra of adsorbed 3-MP and solid 3-MP. c, INS spectra of adsorbed 1-butene and solid 1-butene. Source data

To further understand the reaction mechanism at an atomic level*,* in situ INS (Supplementary Fig. 28 and Supplementary Discussion 7), combined with density functional theory (DFT) calculations, was employed to investigate the conversion of HDPE on LSP-Z100. The INS spectrum of activated LSP-Z100 gave a clean background with no prominent features at 0–1,600 cm^−^^1^ (Supplementary Figs. 29 and 30). The mixture of HDPE with LSP-Z100 showed a similar spectrum to that of the bare HDPE, indicating little interaction between HDPE and LSP-Z100 upon mixing at room temperature (Supplementary Fig. 30). HDPE on LSP-Z100 underwent the first catalytic conversion at 190 °C for 2 h (stage 1 of reaction, Fig.5a and Supplementary Fig. 31). In comparison with the spectrum of HDPE, the spectrum of stage 1 of the reaction showed a decrease in intensity at 130, 201 and 726 cm^−^^1^, which are assigned to the in-plane and out-of-plane skeletal stretching of HDPE, and to the methylene (–CH_2_–) rocking, respectively. This suggests the scission of C–C bonds and depolymerization of PE chains. Meanwhile, a broad feature at low energy (below 200 cm^−^^1^) appeared, suggesting that the intermediate species were disordered over the catalyst surface showing restricted translational and rotational dynamics. In addition, new peaks at 212, 235, 255, 266, 336, 440, 913 and 971 cm^−^^1^ were observed. The appearance of peaks at 212–266 cm^−^^1^ (assigned to methyl torsion, Supplementary Table 25) further confirms the cleavage of PE chains. Notably, the overall spectrum profile of stage 1 of reaction is consistent with that of adsorbed unsaturated oligomers (Fig. 5b and Supplementary Discussion 8), which is in excellent agreement with the formation of oligomers upon cracking of PE as confirmed by ^13^C NMR in the time course study. Specifically, shoulder peaks at 212, 255 and 266 cm^−^^1^ were identified as methyl torsion of 2-methylpentane and 3-methylpentane (Fig. 5c, Supplementary Fig. 32 and Supplementary Table 25) and the peaks at 336 and 440 cm^−^^1^ as the skeletal stretching modes of short alkanes (Fig. 5b, Supplementary Fig. 32 and Supplementary Table 25). This result indicates that PE was activated via hydride abstraction on oFTAl and underwent β-scission/isomerization/hydride transfer to generate iso-alkanes. Although oFTAl are able to activate C(*sp*^3^)–H of PE/alkanes, the alkanes produced can readily desorb as supported by the INS spectra of adsorbed alkanes on the LSP-Z100 (Extended Data Fig. 5a,b). In contrast, alkenes bound strongly to active sites to promote their further conversion with SSH (Extended Data Fig. 5c).

Then, the stage 2 of reaction was studied (230 °C for 1 h, Fig. 5a). The very strong peak at 235 cm^−^^1^, corresponding to the torsional mode of oligomers, decreased notably in intensity. Meanwhile, the peaks at 212 and 255 cm^−^^1^, corresponding to the methyl torsion of iso-alkanes, grew in intensity. This result suggests the rapid conversion of activated HDPE or oligomers to iso-alkanes on the zeolite via β-scission/isomerization/hydride transfer reactions. However, the peaks of HDPE (130, 201 and 726 cm^−^^1^) were still observed. Finally, the stage 3 of reaction was also investigated (240 °C for 2 h, Fig. 5a). The peaks at 235 and 726 cm^−^^1^ disappeared and the peaks at 212, 255 and 266 cm^−^^1^ increased dramatically in intensity. The spectrum of stage 3 of reaction showed notable similarity with iso-hexanes (Fig. 5d,e), confirming the efficient transformation of HDPE into iso-alkanes.

A full catalytic circle can be established (Fig. 5f). Upon adsorption on the LSP zeolite, PE is activated via hydride abstraction^38,39^ on oFTAl or via protonation on the Brönsted acid sites^40^ to give carbenium ions or carbonium ions as the initiation step. The carbonium ions formed on the Brönsted acid sites are unstable, rapidly collapsing to afford carbenium ions along with H_2_ or alkanes. The isotope-labelling reactions (Fig. 4e–h) suggest that the activation via hydride abstraction on oFTAl dominates at 240 °C. Then the activated PE (that is, the carbenium ion originated from hydride abstraction or carbonium ion collapse) binds strongly to the Brönsted acid sites and undergoes rapid β-scission and skeletal isomerization via a three-membered ring structure^41–43^, followed by intramolecular hydrogen transfer to generate stable tertiary carbenium ions. Meanwhile, oFTAl abstract hydrogen from PE or oligomers. Subsequentially, tertiary carbenium ions react with the SSH through hydride transfer to yield iso-alkanes for facile desorption and the acid sites regenerated.

## Conclusion

The LSP structures endow the materials with oFTAl sites and accessible Brönsted acid sites, which facilitate the SSH mode to activate bulk PE molecules through hydride abstraction on oFTAl sites, β-scission/isomerization on Brönsted acid sites and hydride transfer on oFTAl sites, collectively resulting in the production of gasoline with both high selectivity and yield. Moreover, LSP zeolites show excellent catalytic performance for the production of commercial-grade gasoline from both low-density and high-density PE waste (Supplementary Fig. 33), which account for about 25% of the plastic waste today. The potential profit of this route makes it economically attractive (Supplementary Discussion 9 and Supplementary Tables 26–28, the analysis is subject to uncertainties as discussed in Supplementary Information). Moreover, this PE-based route to produce gasoline has a reduced carbon emission compared with the conventional oil-based route (Supplementary Discussion 10, Supplementary Figs. 34–37, Supplementary Table 29). The integration of low energy input, inexpensive and noble metal-free and highly stable catalysts, removal of external hydrogen source and products for direct use as transportation fuels with minimized environmental impact affords a promising solution to the abatement of future plastic pollution via the ‘waste-to-chemical’ strategy.

## Methods

### Zeolite synthesis

The LSP zeolites were synthesized using the published method^44^ with modification. Aluminium isopropoxide was added into tetraethyl orthosilicate. Tetra(*n*-butyl)ammonium hydroxide was added dropwise into the mixture. Then, sodium hydroxide aqueous solution and de-ionized water were added into the mixture. The composition of the above mixture was 60 SiO_2_:0.30 Al_2_O_3_:18 TBAOH:0.75 NaOH:600 H_2_O:240 EtOH for LSP-Z100 and 60 SiO_2_:0.40 Al_2_O_3_:18 TBAOH:1.5 NaOH:600 H_2_O:240 EtOH for LSP-Z75. The sample name of LSP-ZX refers to LSP zeolite synthesized from a clear sol with the Si/Al ratio being *X*. After stirring for 12 h, the mixture for the synthesis of LSP-Z100 was sealed in an autoclave (50 ml) and heated for 88 h in a pre-heated oven at 115 °C, and that for LSP-Z75 was sealed and heated for 48 h at 120 °C. The product was centrifuged and washed by de-ionized water for several times until the pH is lower than 9. Then, the product was dried at 70 °C for 12 h and calcined at 550 °C in static air for 16 h. Ion exchange was conducted to exchange the Na^+^ sites by NH_4_^+^ using 1.0 mol l^−^^1^ ammonium chloride aqueous solution at 80 °C. The process was repeated three times, and the solid was washed and dried at 70 °C and calcined at 550 °C in static air for 4 h to produce the protonated zeolite.

For synthesis of HZSM-5, tetra(*n*-propyl)ammonium hydroxide (TPAOH), aluminium isopropoxide and de-ionized water were mixed and stirred at room temperature for 2 h. Tetraethyl orthosilicate was added and then continued to stir for another 2 h, resulting in a gel with a chemical composition of 60 SiO_2_:0.43 Al_2_O_3_:15 TPAOH:900 H_2_O. The gel was transferred and sealed in an autoclave (50 ml) and heated at 170 °C for 48 h. The product was centrifuged and washed by de-ionized water for several times until the pH is lower than 9. Then, the product was dried at 70 °C for 12 h and calcined at 550 °C in static air for 16 h. The ion exchange procedure was conducted to exchange the Na^+^ sites by NH_4_^+^ using 1.0 mol l^−1^ ammonium chloride aqueous solution at 80 °C. The process was repeated for three times, and the solid was washed and dried at 70 °C and calcined at 550 °C in static air for 4 h to produce H form zeolite. The Si/Al ratio of HZSM-5 is 60 (Supplementary Table 4) and particle size of HZSM-5 is 200 nm.

### Catalytic conversion of polyolefins

The catalytic reaction was conducted in a 10 ml Teflon-lined stainless steel autoclave. In a typical reaction, polyolefins (0.45 g) was mixed with the catalyst (0.09 g) by grinding for 5 min before loading into the autoclave. The autoclave was purged with N_2_ for three times and the pressure regulated to ambient pressure. The reactor was then sealed and heated to 240 °C in 30 min and held at this temperature for 4 h. Then, the reactor was cooled to room temperature. The gaseous product was collected with a gas bag and analysed by a GC (Agilent 8890) instrument equipped with a thermal conductivity detector (TCD), two flame ionization detectors, a HP-plot Al_2_O_3_ column, a HP PONA column, a 5 Å molecular sieve column and two hayesep Q columns. Liquid products were dissolved in CH_2_Cl_2_ and analysed by GC Agilent 8890 and GC–MS (Agilent 7890B-5977A MSD) equipped with a HP-5ms column. The standard curve method was used in quantification. Solid residue was heated at 80 °C for 12 h and analysed by TGA analyser. All long-chain species that can not be evaporated below 220 °C are considered as unconverted PE. The conversion of polyolefin and selectivity (*S*) and yield of products were calculated by the following equations:

$${\rm{Conversion}}=\Big({\left[{\rm{polyolefin}}\right]}_{0}-{\left[{\rm{polyolefin}}\right]}_{{t}}\Big)/{\left[{\rm{polyolefin}}\right]}_{0}\times 100 \%$$

$${{{S}}}_{{{i}}}={{{m}}}_{{{i}}}/\sum {{{m}}}_{{{i}}}\times 100 \%$$

$${\rm{Yield}}={\rm{conversion}}\times {{{S}}}_{{{i}}}\times 100 \%$$

where [polyolefin]_0_ and [polyolefin]_t_ denote the mass of polyolefin before and after reaction, respectively, and *m*_*i*_ denotes the mass of product *i*. The ^13^C NMR spectra of solid resides show that they are composed of unsaturated compounds and unreacted PE, and the latter account for the majority (Fig. 3b and Supplementary Fig. 6). For simplicity, all solid residues were treated as [polyolefin]_*t*_. This leads to slightly overestimated selectivity and underestimated conversion, but the yield is accurate.

The stability test of LSP-Z100 was conducted for the conversion of HDPE at 240 °C for 4 h. After each cycle of the reaction, LSP-Z100 was collected, washed, dried and calcined at 550 °C in static air for 4 h. The regenerated catalyst was used for the next cycle of catalytic conversion.

### NEXAFS

NEXAFS measurements were conducted at 4B7B station at Beijing Synchrotron Radiation Facility, and at beamline B07-B at Diamond Light Source. The samples were collected after the reaction at different stages for NEXAFS measurements. In a typical sample preparation, 0.24 g of LSP zeolite and 0.04 g of HDPE were mixed and heated at 190 °C for 2 h under 0.1 MPa N_2_ for the stage 1 of reaction. The reaction conditions were 230 °C for 1 h for stage 2 of the reaction and 240 °C for 2 h for stage 3 of the reaction. The NEXAFS spectra of all samples were collected under vacuum (or under 1 mbar He to avoid charging) at room temperature using the total electron yield method.

### SANS

SANS was measured at China Spallation Neutron Source (CSNS). In a typical experiment, LSP-Z100 powder was loaded into a quartz cuvette with 1 mm path length. The scattering intensity spectrum was collected in Ar atmosphere at room temperature. Then, LSP-Z100 and HDPE were mixed with a mass ratio of 6:1 and the mixture was loaded into a quartz cuvette in the Ar atmosphere. The temperature was raised from room temperature to 190 °C. The spectra were collected at 0.5 h, 2 h and 4 h, respectively at 190 °C. The background of Ar and quartz cuvette has been subtracted, and the scattering intensity was normalized to an absolute scale by a standard calibration procedure.

### Inelastic neutron scattering

INS spectra were recorded on the VISION spectrometer at Spallation Neutron Source, Oak Ridge National Laboratory. VISION are indirect geometry crystal analyser instruments that provide a wide dynamic range with high resolution. In a typical experiment, the catalyst (~9 g) was loaded into a flow-type stainless steel cell that can also be used as a static cell with all valves closed. The sample was heated at 450 °C (5 °C min^−1^ ramping) under He for 3 h to remove any remaining trace water before the experiment. Then, 1.5 g HDPE was mixed with activated catalyst in a glove box at room temperature. The reactions were conducted at 190, 230 and 240 °C, respectively. After each reaction, the cell was cooled for INS collection to detect the presence of possible reaction intermediates. All the INS spectra were collected after the sample was cooled and stabilized at temperatures below 15 K. INS spectra of pure solid compounds for both starting material and reaction products were collected at 5 K. The INS procedure is listed in Supplementary Fig. 28.

### DFT calculations

Simulation of the INS spectra of 1-butene, 2-methylpentane and 3-methylpentane has been conducted. Optimized geometry and vibrational frequencies of the lowest energy conformer of each molecule were calculated at the B3LYP/6-311++G(d,p) level of theory using the Gaussian suite of programs. INS spectra were obtained from calculated eigenvectors and eigenenergies using the aCLIMAX program^45^ and compared with the experimental data.

Simulation of ^13^C MAS NMR spectra of the reaction solid residue has been conducted. Predicted ^13^C chemical shift was calculated on a platform based on DFT calculation^46^. On this platform, molecular construction and visualization modules are built using Ketcher, JSmol and molview, three-dimensional conformation of two-dimensional molecular structures and the optimization of molecular structures based on Merck Molecular Force Field are performed using openbabel 2.3.1, and optimization of molecular structure based on DFT is performed using Gaussian 09 at the M06-2X/6-31G(d) level.

## Online content

Any methods, additional references, Nature Portfolio reporting summaries, source data, extended data, supplementary information, acknowledgements, peer review information; details of author contributions and competing interests; and statements of data and code availability are available at 10.1038/s41557-024-01506-z.

### Supplementary information

Supplementary Information Experiment section, Supplementary Figs. 1–37, Discussion 1–10 and Tables 1–29.

### Source data

Source Data Fig. 2 XRD and N_2_ adsorption isotherm data.

Source Data Fig. 3 Small-angle scattering data.

Source Data Fig. 4 X-ray adsorption data.

Source Data Fig. 5 Inelastic neutron scattering data.

Source Data Extended Data Fig. 3 Mass spectra data of 2-methylbtuane.

Source Data Extended Data Fig. 4 Mass spectra data of deuterated hexane.

Source Data Extended Data Fig. 5 Inelastic neutron scattering data.

## Data Availability

All data are available in the main text or Supplementary Information. Source data are provided with this paper.
