# Supplementary material for: Upcycling of polyethylene to gasoline through a self-supplied hydrogen strategy in a layered self-pillared zeolite
Source: Nat Chem. 2024 Apr 9;16(6):871–80. doi: 10.1038/s41557-024-01506-z (PMC11164678; doi:10.1038/s41557-024-01506-z)
Supplement: Supplementary file 1 — Experiment section, Supplementary Figs. 1–37, Discussion 1–10 and Tables 1–29. [file 41557_2024_1506_MOESM1_ESM.pdf]

nature chemistry

Article

https://doi.org/10.1038/s41557-024-01506-z

Upcycling of polyethylene to gasoline through a self-supplied hydrogen strategy in a layered self-pillared zeolite

In the format provided by the authors and unedited

Contents

Experimental Sections **4**
1. Materials and synthesis 4
2. Catalysts characterisation 5
3. Characterization of solid residue 6
4. Analysis of CO_2 emissions of production of fuels 6

Supplementary Discussions **7**
Supplementary Discussion 1: Py-IR and DTBPy-IR 7
Supplementary Discussion 2: Selectivity and yield of gasoline. 8
Supplementary Discussion 3: ^{13}C MAS NMR assignment. 8
Supplementary Discussion 4: Coke quantification. 9
Supplementary Discussion 5: i-C_5H_{12}/C_6D_{14} reactions 9
Supplementary Discussion 6: Controlled experiments to explore the structure-performance relationship. 10
Supplementary Discussion 7: Inelastic Neutron Scattering (INS). 10
Supplementary Discussion 8: Oligomerisation of olefin. 11
Supplementary Discussion 9: Economic analysis. 12
Supplementary Discussion 10: CO_2 emission. 12

Supplementary Figures **14**
Supplementary Fig. 1. Powder X-ray diffraction (XRD) patterns of LSP-Z100, LSP-Z75, MEL and MFI 14
Supplementary Fig. 2. N_2 adsorption of LSP-Z100, LSP-Z75 and HZSM-5 15
Supplementary Fig. 3. NH_3-TPD profiles of LSP-Z100 and HZSM-5 16
Supplementary Fig. 4. Studies of the zeolite acidity by Py-IR. 17
Supplementary Fig. 5. IR spectra before and after the adsorption of DTBPy on LSP-Z100 and HZSM-5 18
Supplementary Fig. 6. Solid-state ^{13}C NMR spectra of the reaction solid residue. 19
Supplementary Fig. 7. Solid-state ^{13}C NMR spectrum of the DCM insoluble fraction in solid residue.. 20
Supplementary Fig. 8. ^{13}C NMR spectrum of the DCM-soluble mixture extracted from HF dissolved solid residue 21
Supplementary Fig. 9. ^{1}H NMR spectrum of the DCM-soluble mixture extracted from HF dissolved solid residue 22
Supplementary Fig. 10. GD-ESI mass spectra of carbon rich products in solid residue extracted by DCM 23
Supplementary Fig. 11. GD-ESI mass spectra analysis 24
Supplementary Fig. 12. TGA plots of the mixture of HDPE and LSP-Z100, and dried solid residues after the reaction in N_2 flow 25
Supplementary Fig. 13. TGA plots of mixture of HDPE and LSP-Z100, and dried solid residues after the reaction in air flow 26
Supplementary Fig. 14. Mass fractal dimension (Dm) from SANS curves of reaction mixture 27
Supplementary Fig. 15. TGA plots of dried reaction solid residues in 4 reaction cycle 28
Supplementary Fig. 16. XRD patterns of LSP-Z100 before and after 5 recycle runs. 29
Supplementary Fig. 17. N_2 adsorption isotherms of LSP-Z100 before and after 5 recycle runs 30
Supplementary Fig. 18. NH_3-TPD profiles of LSP-Z100 before and after 5 recycle runs. 31
Supplementary Fig. 19. O *K*-edge NEXAFS spectra for LSP-Z100 before and during the reaction 32
Supplementary Fig. 20. GC traces of i-C_5H_{12}/C_6D_{14} hydride transfer reactions 33
Supplementary Fig. 21. Comparison of mass spectra of n-hexane and 2-methylbutane 34
Supplementary Fig. 22. GC traces of i-C_5H_{12}/C_6D_{14} hydride transfer reactions catalysed 35
Supplementary Fig. 23. Sectional mass spectra of 2-methylbutane after reaction on HZSM-5 for 8 hours and on LSP-Z100 for 2 hours 36
Supplementary Fig. 24. Mass spectra of 2-methylbutane after reaction on HZSM-5 for 8 hours and on LSP-Z100 for 2 hours 37
Supplementary Fig. 25. Scheme of i-C_5H_{12}/C_6D_{14} transformation reactions 38

Supplementary Fig. 26. IR spectra before and after adsorption of pyridine at variable temperatures on dealuminated LSP-Z100 (De-Al LSP-Z100). 39
Supplementary Fig. 27. IR spectra before and after adsorption of pyridine at variable temperatures on pure silicate LSP (Si-LSP) 40
Supplementary Fig. 28. Schematic view of the procedure of *operando* INS experiment and data collection...41
Supplementary Fig. 29. View of the INS spectrum for the empty catalysis cell 42
Supplementary Fig. 30. INS spectra for LSP-Z100, solid HDPE and mixture of HDPE and LSP-Z100 43
Supplementary Fig. 31. INS spectra for solid HDPE and reacted HDPE over LSP-Z100 44
Supplementary Fig. 32. Comparison of INS spectra of experimental solid 2-methylpentane (2-MP) and 3-methylpentane (3-MP) with those of calculated single molecule 45
Supplementary Fig. 33. Yields of gasoline of the catalytic conversion of reagent PE and waste PE. 46
Supplementary Fig. 34. Process flowsheet to produce fuels and petrochemicals from crude oil in the integrated refining plant with 10000 kt/a processing intake. 47
Supplementary Fig. 35. Process flowsheet to produce fuels from PE via pyrolysis in the plastic pyrolysis demonstration plant with 100 kt/a processing intake 48
Supplementary Fig. 36. Process flowsheet to produce fuels from PE via catalytic conversion. 49
Supplementary Fig. 37. CO_2 emission of liquid fuel production using strategy of this work, PE pyrolysis and crude oil refinery when converting 1 MT PE or crude oil and when producing 1 MT liquid fuel 50

Supplementary Tables 51
Supplementary Table 1. Comparison of the catalytic performance of reported strategies for the conversion of polyolefins 51
Supplementary Table 2. Textural properties determined from nitrogen sorption at 77K. 52
Supplementary Table 3. Summary of different types of Si atoms from the ^{29}Si NMR data. 53
Supplementary Table 4. Elemental analysis of zeolites 54
Supplementary Table 5. Summary of acidities of LSP-Z100 and HZSM-5 55
Supplementary Table 6. Summary of the HDPE conversion and product yields over various catalysts 56
Supplementary Table 7. Product composition of HDPE conversion catalyzed by LSP-Z100 58
Supplementary Table 8. Summary of the HDPE conversion and products selectivity over various zeolites 59
Supplementary Table 9. Prediction of gasoline Research Octane Number based on composition 60
Supplementary Table 10. Results of the HDPE conversion and gasoline yield over various zeolites.. 61
Supplementary Table 11. Molecule models and predicted ^{13}C NMR chemical shift by an on-line NMR chemical shift prediction platform 62
Supplementary Table 12. Peak assignment of solid state ^{13}C NMR spectra of reaction solid residue. 64
Supplementary Table 13. Elemental analysis of HDPE powder, mixture of HDPE and LSP-Z100 and reaction solid residue in the time course study 65
Supplementary Table 14. Mass balance in the time course study. 66
Supplementary Table 15. Carbon rich products and unreacted PE proportion in solid residue.. 67
Supplementary Table 16. Assignments for peaks in ^{1}H NMR spectrum of carbon rich products left in solid reaction residue. 68
Supplementary Table 17. Assignments for various GD-ESI-MS series in the carbon rich products left in solid reaction residue. 69
Supplementary Table 18. Summary of the product distribution of Reactions 1-4 over LSP-Z100 70
Supplementary Table 19. Elemental analysis of HDPE powder, mixture of HDPE and LSP-Z100 and reaction solid residue in reaction 1-4 71
Supplementary Table 20. Quantification of coke after every reaction by TGA analysis. 72
Supplementary Table 21. Peak assignment of solid state ^{31}P NMR spectra of trimethylphosphine oxide (TMPO) adsorbed on zeolites. 73
Supplementary Table 22. Mass of ions of 2-methylbutane from GC-MS. 74
Supplementary Table 23. Mass of ions of hexane from GC-MS. 75
Supplementary Table 24. Summary of acidities before and after dealumination of LSP-Z100 76
Supplementary Table 25. Peak assignment of the INS spectra 77
Supplementary Table 26. Predicted total capital investment 79

Supplementary Table 27. Predicted total variable operational costs 81
Supplementary Table 28. Summary economic analysis 83
Supplementary Table 29. CO_2 emission of liquid fuel production 84

Supplementary References 86

Experimental Sections

1. Materials and synthesis

Tetraethyl orthosilicate (TEOS, >99%), zirconia (IV) hydroxide (97%), tetra(n-butyl)ammonium bromide (TBABr, >98.0%) and hydrofluoric acid solution (HF, 48-51 %) were purchased from Sigma Aldrich. Aluminium isopropoxide (99.99%) and sodium hydroxide aqueous solution (50 *wt.*%) were obtained from ACROS. Tetra(n-butyl)ammonium hydroxide (TBAOH, 40 *wt.*%), tetra(n-propyl)ammonium hydroxide (TPAOH, 25 *wt.*%), Ru/C, chloroplatinic acid (8 wt % in H_2O), urea (99.5%) and Ammonium hexafluorosilicate (AHFS) were obtained from Innochem. Ammonium chloride (99.5%) was obtained from Amethyst Chemicals. HY (Si/Al=20/1), HY (Si/Al=50/1), ultra-stable HY (Si/Al=5.5/1), and mesoporous HY (Si/Al=5/1) were provided by Nankai University Catalyst Co. Ltd. α-Al_2O_3 (99.9%), ammonium metatungstate hydrate (99.5%), platinum (1% on γ-Al_2O_3, reduced) and N-butylpyridinium chloride (98%) were obtained from Alfa Aesar. Acid boric solution (4 %) was obtained from TCI. SBA-15 (H^+ form, Si/Al=20/1), silicon dioxide (99.5% metals basis, 15 nm) and sodium bicarbonate ($NaHCO_3$) were purchased from Aladdin. MCM-41 (H^+ form, Si/Al=12.5/1) was provided by XFNANO. Reagent high-density polyethylene (HDPE) powder (Mw~80,000) was purchased from Macklin. Reagent low-density polyethylene (LDPE) powder was purchased from Alfa Aesar. Smashed powder of recycled HDPE (HDPE-r) and LDPE (LDPE-r) plastic wastes were purchased from Dongguan Zhongcheng Plastic Material Business Company. Aluminum chloride (99.99%) was purchased from Lanzhou Yulu Fine Chemical Co., Ltd. Fresh and spent FCC catalysts were provided by Sinopec Sichuan Petroleum Company. Short b-axis ZSM-5, [C_4Py]Cl-$AlCl_3$ and Pt/WO_3/ZrO_2 were synthesised following the literature method reported in Ref.[1], Ref.[2] and Ref.[3], respectively. HZSM-5-micron with a particle size of 4 micrometres and Si/Al ratio of 62 was synthesised from a precursor gel composed of 60 SiO_2: 0.43 Al_2O_3: 6 TPAOH: 3.8 NaOH: 2138 H_2O and hydrothermal treatment at 180 °C for 24 hours. HZSM-11 with a particle size of 5 micrometres and Si/Al ratio of 62 was synthesised from a precursor gel composed of 60 SiO_2: 0.40 Al_2O_3: 18 TBABr: 6 Na2O: 2520 H_2O and hydrothermal treatment at 140 °C for 72 hours.

The adsorption of trimethylhosphine oxide and 2,6-di-*tert*-butylpyridine (DTBPy) adsorbed LSP-Z100 was conducted following this procedure. LSP-Z100 was activated at 550 ℃ under dynamic vacuum for 2 hours in a Schlenk tube. Then the Schlenk tube was sealed and cooled to room temperature. Probes (DTBPy or CH_2Cl_2

diluted TMPO) were added into Schlenk tube in a glovebox. The tube was then sealed and further heated at 150 ℃ to promote the adsorption of DTBPy in a uniform manner. The tube containing TMPO adsorbed LSP-Z100 was then vacuumed at 80 ℃ to remove CH_2Cl_2 and sealed and heated at 180 ℃ for 2 hours. Then, both Schlenk tubes were heated at 240 ℃ under vacuum for 2 hours.

Dealumination of Al species in LSP-Z100 was conducted by dispersing zeolites in AHFS solution at a concentration of 1g/50 mL with an AHFS: Al ratio of 2. The mixture was heated at 90 °C for 4 hours with stirring, and then centrifugated and washed by water for three times. The collected solid was dried at 100 °C overnight and then calcined at 550 °C for 6 hours in air with a rate of 5 K/min.

2. Catalyst characterisation

The morphology and size of the crystallites were studied by transmission electron microscopy (TEM) on a JEOL JEM-F200 microscope. Powder X-ray diffraction (XRD) patterns were recorded on a Rigaku D/max 2500 X-ray diffractometer (40 kV and 20 mA) using Cu Kα radiation (λ = 1.54056 Å). N_2 adsorption was carried out at 77 K on a Micromeritics ASAP 2020HD88 instrument after activating the samples for 12 hours under dynamic vacuum at 300 ℃.

Solid-state ^{1}H-^{29}Si Cross Polarisation / Magic Angle Spinning Nuclear Magnetic Resonance (CP/MAS NMR) spectra were recorded with a frequency of 119.10 MHz, a spinning rate of 8.00 kHz, and a recycling delay of 3.00 s on a Bruker NEO 600 WB NMR Spectrometer. Solid-state ^{27}Al One-Pulse NMR spectra were recorded with a frequency of 104.01 MHz, a spinning rate of 12.00 kHz, and a recycling delay of 0.50 s on a Bruker Avance III 400 NMR spectrometer. Solid-State ^{1}H-^{13}C Cross Polarisation / Total Suppression of Sidebands (CP/TOSS) NMR spectra of the reaction solid residue were recorded with a frequency of 100.38 MHz, a spinning rate of 8.00 kHz, and a recycling delay of 2.00 s.

The acidity of the zeolites was measured by temperature-programmed desorption of ammonia (NH_3-TPD) on a Micromeritics AUTOCHEM II 2920 instrument equipped with a thermal conductivity detector (TCD). Typically, 100 mg of the catalyst was pre-treated in a stream of helium at 550 ℃ for 3 hours. The adsorption of NH_3 was carried out at 50 ℃ for 1 hour. The catalyst was then flushed with a stream of helium at 100 ℃ for 2 hours to remove physisorbed NH_3 molecules. Then the TCD signal was recorded at a heating rate of 10 $℃\cdot min^{-1}$

from 100 to 550 ℃. Pyridine adsorbed Infrared (Py-IR) spectra and 2,6-di-*tert*-butylpyridine adsorbed Infrared (DTBPy-IR) spectra were collected on a Nicolet NEXUS-FTIR-670 spectrometer. The samples were activated at 450 ℃ for 1 hour under dynamic vacuum. Adsorption of pyridine or DTBPy was carried out by exposing the desolvated sample wafer to DTBPy vapour at 50 ℃ for 0.5 hour. The samples were outgassed for 0.5 hour upon heating at 150, 200, 250, 350, and 450 °C. Then, the spectra were recorded.

3. characterisation of solid residue

Thermogravimetric analysis (TGA) of the used catalysts and solid residues after the reaction were carried out on a PerkinElmer TGA4000 with a flow of N_2 (20 mL min^{-1}) with a ramp rate of 5 ℃·min^{-1} up to 400 ℃ and then switching to a flow of air (20 mL min^{-1}) with a ramp rate of 5 ℃·min^{-1} up to 800 ℃. Elemental analysis was conducted in Thermo Flash Smart of Thermo Scientific instrument.

To dissolve the zeolite and separate carbon rich products and unreacted PE, the solid residue (0.18 – 0.36 g) was immersed in 10 mL HF solution at room temperature for 20 minutes. Then, HF was neutralised by acid boric solution and $NaHCO_3$ solution. The carbon rich products were extracted and separated by dichloride methane (DCM), and the unreacted PE is left insoluble as solid. The insoluble unreacted PE and DCM soluble carbon rich products were dried at 80 °C overnight and weighed respectively, and then characterised by various techniques. Glow discharge–electrospray ionization (GD-ESI) mass spectra were obtained on a Bruker Compact instrument with atmospheric pressure inlet system.

4. Analysis of CO_2 emissions of production of fuels

The carbon emission of production of liquid fuels via PE pyrolysis and crude oil-based refinery is calculated based upon standard protocols of the IPCC Guidelines for National Greenhouse Gas Emissions Inventories and the GB/T 2589-2020 General rules for calculation of the comprehensive energy consumption[4,5]. Typically, to convert 1 kg waste polyethylene via our strategy, the energy consumption of the collection and cleaning of waste plastics is 29.7 kJ (calculated based on fuel gas, the same below), and that of catalytic conversion is 8,640 kJ. The estimation of latter is based on the results of 4 consecutive cycles (Fig. 3g), where PE is fed every 4 h over 4 cycles. The indirect emissions caused by the use of catalysts have an emission factor

of 18.87g/g (actual production data). The carbon emission factor of fuel gas (calculated as CO_2) is 0.0561 g/kg. The CO_2 emissions of plastic pyrolysis and petroleum refinery are calculated based on the data of the plastic pyrolysis demonstration plant with 100 kt/a processing intake and the integrated refining plant with 10000 kt/a processing intake, respectively, of SINOPEC Research Institute of Petroleum Processing Co., LTD. The CO_2 emissions of converting 1 kg raw materials and yielding 1 kg liquid fuel are compared to evaluate the potential advantage of our strategy to produce liquid fuels.

Supplementary Discussion

Supplementary Discussion 1: Py-IR and DTBPy-IR

In the Py-IR spectra of the zeolites, the peaks at 1454 and 1545 cm^{-1} are assigned to the coordinatively bound pyridine molecules on Lewis acid sites and pyridinium ion on Brönsted acid sites, respectively[6]. As shown in Supplementary Fig. 4, the peak intensities related to both Lewis acid sites and Brönsted acid sites decrease upon heating under vacuum. The result shows that LSP-Z100 exhibits more Lewis acid sites than HZSM-5 (Supplementary Fig. 4, Supplementary Table 5).

In the DTBPy-IR spectra of the zeolites, the peaks at 3740 and 3610 cm^{-1} are assigned to the stretching mode of silanol groups and -OH of Brönsted acid groups of zeolites, respectively. The peak at 3370 cm^{-1} is assigned to stretching mode of the protonated amine group (≡N-H^+) that is related to Brönsted acid sites[7]. The peaks at 1616 and 1530 cm^{-1} are assigned to Brönsted, Lewis or both types of acid centers[7]. The peak at 3610 cm^{-1} of the bare LSP-Z100 zeolite almost disappeared after adsorption of DTBPy molecules (after vacuum treatment at 150 ℃), and new peaks appear at 3370, 1616 and 1530 cm^{-1}, indicating that most of the acid sites of the LSP-Z100 zeolite are accessible to the probe molecule DTBPy (Fig. 2g and Supplementary Fig. 5). In contrast, the peak at 3610 cm^{-1} of bare HZSM-5 only decreased slightly in intensity upon adsorption of DTBPy (after vacuum treatment at 150 ℃), and the peaks at 3370, 1616 and 1530 cm^{-1} increased slightly, suggesting that few acid sites of HZSM-5 are accessible to DTBPy except for those residing on the external surface of bulk particles. The comparison of the DTBPy-IR spectra of LSP-Z100 and HZSM-5 shows that the layered structure and mesopores greatly facilitate the interaction of substrates and active sites of the catalyst.

Supplementary Discussion 2: Selectivity and yield of gasoline

Compared with noble metal catalysts (Supplementary Table 1), the selectivity of gasoline over LSP zeolite is improved notably. Under mild reaction conditions and pressure of hydrogen, noble metals exhibit exceptional activity in cleaving C-C bonds through hydrogenolysis. However, preferential adsorption of end groups, indiscriminate C-C bond cleavage, and cleavage of multiple C-C bonds on noble metals lead to the substantial production of C_1-C_3 compounds.[8–10] In contrast, on LSP zeolite, C-C bonds cleavage occurs through a β-scission mechanism on acidic sites. The generation of C_1-C_3 compounds involves primary or secondary carbenium ions, which are unstable and require high reaction temperature. On the other hand, the formation of C_{4+} compounds, particularly branched C_{4+} compounds through A-type (tertiary–tertiary) or B-type (secondary–tertiary) β-scission, is energetically more favourable[11,12] and rapidly occurs at mild reaction temperatures. Additionally, the layered topologies of the MFI and MEI in LSP zeolite, featuring approximately 5.6 Å micropores, facilitate shape-selective production of hydrocarbons with specific ranges of chain length. Consequently, the gasoline range hydrocarbons are produced selectively on LSP zeolite at mild reaction temperatures.

The layered self-pillared structures of LSP zeolite endow the extensively open acid sites, such as oFTAl sites and accessible Brönsted acid sites (Fig. 2g, Figs. 4c,d). These sites proficiently activate C-H bonds (Figs. 4e-h) and crack C-C bonds (Fig. 5), respectively, under mild reaction temperatures, leading to enhanced PE conversion compared with traditional 3D-structured zeolites (Supplementary Table 8). These have been further validated by poisoning experiments (Supplementary Table 10, entries 3 and 8) and dealumination experiments (Supplementary Fig. 26, Supplementary Table 24, Supplementary Table 10, entries 4 and 8). Besides, silanol groups (Supplementary Fig. 27, Supplementary Table 10, entries 5-9) and shortened diffusion path of LSP zeolite also contribute to the production of gasoline.

Supplementary Discussion 3: ^{13}C MAS NMR assignment

To assign peaks in the solid-state ^{13}C MAS NMR spectra and determine the structure of reaction solid residue, theoretical calculations were carried out using the prediction platform in ref.[13]. It is assumed that the reaction solid residue is a mixture of molecules in the range of polymer to oligomer. We proposed several

oligomers with certain groups (branched chain, aliphatic ring, double bond and aromatic rings) (Supplementary Table 12). This platform predicts the chemical shift of each specific carbon in a given oligomer. By integrating the sets of points generated from all the predicted structures and fitting them into a smooth curve, the simulated curve in Fig. 3b is generated. The simulated curve shows an excellent agreement with experimental curve, confirming the main components in the solid residue. Besides, guided by the predicted chemical shift in each structure, the peak assignments of solid state ^{13}C MAS NMR spectra are listed in Supplementary Table 12.

Supplementary Discussion 4: Coke quantification

TGA analysis was conducted to quantify the coke generated in the reactions. The conventional method to quantify coke on zeolites is by analysing weight loss at temperature ramp in an air flow. However, the weight loss of uncracked oligomers and coke in the reaction residue of this work are not discernible (Supplementary Fig. 13). Thus, we adopted a temperature-programmed method with a flow of N_2 (20 mL min^{-1}) and a ramp rate of 5 ℃·min^{-1} up to 400 ℃ and then switching to a flow of air (20 mL min^{-1}) and a ramp rate of 5 ℃·min^{-1} up to 800 ℃. The weight loss in the range of 30 to 200 ℃ and 200 to 400 ℃ under N_2 flow is assigned to reaction products and uncracked oligomers, respectively (Supplementary Figs. 12 and 15). The weight loss in the range of 400 to 800 ℃ under air flow is assigned to hard coke generated in the reaction[14].

Supplementary Discussion 5: i-C_5H_{12}/C_6D_{14} reactions

Hydride transfer reactions between 2-methylbutane (i-C_5H_{12}) and deuterated hexane (C_6D_{14}) were conducted to investigate the activation of C-H bonds by LSP-Z100. The oFTAl sites of LSP-Z100 grab hydride from saturated i-C_5H_{12} and C_6D_{14} and transfer it between activated alkanes. Thus, i-$C_5H_{12-x}D_x$ (x=1-10) and $C_6D_{14-y}H_y$ (y=1-3) were observed on LSP-Z100 (Figs. 4f-h, Extended Data Figs. 3 and 4, Supplementary Figs. 20-25, Supplementary Tables 22 and 23). The reaction pathway is shown in Supplementary Fig. 25. In addition to the hydride transfer reaction after C-H activation, isomerisation and dehydrogenation occurred efficiently on LSP-Z100. i-C_5H_{10} and C_6D_{12} are generated because dehydrogenation underwent oligomerisation and further cracked into C_4-C_8 hydrocarbons with different deuterated degrees. In contrast, i-C_5H_{12} and C_6D_{14} activated on HZSM-5 show only a small degree of hydride transfer, dehydrogenation or cracking products (Supplementary

Fig. 20). The i-C_5H_{12}/C_6D_{14} reaction confirmed the superior activity of LSP-Z100 to activate C-H bonds and catalyse the hydride transfer, dehydrogenation and β-scission.

Supplementary Discussion 6: Controlled experiments to explore the structure-performance relationship

Poisoning experiments using DTBPy confirmed the importance of accessible acid sites (Supplementary Table 10, entries 3 and 8). PE conversion dropped from 81.8% to 46.2% on LSP-Z100 upon partial poisoning of accessible acid sites by DTBPy. Dealuminated LSP-Z100, in which Lewis acid sites such as oFTAl sites were partially removed, shows a significant drop of PE conversion from 81.8% to 58.9% (Supplementary Table 10, entries 4 and 8, Supplementary Fig. 26), corroborating that the Lewis acid sites originated from oFTAL sites are key to the initiation of C-H bond[15] and promotes hydrogen transfer reaction[16].

The effect of silanol groups has been studied by adding pure silicate LSP (Si-LSP) which possessed silanol group with weak Lewis acid sites (Supplementary Fig. 27). By adding pure silicate LSP (Si-LSP) along with LSP-Z100, increased amount of silanol groups participate into PE conversion. It is observed that silanol groups can promote PE conversion at the early stage and facilitate aromatisation at the later stage of reaction. However, the promotion of silanol group on PE conversion is limited (Supplementary Table 10, entries 5-9). Thus, the controlled experiments further demonstrated that the main active sites for PE conversion are oFTAl sites and accessible Brönsted acid sites.

The importance of shortened diffusion path is demonstrated by controlled experiments on HZSM-5 with the similar Si/Al ratio and different particle size. The PE conversion dropped from 35.1% on 200 nanometres HZSM-5 to 16.2% on 4 micrometres HZSM-5 (Supplementary Table 10, entries 1 and 2). Meanwhile, the selectivity towards alkenes is increased. With shortened diffusion path, the intermediate alkenes diffuse more rapidly within the catalyst, facilitating adequate interaction with active sites in a closed system, and thus promote the transformation of the intermediate alkenes to alkanes. HZSM-5 micro and HZSM-11 with similar crystal size and Si/Al ratio showed similar catalytic performance (Supplementary Table 10, entries 2 and 10). Thus, the introduction of MEL topology is not one of the main drivers for high PE conversion on LSP zeolites.

Supplementary Discussion 7: Inelastic Neutron Scattering (INS)

Inelastic neutron scattering (INS) is an extremely powerful neutron spectroscopy technique to study the vibrational dynamics of hydrogenous compounds by exploring the high neutron cross-section of hydrogen (82.02 barns). INS is used to study the vibrational mode of important intermediates and products during reaction process and the interaction between reactant as well as intermediates with catalyst. It has several advantages:

- INS spectroscopy is sensitive to the vibrations of hydrogen atoms, and hydrogen is ten times more visible than other elements due to its high neutron cross-section.
- The technique is not subject to any optical selection rules. All vibrations are active and, in principle, measurable.
- INS observations are not restricted to the centre of the Brillouin zone (gamma point) as is the case for optical techniques.
- INS spectra can be readily and accurately modelled: the intensities are proportional to the concentration of elements in the sample and their cross-sections, and the measured INS intensities relate straightforwardly to the associated displacements of the scattering atom. Treatment of background correction is also straightforward.
- Neutrons penetrate deeply into materials and pass readily through the walls of metal containers, making neutrons ideal to measure bulk properties of this material (in this case for 9 g catalyst)
- INS spectrometers cover the whole range of the molecular vibrational spectrum, 0-500 meV (0-4000 cm^{-1})
- INS data can be collected at below 10 K, where the thermal motion of the zeolite, polyethylene and adsorbed intermediate molecules can be significantly reduced.
- Calculation of the INS spectra by DFT vibrational analysis can be readily achieved, and DFT calculations relate directly to the INS spectra.

Supplementary Discussion 8: Oligomerisation of olefin

As shown in Extended Data Fig. 4c, the INS spectrum of adsorbed alkene (1-butene) is noticeably different from that of the experimental spectrum of non-adsorbed alkenes. This is because alkenes interact strongly with the active sites and can oligomerize over the zeolite[17,18]. Unsaturated oligomer with a carbenium

ion is generated from this process. By dosing 1-butene into the flow-type cell containing the LSP-Z100 zeolite, the INS spectra of unsaturated oligomers were obtained.

Supplementary Discussion 9: Economic analysis

A preliminary calculation on the cost of 'waste polyolefin to fuel' process is performed (based on the published catalytic data) to evaluate potential of different strategies in industry. The total capital investment and total variable operational cost of this work (Route A, 240 ℃, LSP zeolite alone) and Route B and C have been estimated and compared (Supplementary Tables 26-28). The variation of total capital investment among different routes is caused by catalyst performance (reaction temperature and time) and demands for functional equipment (e.g., explosion-proof equipment) and separation of products. The variation of the total variable operational cost is based upon the cost of catalyst (e.g., noble metal) and additives (e.g., hydrogen). This economic analysis is subject to changes of external conditions, costs and feedstock price and the impact of mass and heat transfer at an industrial scale cannot be included. Thus, this simple economic analysis is only for comparison among the reported catalytic processes on a laboratory scale.

The LSP catalyst used in this work possess excellent catalytic performance and is capable of catalysing PE conversion to gasoline (selectivity>99%) under mild conditions and within short reaction time. Besides, the cost of LSP catalysts is cheap and no other expenditure for additives is required. Therefore, Route A has been demonstrated as a highly profitable strategy (Supplementary Table 28) among all routes with the profit as high as 1043.6 $/MT gasoline. This route, therefore, demonstrates great potential to for commercialisation.

Supplementary Discussion 10: CO_2 emission

The CO_2 emissions of polyethylene pyrolysis and crude oil-based refinery are estimated to demonstrate the reduced environmental impact of our strategy (Supplementary Figs. 34-37, Supplementary Table. 29). Although the conversion of PE wastes to gasoline requires higher energy input than the mature oil-based route (566.5 kg $_{CO2}$·MT $_{PE}^{-1}$ vs 323.0 kg $_{CO2}$·MT $_{crude\ oil}^{-1}$, respectively), the huge carbon emission of extraction of the crude oil (332.0 kg $_{CO2}$·MT $_{crude\ oil}^{-1}$) renders the oil-based routes more carbon-intensive (655.0 kg $_{CO2}$·MT $_{crude\ oil}^{-}$

^{1})[19]. Therefore, compared with the oil-based route, the PE-based route for gasoline production is advantageous in terms of reduced carbon emission (Supplementary Fig. 37, Supplementary Table. 29). In addition, this strategy provides a vital and economic-viable solution to mitigate the plastic pollution[20,21].

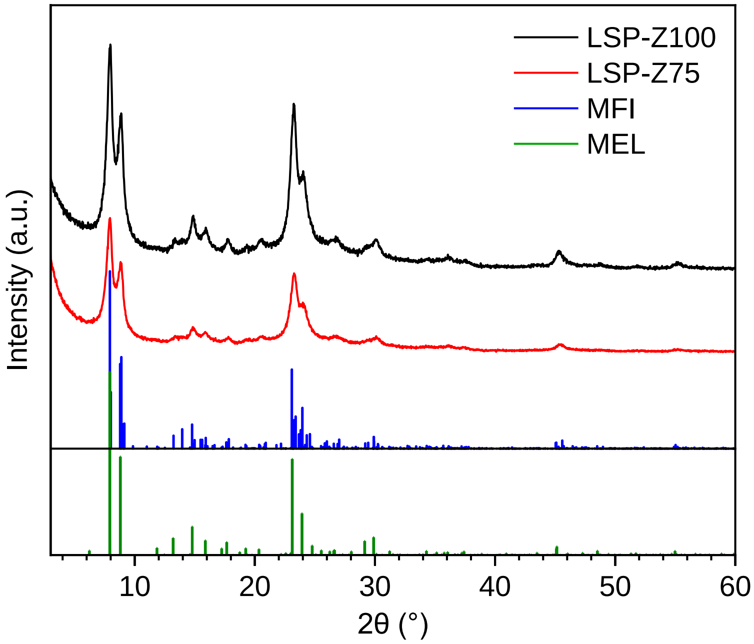

Supplementary Fig. 1 | Powder X-ray diffraction (XRD) patterns of LSP-Z100, LSP-Z75, MEL and MFI. The XRD patterns revealed that the LSP zeolites are MFI/MEL intergrowth. XRD patterns of MEL and MFI were drawn from the Database of Zeolite Structures[22].

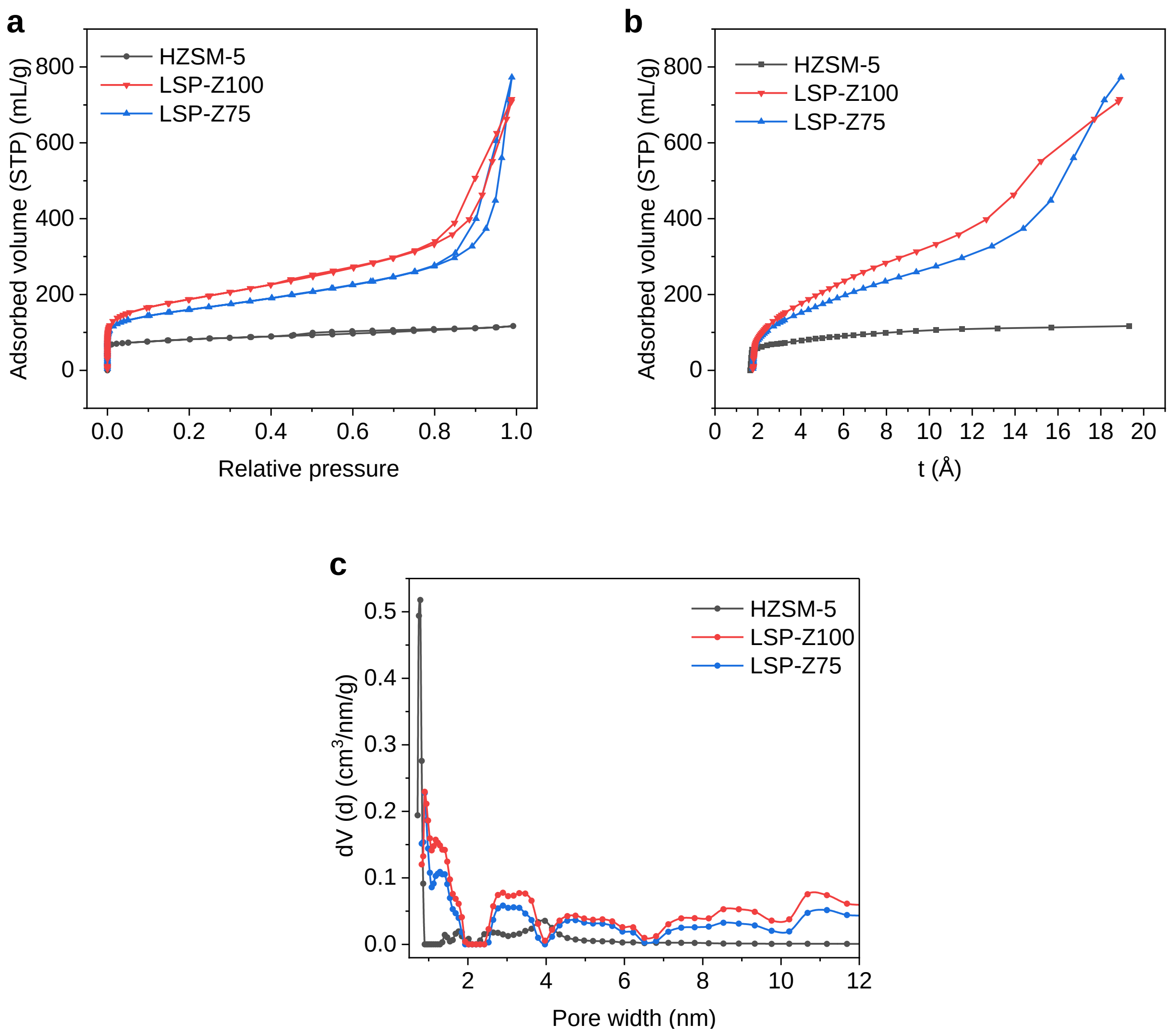

Supplementary Fig. 2 | N_2 adsorption of LSP-Z100, LSP-Z75 and HZSM-5. (**a**) N_2 adsorption/desorption isotherms. (**b**) t-plot profile. (**c**) Non-local DFT pore size distributions.

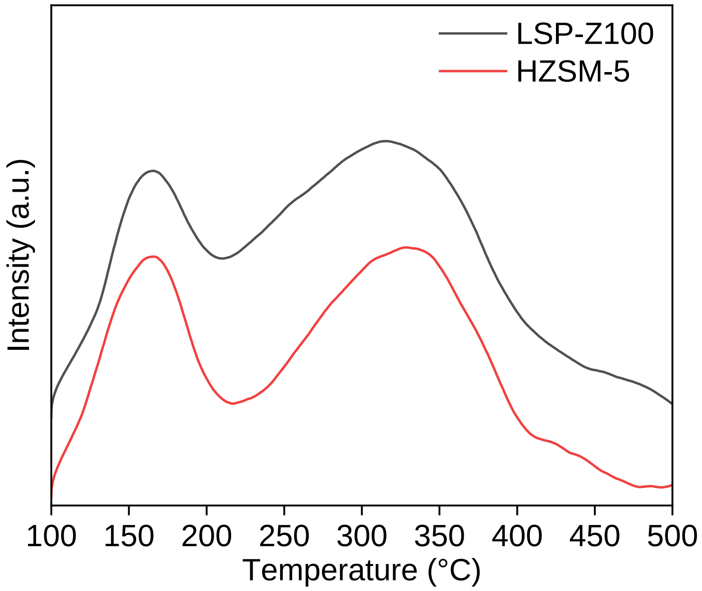

Supplementary Fig. 3 | NH_3-TPD profiles of LSP-Z100 and HZSM-5, showing similar acidity of both samples.

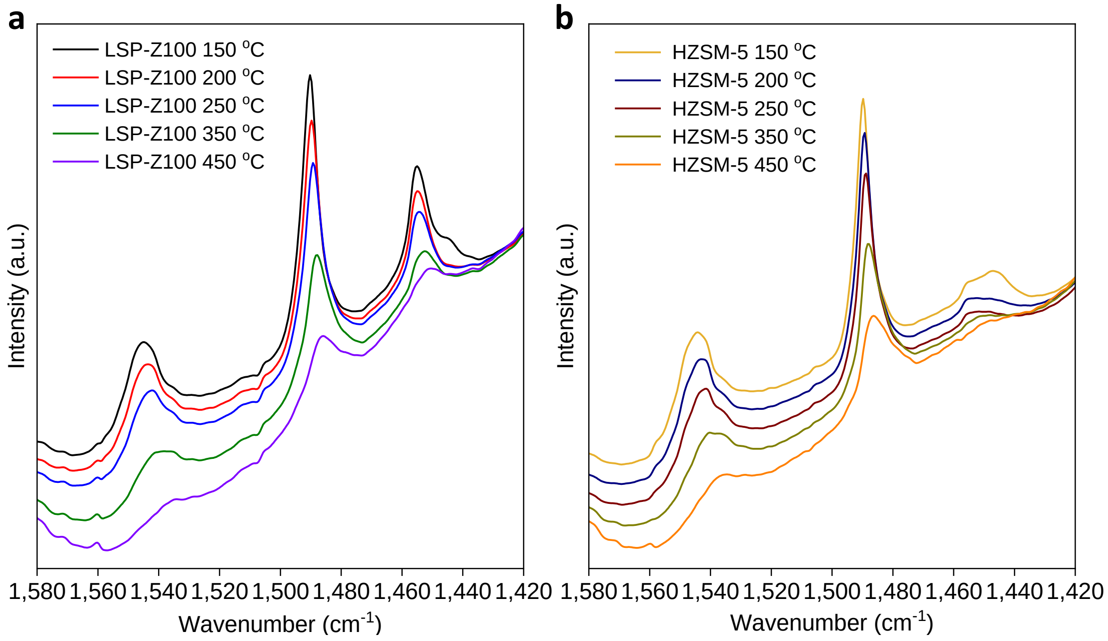

Supplementary Fig. 4 | Studies of the zeolite acidity by Py-IR. Py-IR spectra of LSP-Z100 (**a**) and HZSM-5 (**b**) after vacuum treatment at variable temperatures.

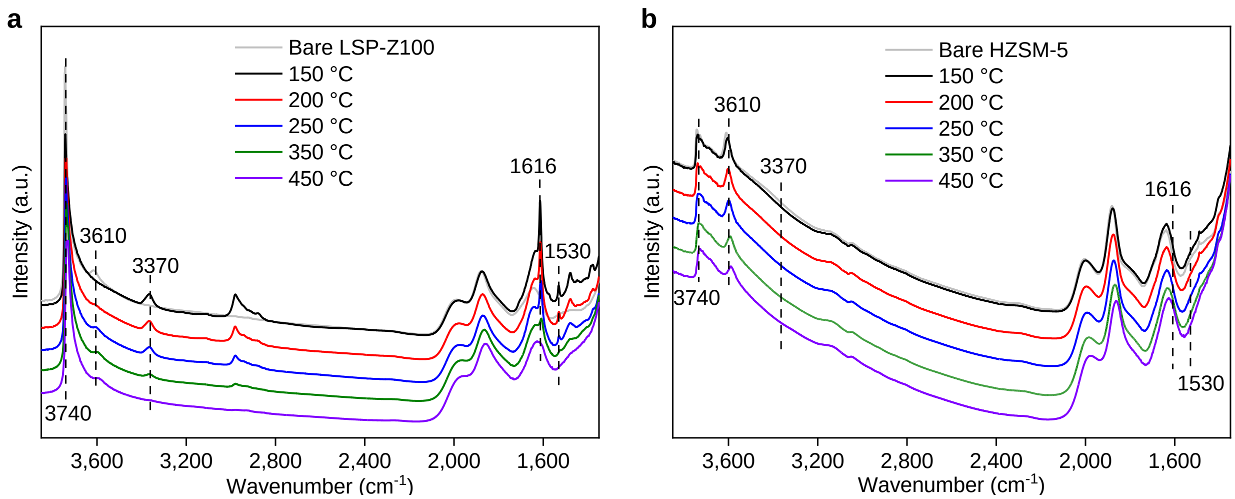

Supplementary Fig. 5 | IR spectra before and after the adsorption of DTBPy on LSP-Z100 (**a**) and HZSM-5 (**b**).

The post-adsorption samples were treated under vacuum at variable temperatures.

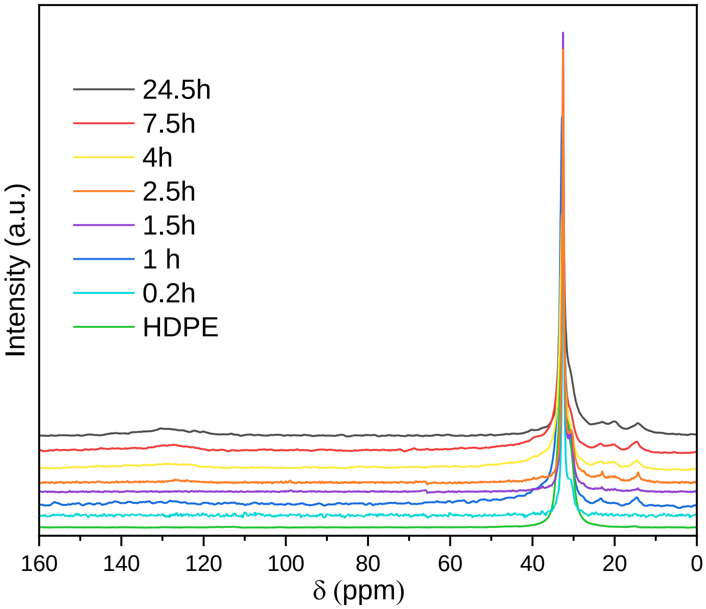

Supplementary Fig. 6 | Solid-state ^{13}C NMR spectra of the reaction solid residue. The main composition of reaction residue is methylene group (δ=30-35) rather than unsaturated carbons (e.g. olefins and aromatics) by the area semiquantitative method. This result also confirms little coke formation in reaction.

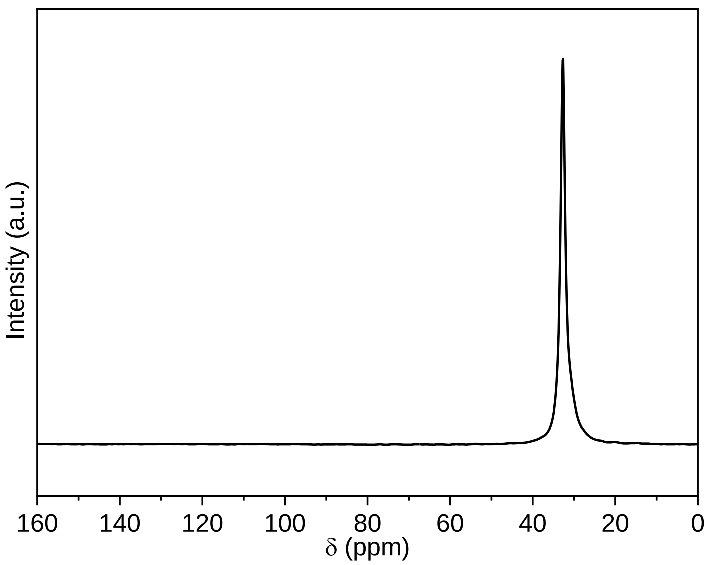

Supplementary Fig. 7 | Solid-state ^{13}C NMR spectrum of the DCM insoluble fraction in solid residue.

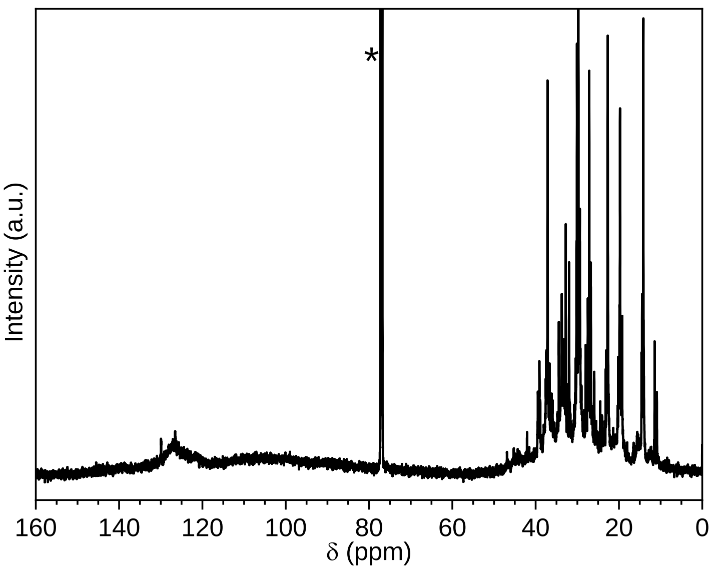

Supplementary Fig. 8 | ^{13}C NMR spectrum of the DCM-soluble mixture extracted from HF dissolved solid residue. The peak at 77 ppm marked with * is assigned to solvent ($CDCl_3$).

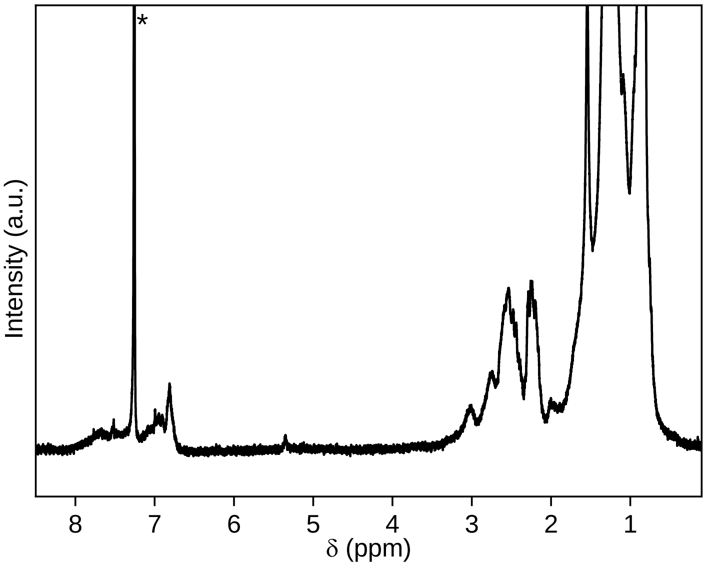

Supplementary Fig. 9 | ^{1}H NMR spectrum of the DCM-soluble mixture extracted from HF dissolved solid residue. The peak at 7.26 ppm marked with * is assigned to solvent ($CDCl_3$).

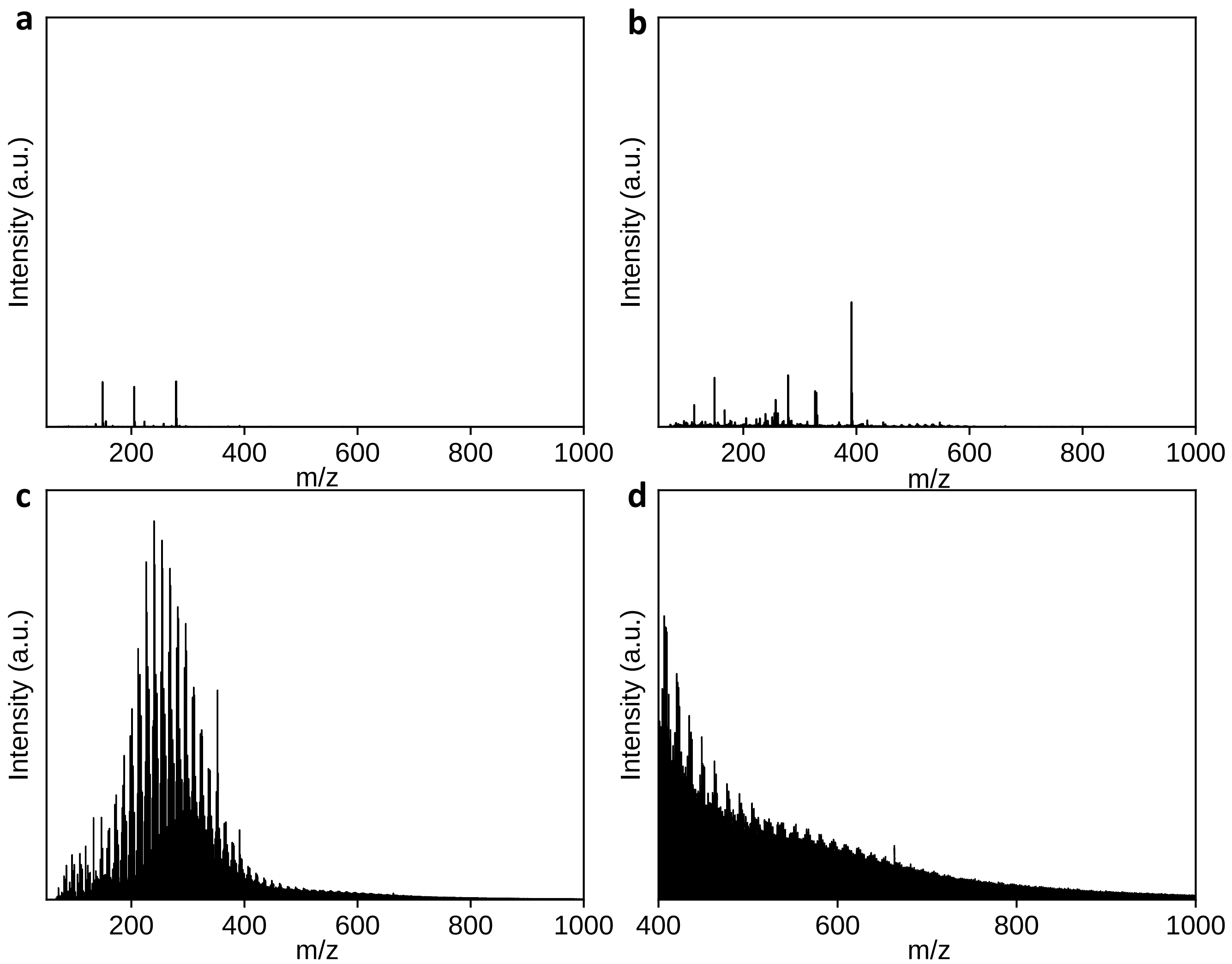

Supplementary Fig. 10 | GD-ESI mass spectra of reaction gas argon (a), solvent dichloromethane inletting (b), carbon rich products in solid residue extracted by DCM (c) and enlarged spectrum of (c) in the m/z range of 400-1000 (d). The peak in (a) and (b) is due to the random ionisation of impurities in air and plasticiser in inlet system which dissolved by DCM.

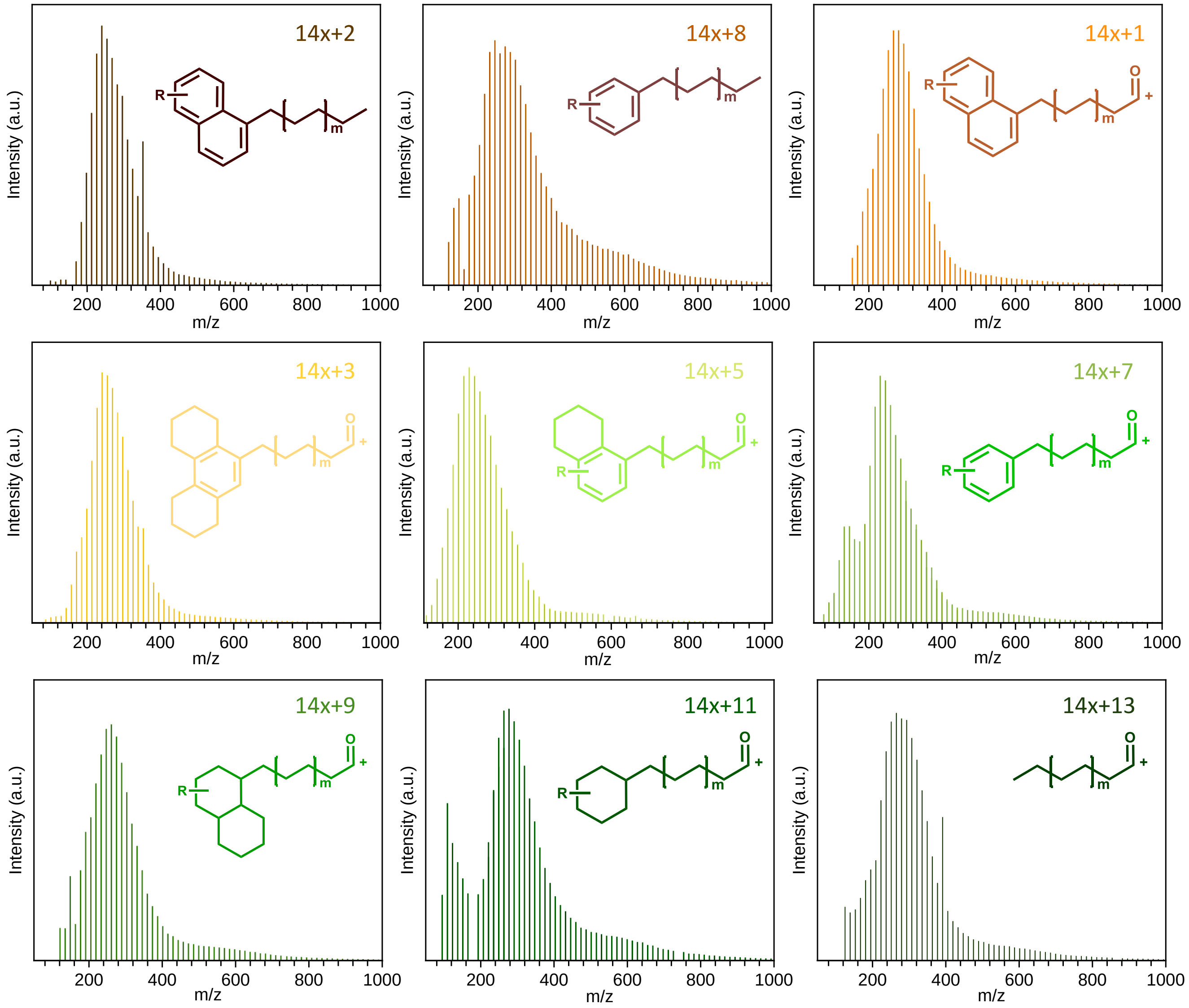

Supplementary Fig. 11 | GD-ESI mass spectra analysis. *m* denotes the number of repetitive -CH_2-CH_2- pieces in oligomers. *x* denotes any positive integer, and 14 is the weight of CH_2 unit. For example, tetracontane ($C_{40}H_{82}$) is one of the components in oligomers. In GS-ESI inletting process, it is oxidised into $C_{40}H_{79}O^+$, and the m/z of the generated ion is 14*40+13, which belongs to 14x+13 peak series.

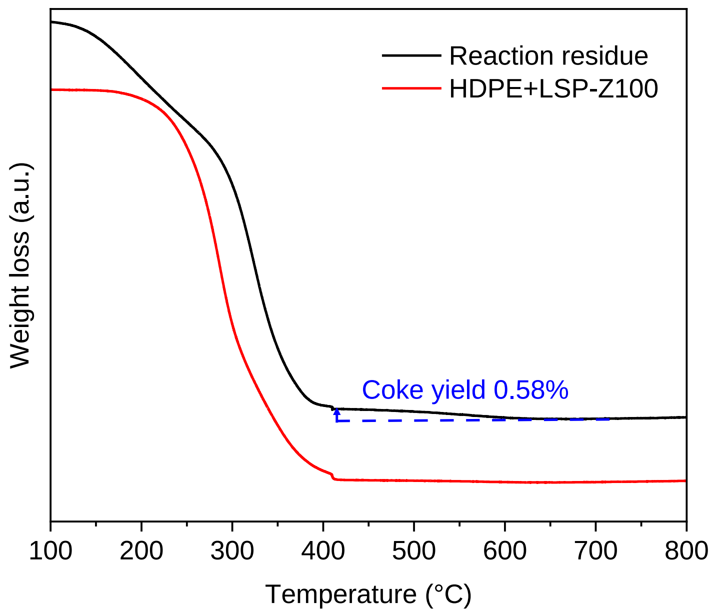

Supplementary Fig. 12 | TGA plots of the mixture of HDPE and LSP-Z100 (HDPE : LSP-Z100 = 1:1), and dried solid residues after the reaction. The measurement is conducted in nitrogen flow.

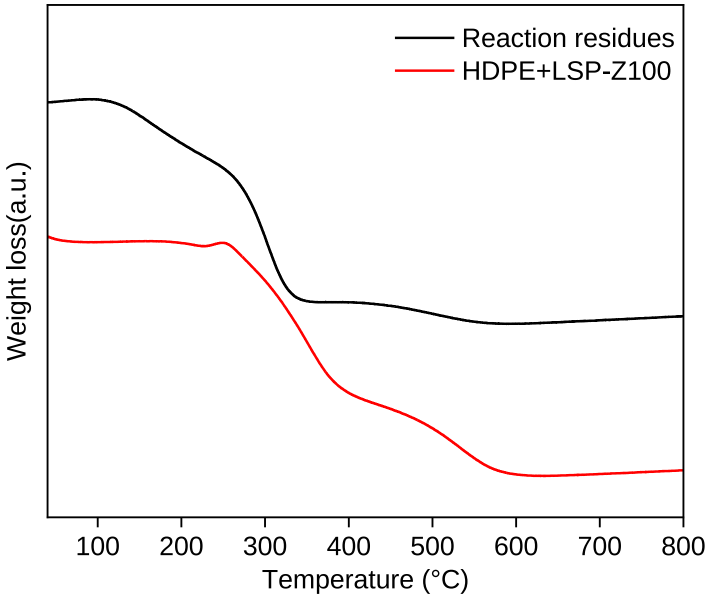

Supplementary Fig. 13 | TGA plots of mixture of HDPE and LSP-Z100 (HDPE : LSP-Z100 = 1:1), and dried solid residues after the reaction. The measurement is conducted in air flow.

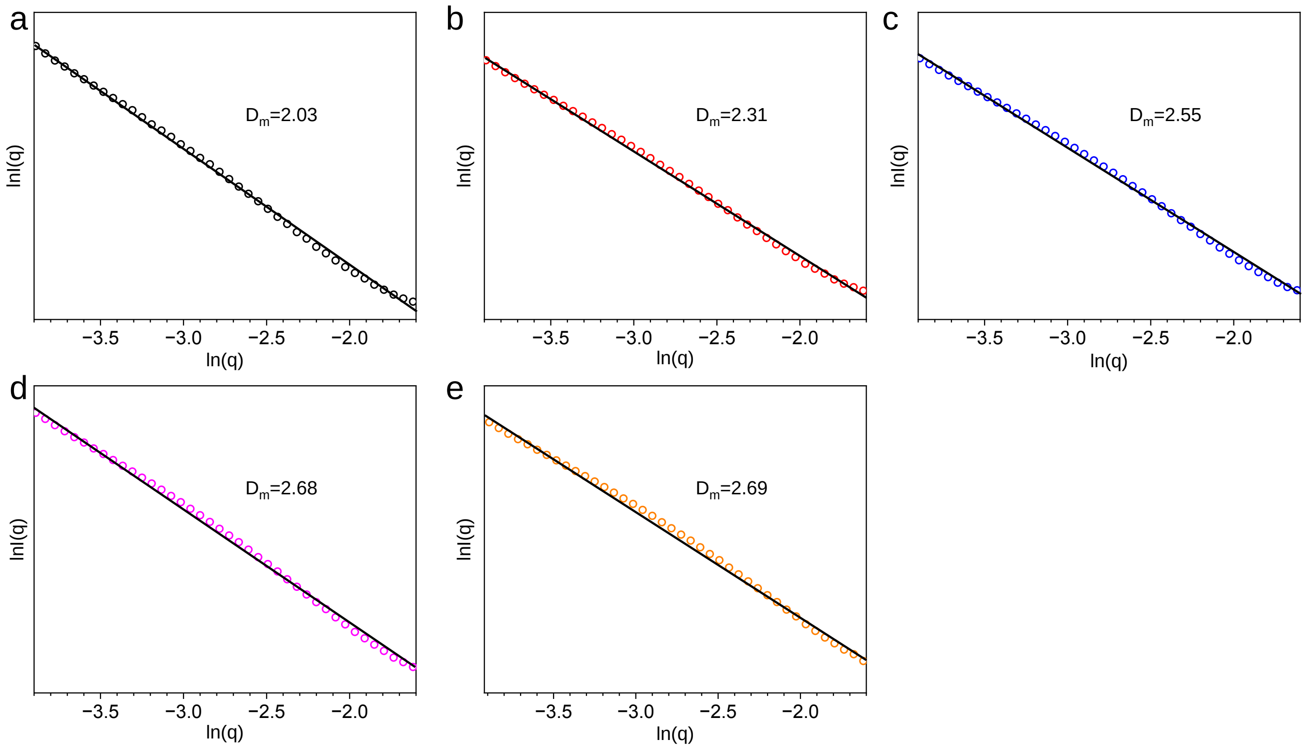

Supplementary Fig. 14 | Mass fractal dimension (D_m) from SANS curves of reaction mixture of LSP-Z100 and HDPE at room temperature (**a**), reaction mixture collected after being heated for 0.5h (**b**), 2h (**c**), 4h (**d**), and pure LSP-Z100 powder (**e**).

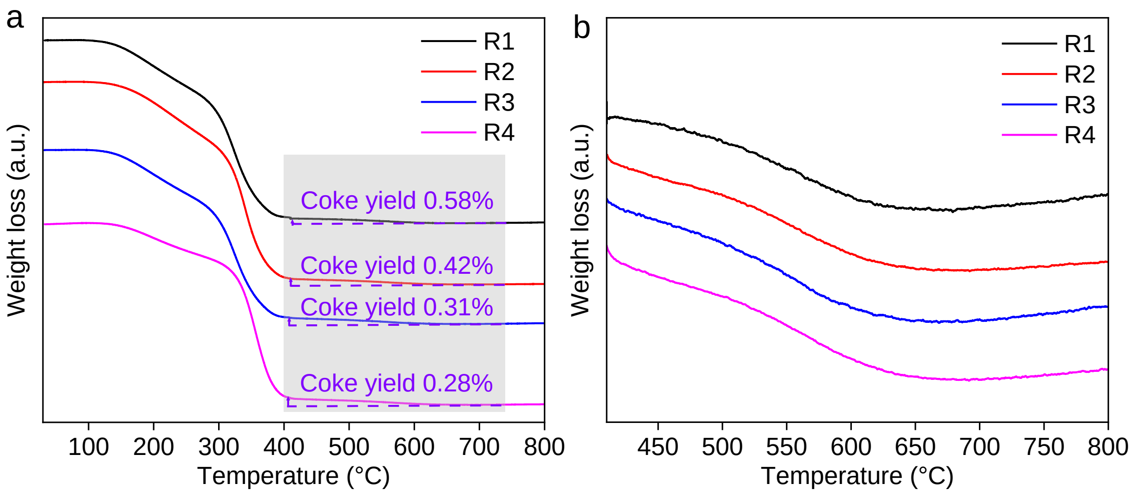

Supplementary Fig. 15 | Full (a) and enlarged (b) TGA plots of dried reaction solid residues in 4 reaction cycle. The weight loss below 400 ℃ is assigned to unconverted oligomers. The weight loss above 400 ℃ is assigned to coke. Coke yield = [Coke] / $[HDPE]_0$ × 100%, [Coke] denotes the mass of coke in reaction residues, $[Polyolefin]_0$ denotes the mass of polyolefin before reaction.

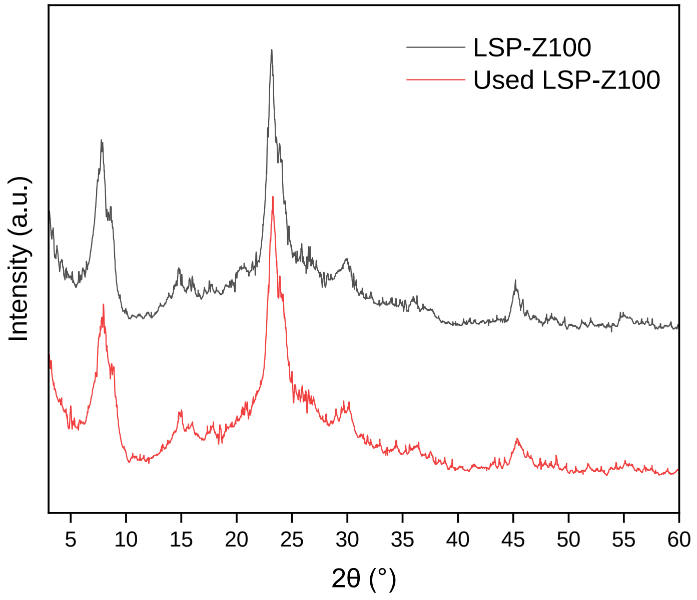

Supplementary Fig. 16 | XRD patterns of LSP-Z100 before and after 5 recycle runs.

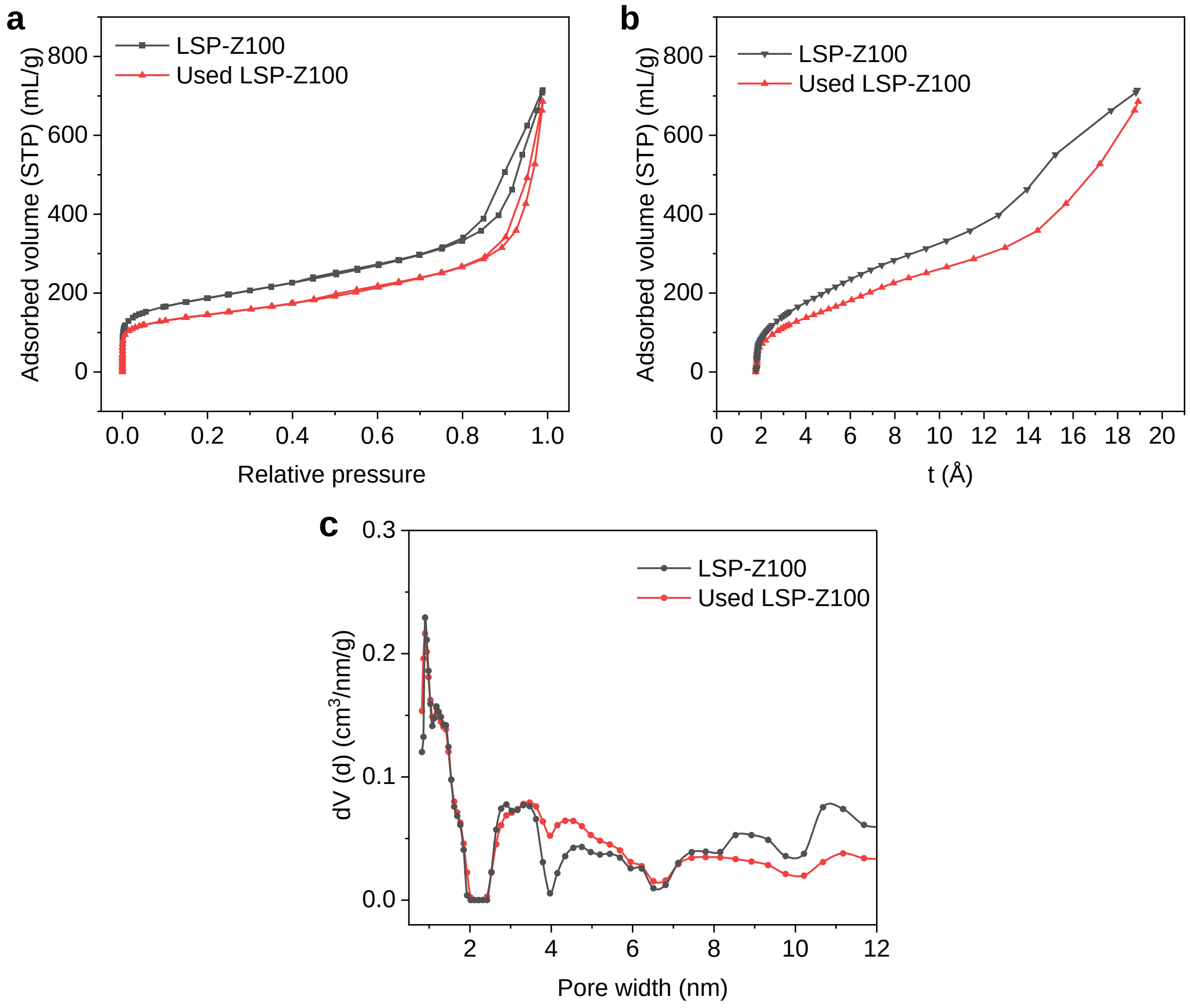

Supplementary Fig. 17 | N_2 adsorption isotherms of LSP-Z100 before and after 5 recycle runs. (**a**) N_2 adsorption/desorption isotherms. (**b**) t-plot profile. (**c**) Non-local DFT pore size distributions.

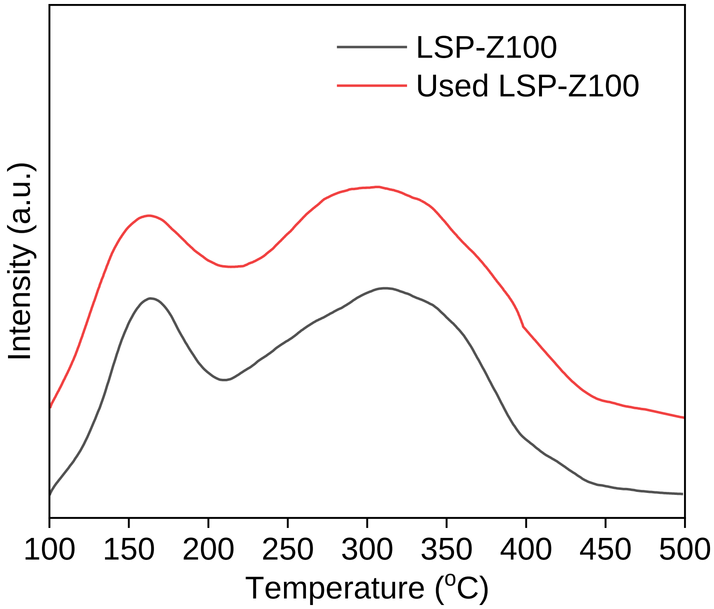

Supplementary Fig. 18 | NH_3-TPD profiles of LSP-Z100 before and after 5 recycle runs.

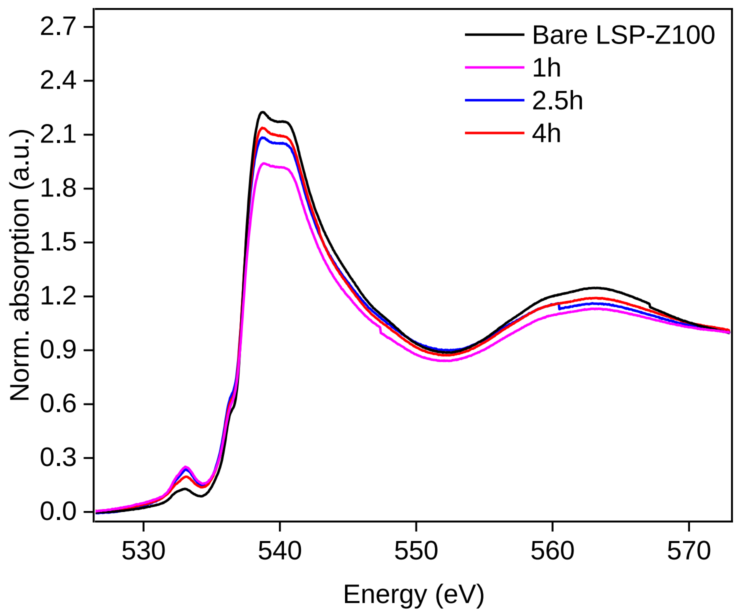

Supplementary Fig. 19 | O *K*-edge NEXAFS spectra for LSP-Z100 before reaction and LSP-Z100 during the reaction. In a typical sample preparation, 0.09 g of LSP zeolite and 0.45 g of HDPE were mixed and heated at 240 °C for 1 h, 2.5 h and 4 h under 0.1 MPa N_2.

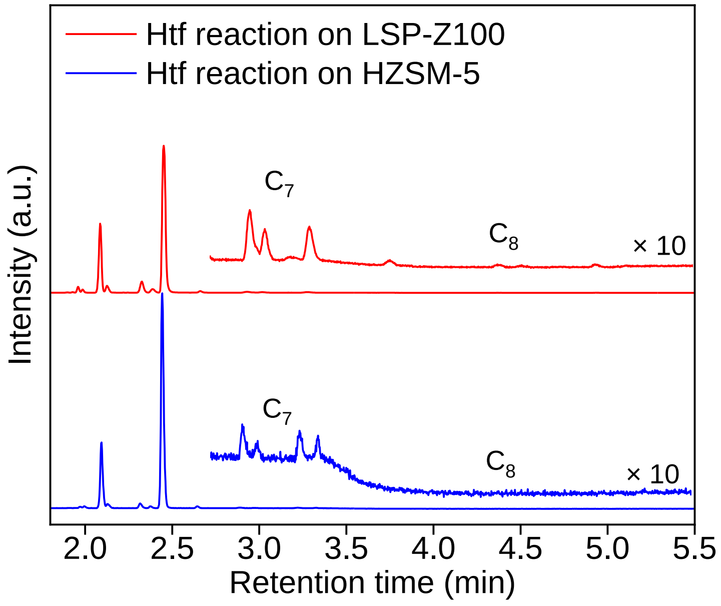

Supplementary Fig. 20 | GC traces of i-C_5H_{12}/C_6D_{14} hydride transfer reactions catalysed by LSP-Z100 and HZSM-5. Reaction conditions: catalyst, 0.075 g; 2-methylbutane, 0.075 g; n-hexane (d-14), 0.15 g; temperature, 240 ℃; reaction time, 1 hour; N_2 atmosphere, 0.1 MPa.

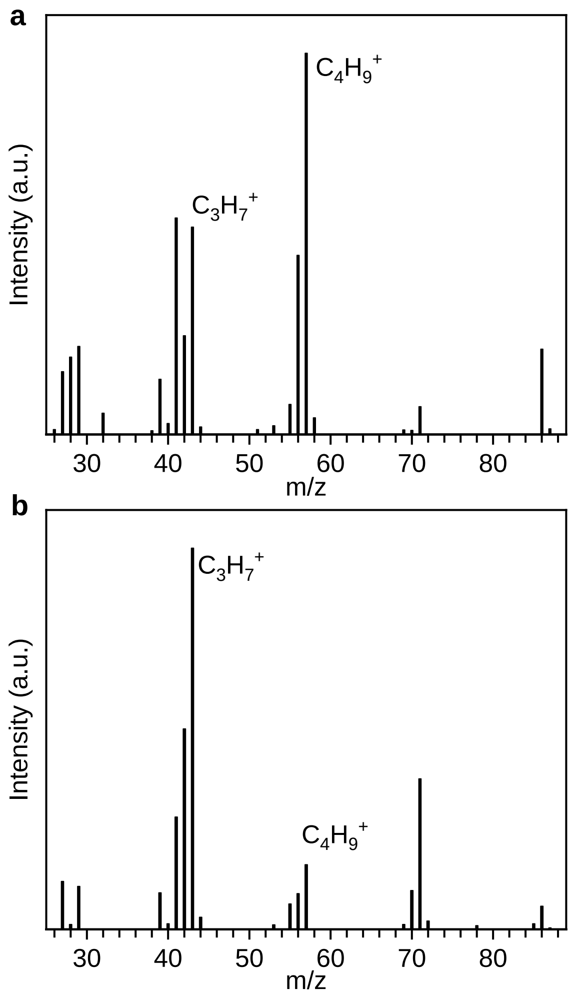

Supplementary Fig. 21 | Comparison of mass spectra of n-hexane (a) and 2-methylbutane (b). The base peak in mass spectrum of 2-methylbutane at m/z=43 ($C_3H_7^+$) demonstrates the isomerized structure of 2-methylbutane.

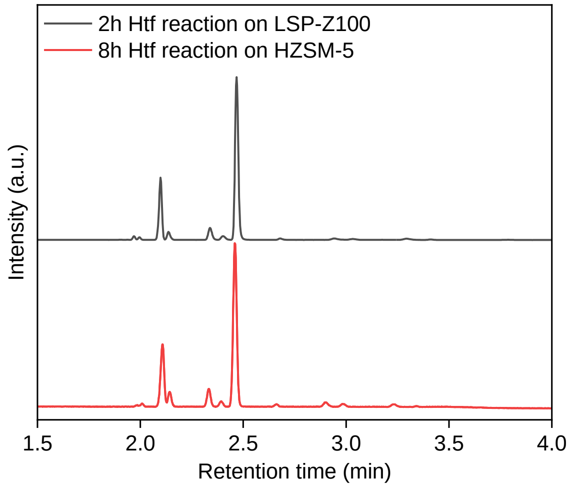

Supplementary Fig. 22 | GC traces of i-C_5H_{12}/C_6D_{14} hydride transfer reactions catalysed by LSP-Z100 for 2 hours and by HZSM-5 for 8 hours. Reaction conditions: catalyst, 0.075 g; 2-methylbutane, 0.075 g; n-hexane (d-14), 0.15 g; temperature, 240 ℃; N_2 atmosphere, 0.1 MPa.

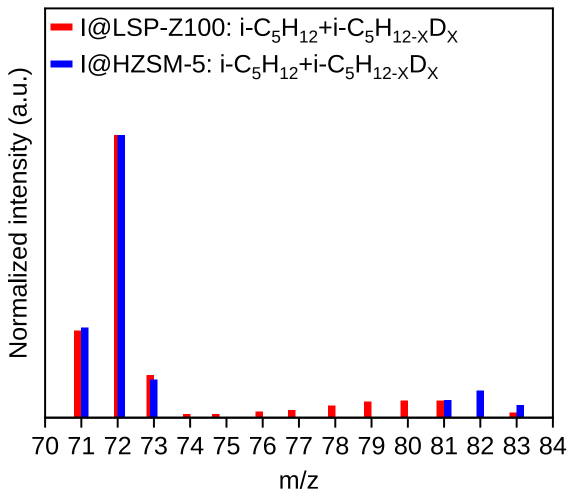

Supplementary Fig. 23 | Mass spectra of 2-methylbutane after reaction on HZSM-5 for 8 hours and on LSP-Z100 for 2 hours. Reaction conditions: catalyst, 0.075 g; 2-methylbutane, 0.075 g; n-hexane (d-14), 0.15 g; temperature, 240 ℃; N_2 atmosphere, 0.1 MPa.

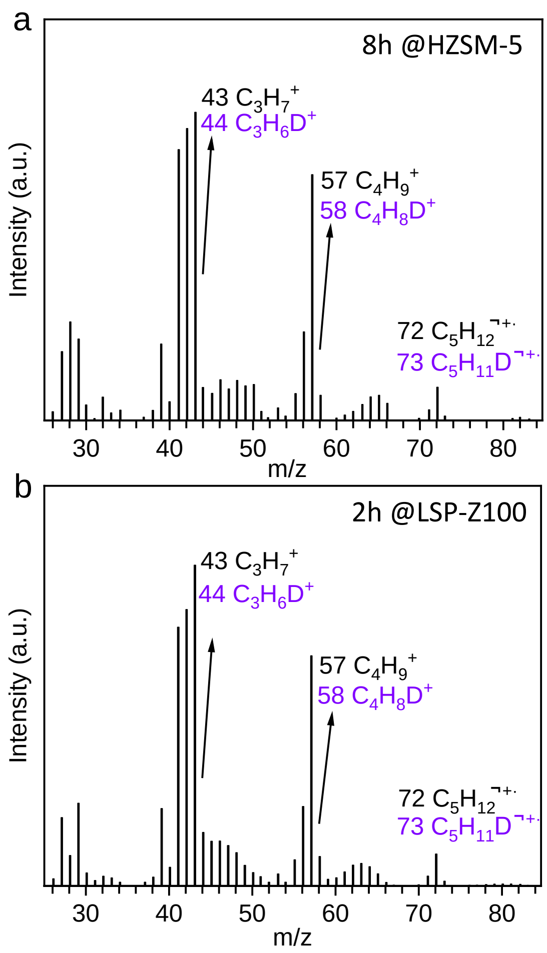

Supplementary Fig. 24 | Mass spectra of 2-methylbutane after reaction on HZSM-5 for 8 hours (a) and on LSP-Z100 for 2 hours (b). Reaction conditions: catalyst, 0.075 g; 2-methylbutane, 0.075 g; n-hexane (d-14), 0.15 g; temperature, 240 ℃; N_2 atmosphere, 0.1 MPa.

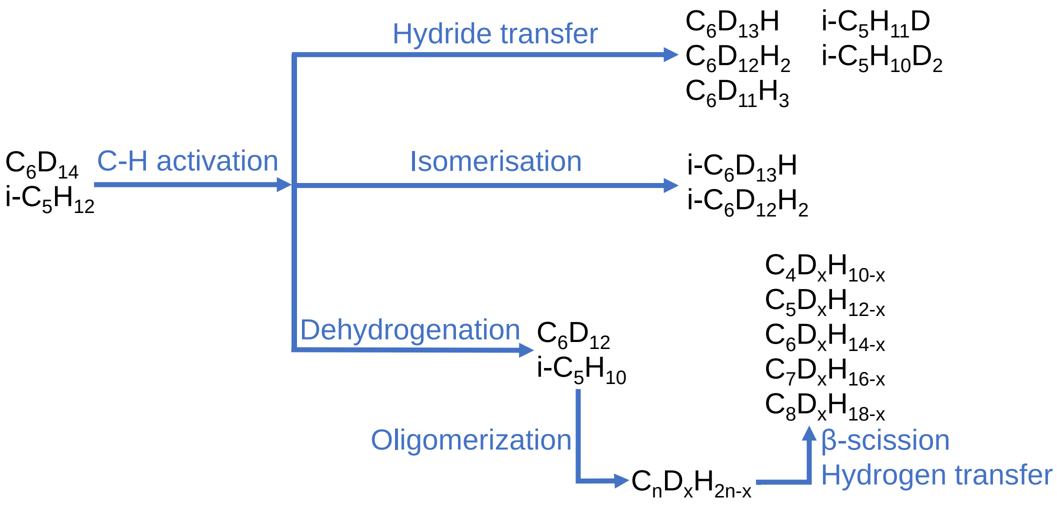

Supplementary Fig. 25 | Scheme of i-C_5H_{12}/C_6D_{14} hydride transfer, isomerisation, dehydrogenation/oligomerisation/β-scission/hydrogen transfer reactions.

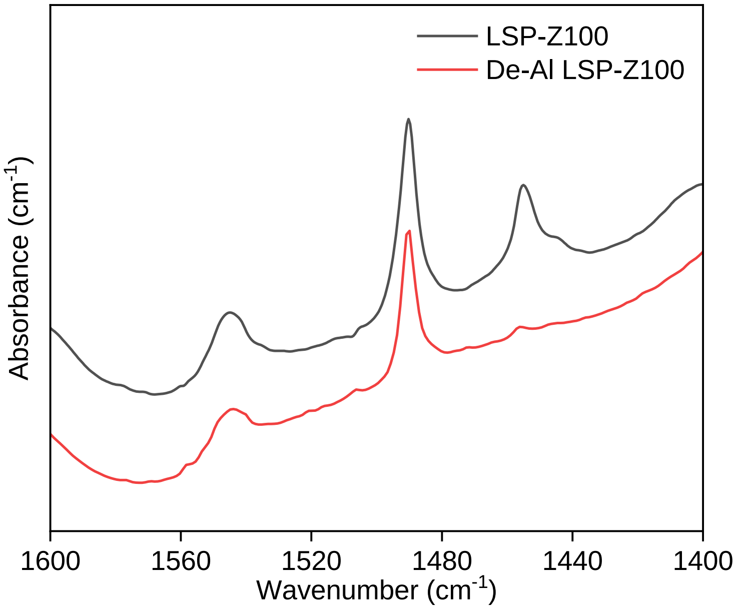

Supplementary Fig. 26 | IR spectra before and after adsorption of pyridine at variable temperatures on dealuminated LSP-Z100 (De-Al LSP-Z100).

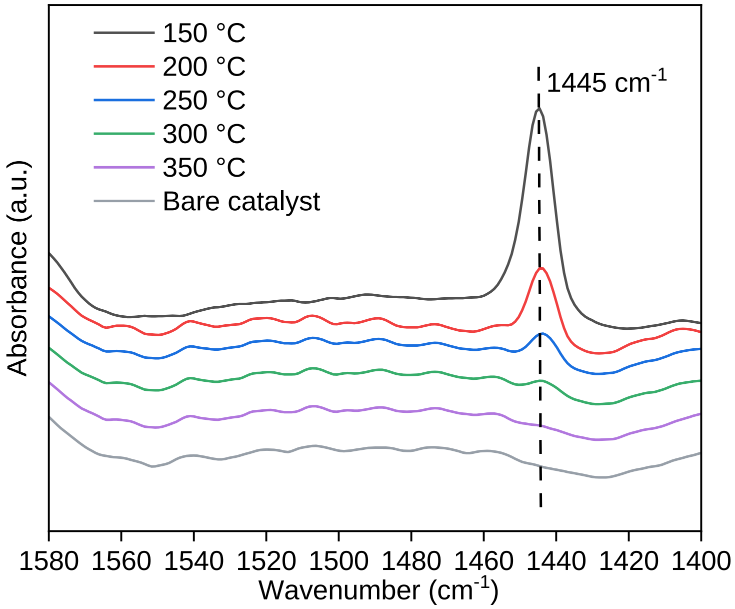

Supplementary Fig. 27 | IR spectra before and after adsorption of pyridine at variable temperatures on pure silicate LSP (Si-LSP).

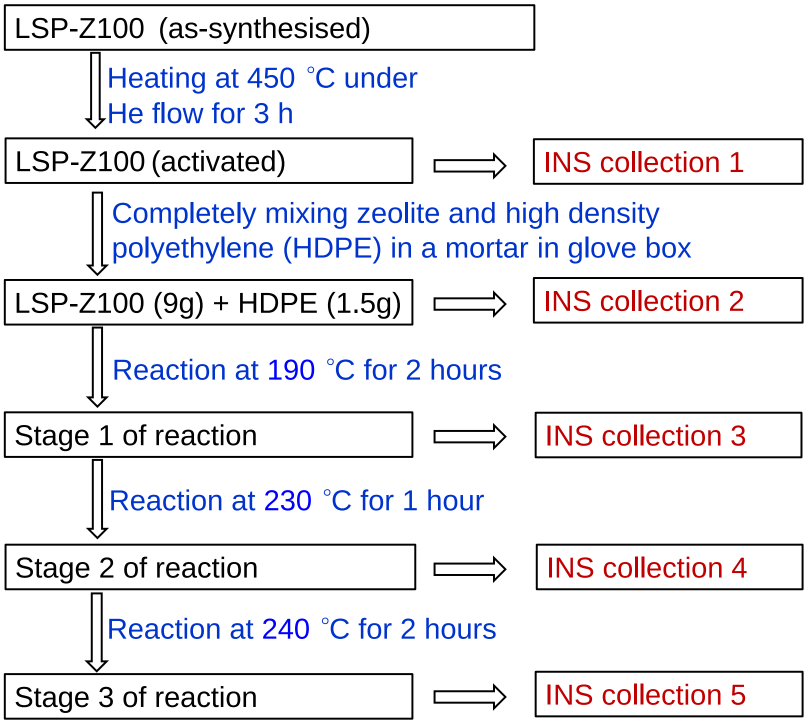

Supplementary Fig. 28 | Schematic view of the procedure of *operando* INS experiment and data collection.

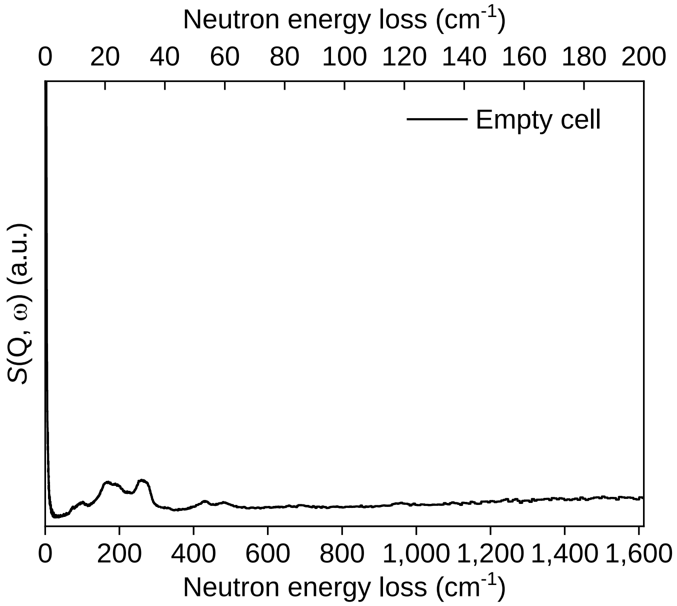

Supplementary Fig. 29 | View of the INS spectrum for the empty catalysis cell. The empty cell has the features below 300 cm^{-1}.

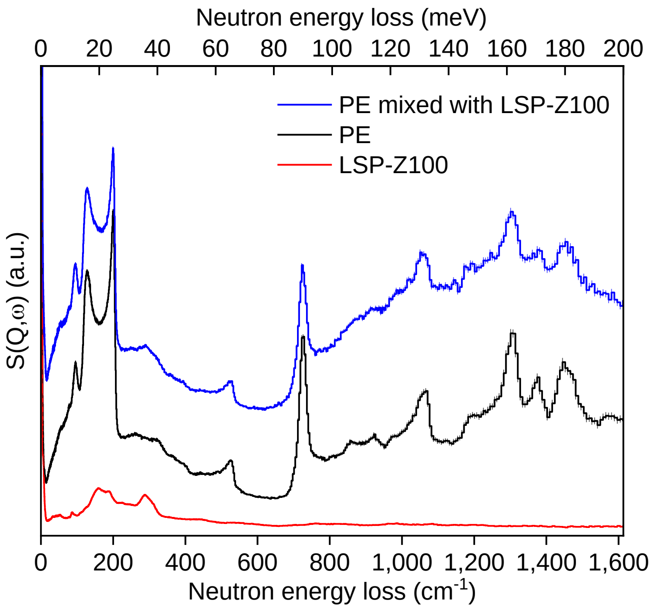

Supplementary Fig. 30 | Comparison of INS spectra for LSP-Z100, solid HDPE and mixture of HDPE and LSP-Z100. The bare catalyst, LSP-Z100, gives no prominent features in the measured INS spectrum at 0-1600 cm^{-1} as a result of the low cross sections of Si, Al and O for neutron scattering. The features below 300 cm^{-1} are contributed by the catalysis cell (Supplementary Fig. 29).

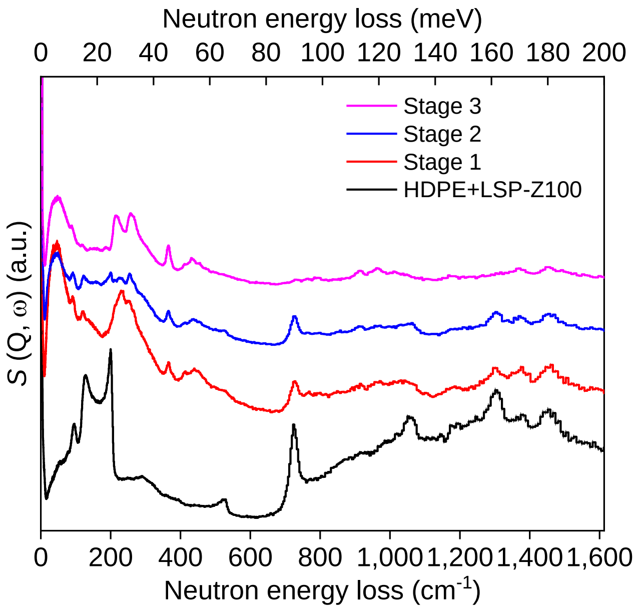

Supplementary Fig. 31 | Comparison of INS spectra for solid HDPE and reacted HDPE over LSP-Z100. These profiles are not subtracted of the LSP-Z100.

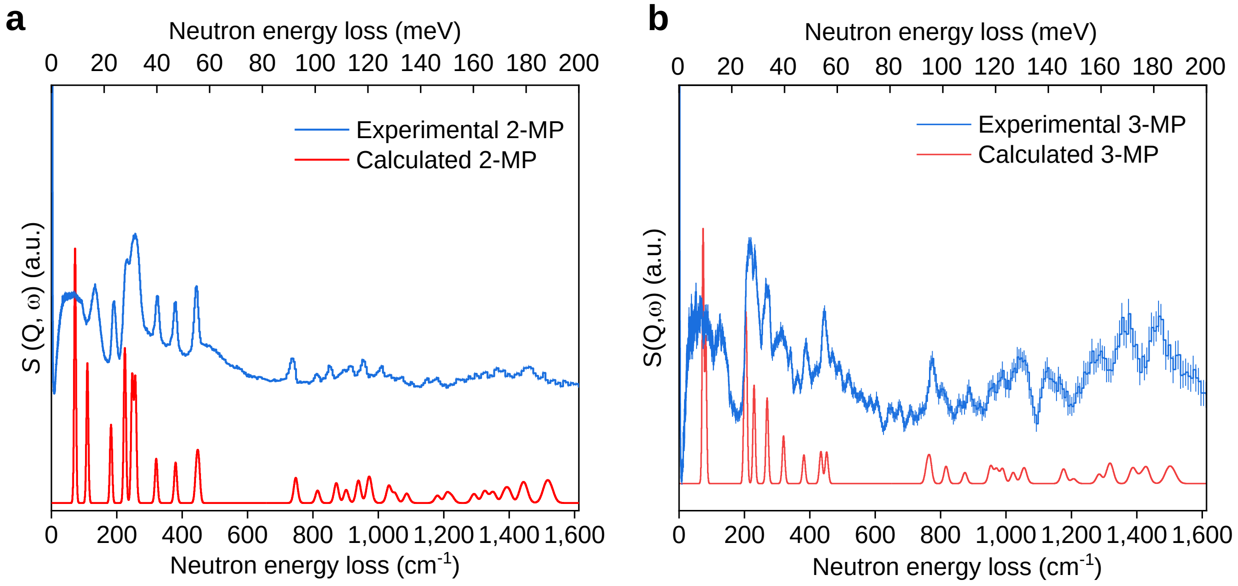

Supplementary Fig. 32 | Comparison of INS spectra of experimental solid 2-methylpentane (2-MP) (**a**) and 3-methylpentane (3-MP) (**b**) with those of calculated single molecule. Abscissa is scaled down by 0.94 for 2-MP and 0.93 for 3-MP.

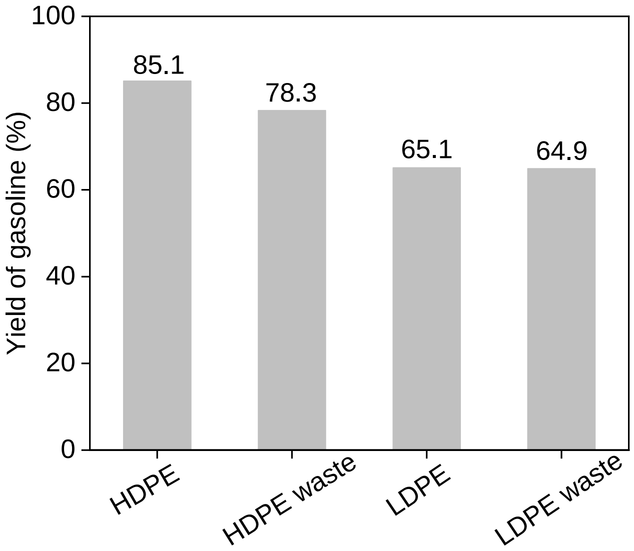

Supplementary Fig. 33 | Yields of gasoline of the catalytic conversion of reagent PE and waste PE. Reaction conditions: LSP-Z100, 0.09 g; plastics, 0.45 g; temperature, 260 ℃; reaction time, 4 hours; N_2 atmosphere, 0.1 MPa.

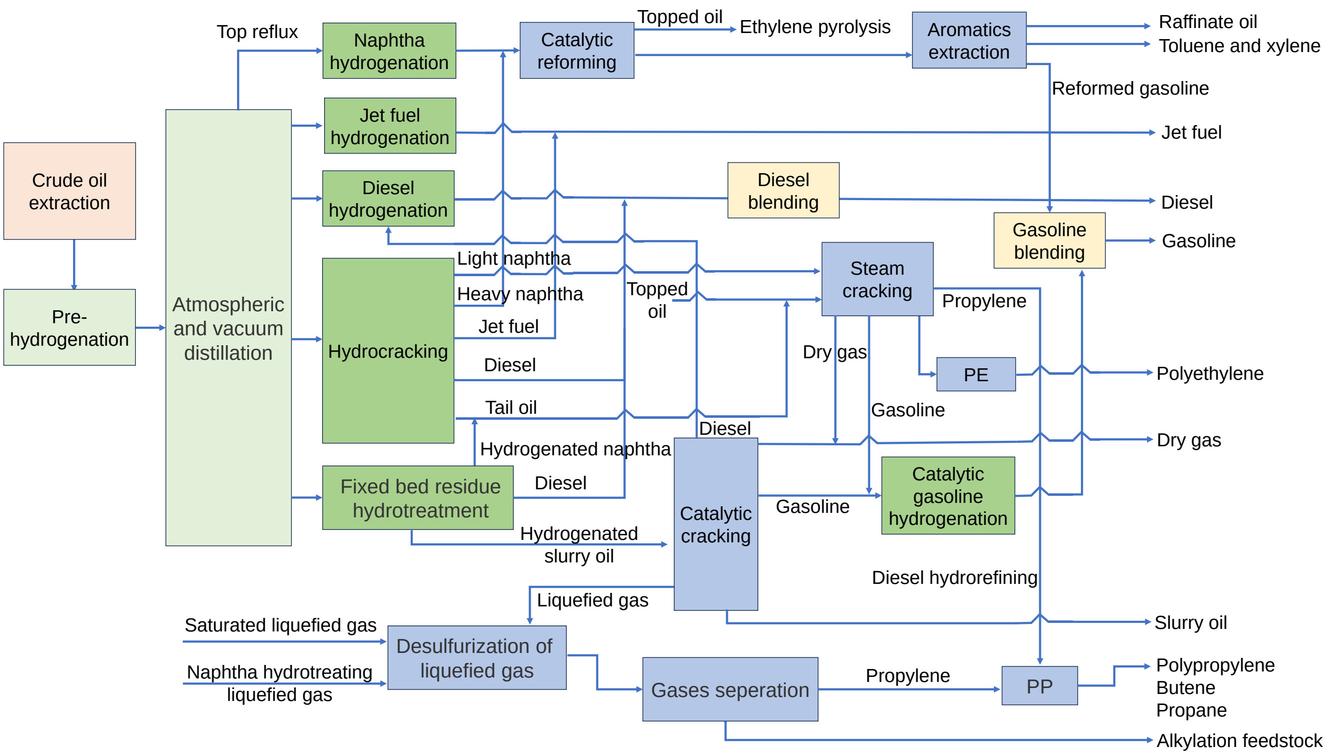

Supplementary Fig. 34 | Process flowsheet to produce fuels and petrochemicals from crude oil in the integrated refining plant with 10000 kt/a processing intake.

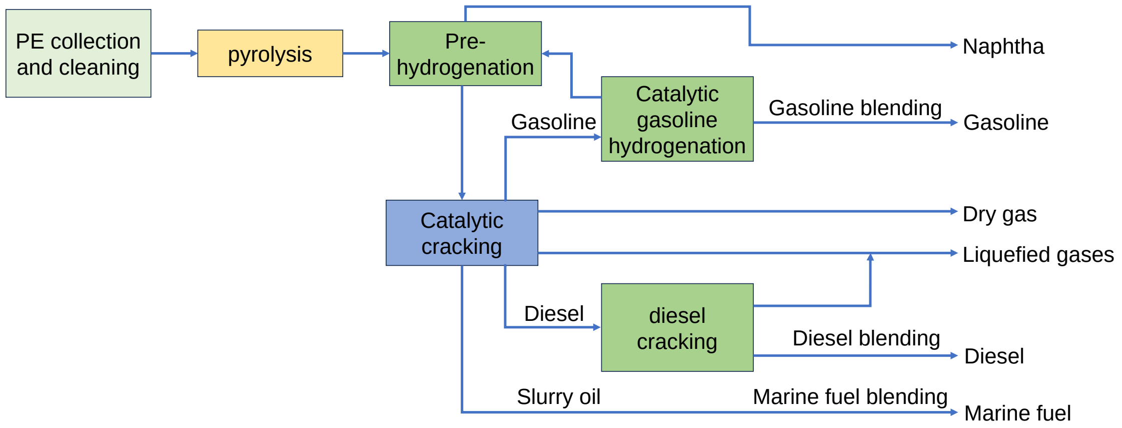

Supplementary Fig. 35 | Process flowsheet to produce fuels from PE via pyrolysis in the plastic pyrolysis demonstration plant with 100 kt/a processing intake.

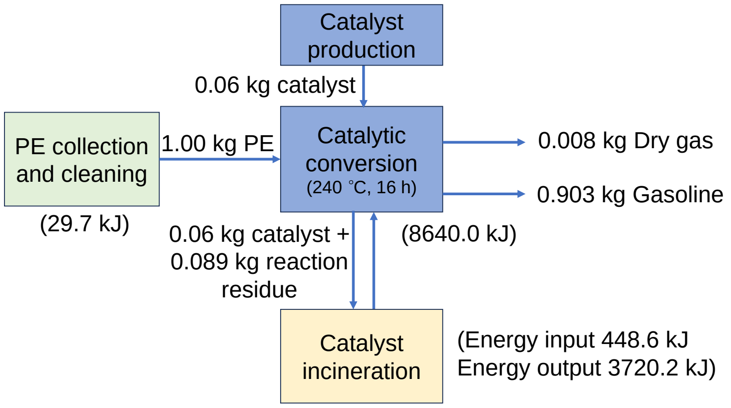

Supplementary Fig. 36 | Process flowsheet to produce fuels from PE via catalytic conversion. The energy cost estimation of catalytic cracking section is based on the results of 4 consecutive cycles, so the catalytic conversion section is fed with PE every 4 hours and continue for 16 hours. Heats of combustion in catalyst incineration section is used as energy supply to the system.

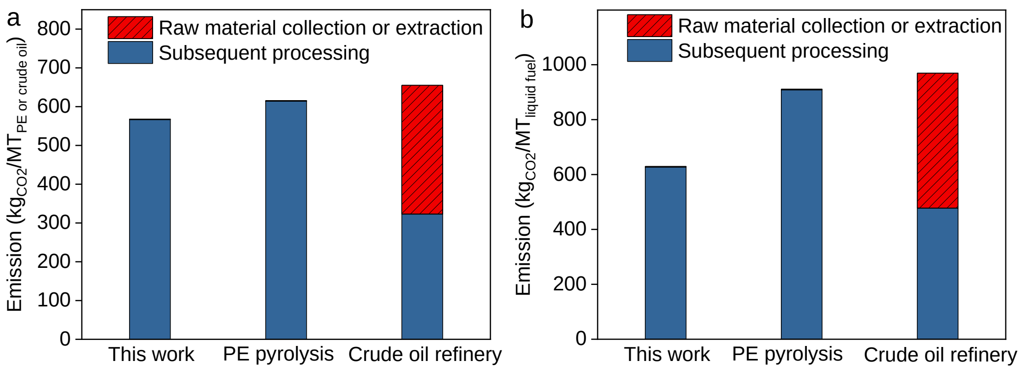

Supplementary Fig. 37 | CO_2 emission of liquid fuel production using strategy of this work, PE pyrolysis and crude oil refinery when converting 1 MT PE or crude oil (a) and when producing 1 MT liquid fuel (b). The CO_2 emissions of plastic pyrolysis and petroleum refinery are calculated based on the data of the plastic pyrolysis demonstration plant with 100 kt/a processing intake and the integrated refining plant with 10000 kt/a processing intake, respectively, of SINOPEC Research Institute of Petroleum Processing Co., LTD.

Supplementary Table 1. Comparison of the catalytic performance of reported strategies for the conversion of polyolefins. Cat/sub is the mass ratio of catalyst and substrate; N.A. is not applicable.

Strategy	Substrate	Catalyst	Cat./sub.	Reaction conditions	Products and yield	Ref.
Partial dehydrogenation and isomerizing ethenolysis	PE	Iridium-pincer complex /metathesis catalyst/[PdP(^{t}Bu)$_3$(μ-Br)]$_2$	1/1.25	130-200 °C, 16 h, 25 bar C_2H_4	C_3H_6, 80%	Ref.[23]
Electrified spatiotemporal heating	PP	No catalyst	NA	600 °C in every pulsed heating	Propylene, 36%	Ref.[24]
Pairing chemical oxidation and biological funneling	PE	Acetate salts of Co and Mn, N-hydroxyphthalimide, Pseudomonas putida	~1/3	160-210 °C, 8 bar O_2, 2-5 h	Polyhydroxy-alkanoates or β-ketoadipate, 57-69%	Ref.[25]
	PE	Ru/C	1/28	200°C, 20 bar H_2,16 h	C_8-C_{45}, 45%	Ref.[26]
	LDPE	Ru-WZr	1/40	250□, 50 bar H_2, 2h.	C_4-C_{12}, 19%	Ref.[27]
Hydrogenolysis	PP	Ru/TiO_2	1/20	250°C, 30 bar H_2, 16h	Oil with weight-average molecular weight of 0.9 kDa, 65.6%	Ref.[8]
	PE, PP	Ru/CeO_2	NA	260°C, 30 bar H_2, 18h	C_6-C_{40}, 70%	Ref.[10]
	HDPE	Mesoporous SiO_2/Pt/SiO_2	1/212	250°C, 13.8 bar H_2, 5.5 days	C_9-C_{15}, 31%	Ref.[28]
Hydrocracking	LDPE	Pt/WO_3/ZrO_2+HY	1/10	250°C, 30 bar H_2, 2h	C_4-C_{12}, 73%	Ref.[3]
Cross alkene metathesis	PE	Iridium-pincer complex/Re_2O_7/γ-Al_2O_3	NA	150 °C, 72 h, excess C_6H_{14}	C_3-C_{12}, 81.8%	Ref.[29]
Tandem cracking-alkylation	PE	[C_4Py]Cl-$AlCl_3$	NA	70 °C, 4 h, excess i-C_5H_{12}	C_6-C_{12}, 70%	Ref.[2]
Hydrogenolysis /aromatisation	PE	Pt/γ-Al_2O_3	2/1.18	280°C, 24 h	Long-chain alkylaromatics, 80%	Ref.[30]
	LDPE	Al-SBA-15	1/3	250 to 700 °C	Not reported	Ref.[31]
Thermal/catalytic cracking	PP	Spent FCC catalyst	1/2	450°C, 45 min	C_4-C_{12}, 80% (with 15% olefin)	Ref.[32]
	PE	ZSM-5 nanosheets	1/5	280 °C, 7h	C_1-C_7, 74.6% (with >50% olefin)	Ref.[1]
Self-supplied hydrogen strategy	HDPE	LSP-Z100	1/5	240°C, 4h	C_4-C_{12} gasoline, 81.2%	this work
				240°C, 24.5h	C_4-C_{12} gasoline, 87.2%	

Supplementary Table 2. Textural properties determined from nitrogen sorption at 77K.

Samples	A_{BET} $(m^2 \cdot g^{-1})$[a]	A_{mic} $(m^2 \cdot g^{-1})$[b]	A_{ext} $(m^2 \cdot g^{-1})$[c]	V_{mic} $(cm^3 \cdot g^{-1})$[d]	V_{total} $(cm^3 \cdot g^{-1})$[e]	D (nm)[f]
LSP-Z100	670	218	452	0.09	1.11	6.60
LSP-Z75	572	219	353	0.09	1.20	8.36
HZSM-5	413	344	69	0.14	0.20	2.40

[a]BET specific surface area;

[b]Micropore surface area;

[c]External surface area;

[d]Micropore volume

[e]Total pore volume

[f]Average pore diameter

Supplementary Table 3. Summary of different types of Si atoms from the ^{29}Si NMR data.

^{29}Si Chemical Shift (ppm)	Assignment
-111 to -119	$Si(OSi)_4$
-105 to -108	$Si(OSi)_3OAl$
-104 to -101	$Si(OSi)_3(OH)$
-93 to -92	$Si(OSi)_2(OH)_2$

Supplementary Table 4. Elemental analysis of zeolites by Inductively Coupled Plasma Mass Spectrometry (ICP-MS).

Samples	Si (*wt*. %)	RSD[a] of Si content (%)	Al (*wt*. %)	RSD[a] of Al content (%)	Si/Al
LSP-Z75	45.73	1.17	0.90	1.15	49
LSP-Z100	45.86	0.79	0.78	0.85	57
HZSM-5	45.90	0.89	0.74	0.77	60

[a] RSD: relative standard deviation

Supplementary Table 5. Summary of acidities of LSP-Z100 and HZSM-5. Data were determined by NH_3-TPD and Pyridine-IR studies.

Catalyst	Weak acid[a] (mmol·g^{-1})	Strong acid[a] (mmol·g^{-1})	Total acid[a] (mmol·g^{-1})	L/B acid ratio[b]
LSP-Z100	0.050	0.141	0.191	1.049
HZSM-5	0.065	0.125	0.190	0.366

[a]From NH_3-TPD

[b]From Pyridine-IR

Supplementary Table 6. Summary of the HDPE conversion and product yields over various catalysts[a].

Catalysts	Conversion (%)				RSD[b] of conversion (%)	Yield (%)				RSD[b] of gasoline yield (%)
	1	2	3	average		1	2	3	average	
HZSM-5	32.2	35.7	37.5	35.1	7.7	31.6	35.0	36.8	34.5	7.7
HY	3.8	3.3	3.6	3.6	6.5	3.7	3.3	3.6	3.5	5.3
USY	9.1	7.9	8.9	8.6	7.4	9.0	7.8	8.8	8.5	7.8
Meso-HY	19.0	20.2	19.8	19.7	3.1	18.7	19.9	19.3	19.3	3.1
MCM-41	9.6	8.4	8.9	9.0	6.7	9.3	8.1	8.7	8.7	6.9
SBA-15	<1%	<1%	<1%	-	-	<1%	<1%	<1%	-	-
LSP-Z75	78.8	76.9	79.3	78.3	1.6	77.2	76.0	76.2	76.5	0.8
LSP-Z100	81.7	82.0	81.8	81.8	0.2	81.3	81.4	81.0	81.2	0.3
LSP-Z100[c]	90.3	90.4	89.8	90.2	0.4	87.2	87.6	86.7	87.2	0.5
Fresh FCC catalyst	5.1	6.0	6.2	5.8	10.2	4.9	5.8	6.0	5.6	10.5
Spent FCC catalyst	<1%	<1%	<1%	-	-	<1%	<1%	<1%	-	-
Short b-axis ZSM-5	11.8	13.4	10.9	12.0	10.5	11.6	13.1	10.6	11.8	10.7
$[C_4Py]Cl-AlCl_3$	<1%	<1%	<1%	-	-	<1%	<1%	<1%	-	-
Ru/C	<1%	<1%	<1%	-	-	<1%	<1%	<1%	-	-

Pt/γ-Al_2O_3	<1%	<1%	<1%	-	-	<1%	<1%	<1%	-	-
Pt/WO_3/ZrO_2+HY(30)	12.0	11.8	13.6	12.5	7.9	11.8	11.4	13.3	12.2	8.2

[a]Reaction conditions, catalyst, 0.09 g; HDPE, 0.45 g; temperature, 240 ℃; reaction time, 4 h; N_2 atmosphere, 0.1 MPa. [b]The RSD (Relative standard deviation) value is calculation by equation: $RSD = \frac{s}{\bar{x}}\,;\ s = \sqrt{\frac{\sum_{i=1}^{n}(x_i-\bar{x})^2}{n-1}}$. [c]Reaction time is 24.5 h.

Supplementary Table 7. Product composition of HDPE conversion catalyzed by LSP-Z100. Reaction conditions: catalyst, 0.09 g; HDPE powder, 0.45 g; Reaction temperature, 240 ℃; Reaction time: 4 hours; Nitrogen, 0.1 MPa.

Composition	Selectivity (%)	Composition	Selectivity (%)
hydrogen	0.01	2-methyl-1butene	0.05
methane, ethane, ethene, propane	0.77	2-methyl-2-butene	0.11
**Alkane**		2-methyl-2-butene	3-hexene
butane	1.70	3-methyl-2-pentane	0.17
2-methylpropane	6.27	2-ethyl-1-butene	0.03
pentane	3.69	2-methyl-1-pentene	0.01
2-methylbutane	12.0	4-methyl-2-hexene	0.03
hexane	3.39	2-methyl-2-hexene	0.11
2,3-dimethylbutane	1.00	3-methyl-2-hexene	0.04
2-methylpentane	11.2	2-methyl-3-hexene	0.06
3-methylpentane	3.46	4,4-dimethyl-2-pentene	0.10
heptane	1.86	2-propyl-1-pentene	0.14
2-methylhexane	8.01	3,4-dimethyl-3-hexene	0.10
3-methylhexane	4.76	2,3-dimethyl-1-hexene	0.10
2,4-dimethylpentane	0.96	**Cycloalkane**	
2,3-dimethylpentane	0.86	methylcyclohexane	0.56
octane	0.99	1,3-dimethylcyclopentane	0.43
2-methylheptane	3.23	Ethyl-cyclopentane	0.18
3-methylheptane	4.25	1,3-dimethylcyclohexane	0.61
2,4-dimethylhexane	0.72	1,4-dimethylcyclohexane	1.04
3,4-dimethylhexane	1.26	1-methyl-2-ethylcyclopentane	0.31
2-methyl-3-ethylpentane	0.43	1,2,5-trimethylcyclohexane	0.44
2,3,3-trimethylpentane	0.17	1,2,3-trimethylcyclohexane	0.30
2,3,4-trimethylpentane	0.96	1-methyl-4-ethylcyclohexane	0.07
3-methyloctane	1.91	1-methyl-3-ethylcyclohexane	0.40
2,5-dimethylheptane	3.12	**Aromatics**	
2,2-dimethylheptane	0.40	toluene	0.20
3,5-dimethylheptane	1.12	1,2-xylene	0.22
2,4-dimethyl-3-ethylpentane	0.42	1,3-xylene	1.00
3-methylnonane	0.70	1,4-xylene	0.17
2,4-dimethyl-3-isopropylpentane	0.46	1-ethyl-2-methylbenzene	0.56
3,3-dimethyl-4-ethylhexane	1.16	1-ethyl-3-methylbenzene	1.29
3,4,5-trimethylheptane	0.74	1,2,3-trimethylbenzene	0.50
2,5-dimethylnonane	0.33	1,2,5trimethylbenzene	0.73
2,6-dimethylnonane	0.38	1,3,5-trimethylbenzene	0.98
2,4-dimethylnonane	0.55	1,2,4,5-tetramethylbenzene	0.71
2,4-dimethyl-3-isopropylhexane	0.05	3-methyl-1-isopropylbenzene	0.80
4-methyl-3-ethylnonane	0.44	1,4-dimethyl-2-ethylbenzene	0.32
2-methyl-5-ethylnonane	0.36	1,3-diethyl-5-methylbenzene	0.27
2,4-dimethyl-3-isopropylheptane	0.21	4-sec-butyltoluene	0.38
2,6-dimethyldecane	0.05	1,2,3,4,5-pentamethylbenzene	0.32
**Alkene**		1,3,5-trimethyl-2-poropylbenzene	0.42
1-butene	0.09	1,3,5-trimethyl-2-isopropylbenzene	0.54
2-butene	0.09	1,3,5-triethylbenzene	0.56

Supplementary Table 8. Summary of the HDPE conversion and products selectivity over various zeolites[a].

Entry	Catalysts	Conversion (%)	Selectivity (%)			Components of gasoline range products (%)				
			C_1-C_3	C_4-C_{12} (gasoline range)	$>C_{12}$[b]	n-alkanes	i-alkanes	alkene	cycloalkanes	aromatics
1	HZSM-5	35.1	1.7	98.3	0.0	16.0	43.0	27.8	5.7	7.5
2	HY[c]	3.6	2.8	97.2	0.0	3.3	78.3	3.9	4.1	10.4
3	USY	8.6	1.2	98.8	0.0	3.8	77.2	1.0	6.1	11.9
4	Meso-HY	19.7	2.0	98.0	0.0	5.2	79.5	4.7	5.3	5.3
5	MCM-41	9.0	3.3	96.7	0.0	8.1	78.5	5.2	3.1	5.1
6	SBA-15	<1	-	-	-	-	-	-	-	-
7	LSP-Z75	78.3	2.3	97.7	0.0	10.1	71.7	3.2	4.5	10.5
8	LSP-Z100	81.8	0.7	99.3	0.0	11.7	72.5	1.4	4.3	10.1
9	LSP-Z100[c]	90.2	3.3	96.7	0.0	14.6	68.7	0.8	4.2	11.7

[a]Reaction conditions, catalyst, 0.09 g; HDPE, 0.45 g; temperature, 240 ℃; reaction time, 4 h; N_2 atmosphere, 0.1 MPa. [b]C_{12+} compounds in liquid products were not detected by GC. [c]Reaction time is 24.5 h.

Supplementary Table 9. Prediction of gasoline Research Octane Number (RON) based on composition. The prediction model is obtained from ref.[33]

	Weight fraction (%) and predicted ON	
	Gasoline from HDPE	Commercial gasoline
NP[a]	11.7	9.8
NP1[b]	5.4	3.8
NP2[c]	6.3	6.0
IP[d]	73.8	51.5
IP1[e]	18.8	5.4
IP2[f]	55.1	46.1
CP[g]	4.4	6.2
Ar[h]	10.1	32.5
Research Octane Number[i]	88.0	86.6

[a]Total n-paraffins

[b]n-butane and n-pentane

[c]n-paraffins except n-butane and n-pentane

[d]Total iso-paraffins

[e]iso-butane and iso-pentane and butene

[f]iso-paraffins and olefins except iso-butane, iso-pentane and butene

[g]Total cycloparaffins

[h]Total aromatics and additive ethanol

[i]Prediction equation:

$$ON = -1.0729\,NP2 + 0.7875IP1 + 0.0976IP2 + 0.3395CP + 0.4049Ar + 69.0306$$

Supplementary Table 10. Results of the HDPE conversion and gasoline yield over various zeolites.

Entry	Catalyst	Conversion (%)	Yield (%)			Components of gasoline range products (%)				
			C_1-C_3	C_4-C_{12} (gasoline range)	>C_{12}[b]	n-alkanes	i-alkanes	alkenes	Cyclo-alkanes	aromatics
1	HZSM-5[a]	35.1	0.6	34.5	0.0	16.0	43.0	27.8	5.7	7.5
2	HZSM-5 micron[a]	16.2	0.4	15.8	0.0	16.4	30.0	45.8	2.9	4.9
3	DTBPy-LSP[a]	46.2	2.2	44.0	0.0	10.1	20.1	57.7	4.9	7.1
4	De-Al LSP[b]	58.9	0.4	58.5	0.0	11.1	66.5	9.1	3.3	10.0
5	Si-LSP[a]	0.0	-	-	-	-	-	-	-	-
6	LSP-Z100[c]	57.6	0.3	99.7	0.0	8.3	66.2	14.7	4.1	6.7
7	LSP-Z100 + Si-LSP[d]	67.6	0.7	99.3	0.0	10.9	65.6	13.1	2.9	7.5
8	LSP-Z100[a]	81.8	0.6	81.2	0.0	11.7	72.5	1.4	4.3	10.1
9	LSP-Z100 + Si-LSP[e]	83.1	0.7	82.4	0.0	10.1	70.4	1.8	2.3	15.4
10	HZSM-11[a]	12.0	0.5	11.5	0.0	14.2	27.8	49.0	4.2	4.6
11	HY (Si/Al=50)[a]	5.8	0.3	5.6	0.0	3.7	78.4	2.1	6.5	9.3

[a]Reaction conditions, catalyst, 0.09 g; HDPE, 0.45 g; temperature, 240 ℃; reaction time, 4.0 h; N_2 atmosphere, 0.1 MPa.

[b]Reaction conditions, catalyst, 0.1046 g (0.00167 mmol Lewis acid sites and 0.00837 mmol Brönsted acid sites calculated based on Supplementary Table 24; Similar amount of Brönsted acid sites but less amount of Lewis acid sites compared to entry 8); HDPE, 0.45 g; temperature, 240 ℃; reaction time, 4.0 h; N_2 atmosphere, 0.1 MPa.

[c]Reaction conditions, catalyst, 0.09 g; HDPE, 0.45 g; temperature, 240 ℃; reaction time, 1.5 h; N_2 atmosphere, 0.1 MPa.

[d]Reaction conditions, LSP-Z100, 0.09 g; Si-LSP, 0.03g; HDPE, 0.45 g; temperature, 240 ℃; reaction time, 1.5 h; N_2 atmosphere, 0.1 MPa.

[e]Reaction conditions, LSP-Z100, 0.09 g; Si-LSP, 0.03g; HDPE, 0.45 g; temperature, 240 ℃; reaction time, 4.0 h; N_2 atmosphere, 0.1 MPa.

Supplementary Table 11. Molecule models and predicted ^{13}C NMR chemical shift by an on-line NMR chemical shift prediction platform[13].

<table>
<tr><td rowspan="8">Branched chain oligomer</td><td rowspan="4">Middle chain branched oligomer</td><td>End chain carbon of straight chain (δ=14-15)</td><td>Carbon connected to end chain carbon (δ=24-26)</td></tr>
<tr><td>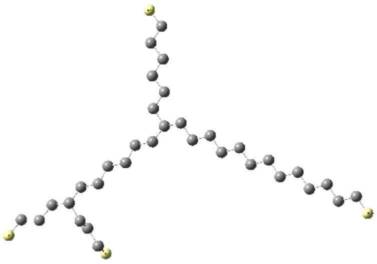</td><td>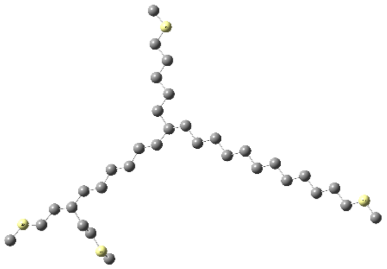</td></tr>
<tr><td>Methylene carbon (δ=32-34)</td><td>Tertiary carbon (δ=38-40)</td></tr>
<tr><td>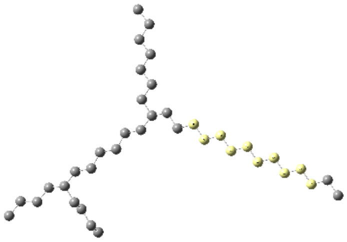</td><td>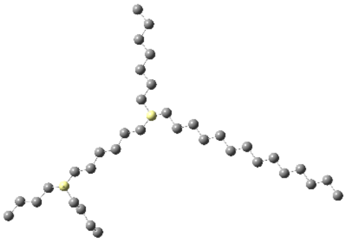</td></tr>
<tr><td rowspan="4">End chain branched oligomer</td><td>End chain carbon of branched chain (δ=20-22)</td><td>Tertiary carbon (δ=29-30)</td></tr>
<tr><td>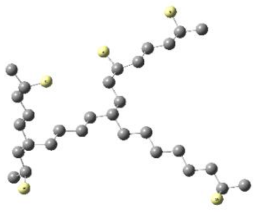</td><td>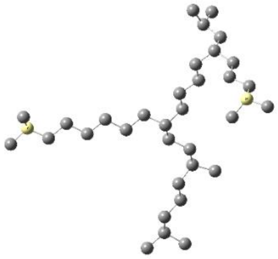</td></tr>
<tr><td>Tertiary carbon (δ=34-35)</td><td></td></tr>
<tr><td>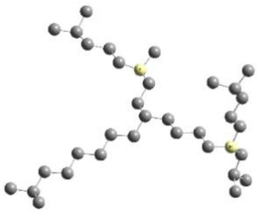</td><td></td></tr>
<tr><td colspan="2"></td><td>Carbon in aliphatic ring (δ=26-</td><td>Carbon in aliphatic ring</td></tr>
</table>

**Oligomer with aliphatic rings**	31)	(δ=40-45)
	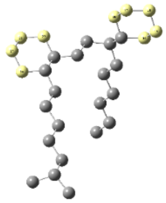	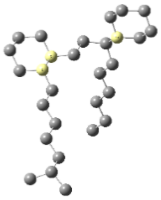
**Oligomer with aromatic rings and double bonds**	Carbon connected to aromatic rings (δ=45-55)	Carbon connected to double bond carbons (δ=29-31)
	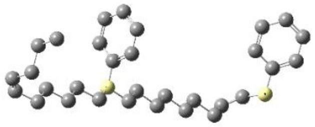	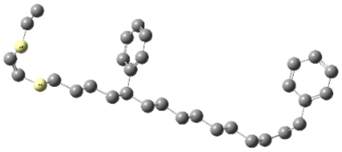
	Carbon in aromatic rings (δ=125-150)	Carbon in isolated double bonds (δ=125-150)
	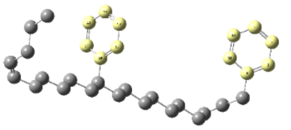	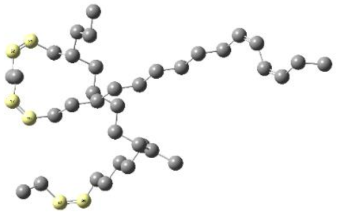
	Carbon in conjugated double bonds (δ=122-124)	
	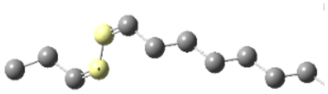	

Supplementary Table 12. Peak assignment of solid state ^{13}C NMR spectra of reaction solid residue.

Peak Shift (ppm)	Corresponding Carbon Atom
14-15	End chain carbon of straight chain
20-22	End chain carbon of branched chain
24-26	Carbon atom connected to end chain carbon
29-34	Carbon atom connected to double bond carbon
29-45	Tertiary carbon
32-34	Carbon of methylene in polymer
26-31, 40-45	Carbon in aliphatic ring
45-55	Carbon connected to aromatic ring
120-150	Carbon in aromatic ring
120-150	Double-bond carbon

Supplementary Table 13. Elemental analysis of HDPE powder, mixture of HDPE and LSP-Z100 and reaction solid residue in the time course study. It shows that the polymer in solid residue supplies hydrogen to products and the carbon and hydrogen are balanced in the closed system during all reactions.

	HDPE mixed with LSP-Z100	0.1h	1h	1.5h	2.5 h	4 h	7.5 h	24.5 h
C in residue (mol)		0.027	0.017	0.016	0.014	0.008	0.008	0.005
H in residue (mol)		0.053	0.032	0.030	0.025	0.014	0.013	0.008
H/C ratio of residue		1.963	1.882	1.875	1.786	1.750	1.625	1.600
C in GC-analyzed product (mol)		0.005	0.014	0.016	0.018	0.023	0.023	0.026
H in GC-analyzed product (mol)		0.011	0.031	0.034	0.040	0.051	0.052	0.057
H/C ratio of GC-analyzed product		2.200	2.214	2.125	2.222	2.217	2.261	2.192
All C in count (mol)	0.032	0.032	0.031	0.032	0.032	0.031	0.031	0.031
All H in count (mol)	0.065	0.064	0.063	0.064	0.065	0.065	0.065	0.065
Overall H/C ratio	2.031	2.000	2.032	2.000	2.031	2.097	2.097	2.097

Supplementary Table 14. Mass balance in the time course study.

Reaction time (h)	0.1	1	1.5	2.5	4	7.5	24.5
Mass of HDPE input (g)	0.450	0.450	0.450	0.450	0.450	0.450	0.450
Mass of products (g)	0.073	0.209	0.252	0.300	0.362	0.365	0.398
Mass of residues (g)	0.376	0.238	0.194	0.144	0.082	0.080	0.044
Mass balance (%)	0.222	0.667	0.889	1.333	1.333	1.111	1.778

Supplementary Table 15. Carbon rich products and unreacted PE proportion in solid residue.

	HDPE	0.1h	2.5 h	4 h	7.5 h	24.5 h
Mass weight percent of carbon rich products in solid residue (%)	-	12.3	26.5	40.7	45.5	46.9
C in carbon rich products (mol)		0.00338	0.00387	0.00287	0.00376	0.00242
H in carbon rich products (mol)		0.00577	0.00474	0.00374	0.00451	0.00284
H/C ratio of carbon rich products		1.704	1.224	1.303	1.201	1.174
C in unreacted PE (mol)		0.02362	0.01013	0.00513	0.00424	0.00258
H in unreacted PE (mol)		0.04723	0.02026	0.01026	0.00849	0.00516
Total C in solid residue (mol)	0.032	0.027	0.014	0.008	0.008	0.005
Total H solid residue (mol)	0.065	0.053	0.025	0.014	0.013	0.008
Overall H/C ratio of solid residue	2.031	1.963	1.786	1.750	1.625	1.600

Supplementary Table 16. Assignments for peaks in ^1H NMR spectrum of carbon rich products left in solid reaction residue.

Peak Shift (ppm)	Corresponding Carbon Atom
0.70-1.01	Methyl groups
1.01-1.43	Methylene groups
1.43-1.60	H in methyne groups
2.00-2.40	H in methyl group connected to aromatic rings
2.40-2.92	H in methylene group connected to aromatic rings
2.92-3.30	H in methyne group connected to aromatic rings
6.60-7.40	H in monoaromatic rings
7.40-8.00	H in polyaromatic rings

Supplementary Table 17. Assignments for various GD-ESI-MS series in the carbon rich products left in solid reaction residue.

Mass series	Assignments
14x+2	alkylnaphthalenes
14x+8	Alkylbenzenes
14x+1[a]	Oxidized alkylnaphthalenes
14x+3 [a]	Oxidized benzodicycloparaffins
14x+5 [a]	Oxidized benzocycloparaffins
14x+7 [a]	Oxidized alkylbenzenes
14x+9 [a]	Oxidized cycloparaffins with two aliphatic rings
14x+11 [a]	Oxidized cycloparaffins with an aliphatic ring
14x+13 [a]	Oxidized paraffins

[a] End chain methyl group -CH_3 was oxidized into $C=O^+$. *x* denotes any positive integer, and 14 is the weight of CH_2 unit. For example, tetracontane ($C_{40}H_{82}$) is one of the components in oligomers. In GS-ESI inletting process, it is oxidized into $C_{40}H_{79}O^+$, and the m/z of the generated ion is 14*40+13, which belongs to 14x+13 peak series.

Supplementary Table 18. Summary of the product distribution of Reactions 1-4 over LSP-Z100.

Reaction	Components of gasoline range products (%)				
	n-alkanes	i-alkanes	alkene	cycloalkanes	aromatics
R1	11.7	72.5	1.4	4.3	10.1
R2	11.6	70.5	4.8	3.6	9.4
R3	10.6	59.0	14.8	5.1	10.4
R4	10.9	63.4	13.8	3.3	8.6

Supplementary Table 19. Elemental analysis of HDPE powder, mixture of HDPE and LSP-Z100 and reaction solid residue in reaction 1-4 over LSP-Z100 zeolite at 240 ℃ for 4 hours. It shows that the carbon and hydrogen are balanced in the closed system during all reactions.

	HDPE mixed with LSP-Z100	R_1	R_2	R_3	R_4
C in residue (mol)		0.006	0.007	0.009	0.009
H in residue (mol)		0.011	0.013	0.016	0.018
H/C ratio of residue		1.798	1.808	1.876	1.920
C in GC-analyzed product (mol)		0.025	0.024	0.022	0.022
H in GC-analyzed product (mol)		0.055	0.052	0.048	0.047
H/C ratio of GC-analyzed product		2.205	2.200	2.146	2.179
All C in count (mol)	0.031	0.031	0.031	0.031	0.031
All H in count (mol)	0.065	0.065	0.065	0.064	0.065
Overall H/C ratio	2.031	2.075	2.065	2.042	2.073

Supplementary Table 20. Quantification of coke after every reaction by TGA analysis.

	R_1	R_2	R_3	R_4
Coke (mg)	2.615	3.198	3.210	3.510
Yield of coke (*wt.*% of feeding HDPE)	0.581	0.415	0.309	0.280

Supplementary Table 21. Peak assignment of solid state ^{31}P NMR spectra of trimethylphosphine oxide (TMPO) adsorbed on zeolites.

^{31}P chemical shift (ppm)	Corresponding adsorbed species
64 (65)	TMPO adsorbed on work-associated Al Lewis acid sites 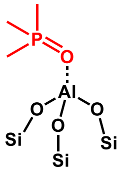
62 (63)	TMPO adsorbed on work-associated Al Lewis acid sites 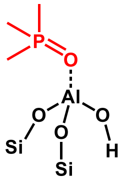
68 (69)	TMPO adsorbed on work-associated Al Lewis acid sites 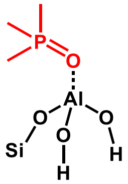
74 (76)	$TMPOH^{+}$ ion formed on Brönsted acid site 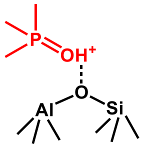
83	$TMPOH^{+}$ ion transfers from the Brönsted acid site to the zeolite channel intersection
89	$TMPOH^{+}$ ion formed on Brönsted/Lewis acid pairs 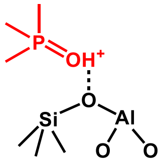

Supplementary Table 22. Mass of ions of 2-methylbutane from GC-MS.

m/z	ion	Generation
73	$C_5H_{11}D^{+\cdot}$	ionization of $C_5H_{11}D$
72	$C_5H_{12}^{+\cdot}$, $C_5H_{10}D^{+}$	ionization of $C_5H_{11}D$, $H\cdot$ loss of $C_5H_{11}D^{+\cdot}$
71	$C_5H_{11}^{+}$	$H\cdot$ loss of $C_5H_{12}^{+\cdot}$, $D\cdot$ loss of $C_5H_{11}D^{+\cdot}$
58	$C_4H_8D^{+}$	Cleavage of $C_5H_{11}D^{+\cdot}$
57	$C_4H_9^{+}$	Cleavage of $C_5H_{12}^{+\cdot}$, Cleavage of $C_5H_{11}D^{+\cdot}$
56	$C_4H_6D^{+}$	H_2 loss of $C_4H_8D^{+}$
55	$C_4H_7^{+}$	H_2 loss of $C_4H_9^{+}$, HD loss of $C_4H_6D^{+}$
44	$C_3H_6D^{+}$	Cleavage of $C_5H_{11}D^{+\cdot}$
43	$C_3H_7^{+}$	Cleavage of $C_5H_{12}^{+\cdot}$, Cleavage of $C_5H_{11}D^{+\cdot}$
42	$C_3H_5D^{+}$	H_2 loss of $C_3H_6D^{+}$
41	$C_3H_5^{+}$	H_2 loss of $C_3H_7^{+}$, HD loss of $C_3H_6D^{+}$
84	$C_5D_{12}^{+\cdot}$	ionization of C_5D_{12}
83	$C_5D_{11}H^{+\cdot}$	ionization of $C_5D_{11}H$
82	$C_5D_{10}H_2^{+\cdot}$, $C_5D_{11}^{+}$	ionization of $C_5D_{10}H_2$, $D\cdot$ loss of $C_5D_{12}^{+\cdot}$, $H\cdot$ loss of $C_5D_{11}H^{+\cdot}$
81	$C_5D_9H_3^{+\cdot}$, $C_5D_{10}H^{+}$	ionization of $C_5D_{10}H_2$, $D\cdot$ loss of $C_5D_{11}H^{+\cdot}$
80	$C_5D_9H_2^{+}$	$D\cdot$ loss of $C_5D_{10}H_2^{+\cdot}$, $H\cdot$ loss of $C_5D_9H_3^{+\cdot}$
66	$C_4D_9^{+}$	Cleavage of $C_5D_{12}^{+\cdot}$, Cleavage of $C_5D_{11}H^{+\cdot}$, Cleavage of $C_5D_{10}H_2^{+\cdot}$, Cleavage of $C_5D_9H_3^{+\cdot}$
65	$C_4D_8H^{+}$	Cleavage of $C_5D_{11}H^{+\cdot}$, Cleavage of $C_5D_{10}H_2^{+\cdot}$, Cleavage of $C_5D_9H_3^{+\cdot}$
64	$C_4D_7H_2^{+}$	Cleavage of $C_5D_{10}H_2^{+\cdot}$, Cleavage of $C_5D_9H_3^{+\cdot}$
63	$C_4D_6H_3^{+}$	Cleavage of $C_5D_9H_3^{+\cdot}$
62	$C_4D_7^{+}$	D_2 loss of $C_4D_9^{+}$, HD loss of $C_4D_8H^{+}$, H_2 loss of $C_4D_7H_2^{+}$
61	$C_4D_6H^{+}$	D_2 loss of $C_4D_8H^{+}$, HD loss of $C_4D_7H_2^{+}$, H_2 loss of $C_4D_6H_3^{+}$
60	$C_4D_5H_2^{+}$	D_2 loss of $C_4D_7H_2^{+}$, HD loss of $C_4D_6H_3^{+}$
50	$C_3D_7^{+}$	Cleavage of $C_5D_{12}^{+\cdot}$, Cleavage of $C_5D_{11}H^{+\cdot}$, Cleavage of $C_5D_{10}H_2^{+\cdot}$, Cleavage of $C_5D_9H_3^{+\cdot}$
49	$C_3D_6H^{+}$	Cleavage of $C_5D_{11}H^{+\cdot}$, Cleavage of $C_5D_{10}H_2^{+\cdot}$, Cleavage of $C_5D_9H_3^{+\cdot}$
48	$C_3D_5H_2^{+}$	Cleavage of $C_5D_{10}H_2^{+\cdot}$, Cleavage of $C_5D_9H_3^{+\cdot}$
47	$C_3D_4H_3^{+}$	Cleavage of $C_5D_9H_3^{+\cdot}$
46	$C_3D_5^{+}$,	D_2 loss of $C_3D_7^{+}$, HD loss of $C_3D_6H^{+}$, H_2 loss of $C_3D_5H_2^{+}$
45	$C_3D_4H^{+}$	D_2 loss of $C_3D_6H^{+}$, HD loss of $C_3D_5H_2^{+}$, H_2 loss of $C_3D_4H_3^{+}$
44	$C_3D_3H_2^{+}$	D_2 loss of $C_3D_5H_2^{+}$, HD loss of $C_3D_4H_3^{+}$

Supplementary Table 23. Mass of ions of hexane from GC-MS.

m/z	ion	Generation
100	$C_6D_{14}^{+\cdot}$	ionization of C_6D_{14}
99	$C_6D_{13}H^{+\cdot}$	ionization of $C_6D_{13}H$
98	$C_6D_{12}H_2^{+\cdot}$, $C_6D_{13}^{+}$	ionization of $C_6D_{12}H_2$, D· loss of $C_6D_{14}^{+\cdot}$, H· loss of $C_6D_{13}H^{+\cdot}$
97	$C_6D_{11}H_3^{+\cdot}$, $C_6D_{12}H^{+}$	ionization of $C_6D_{11}H_3$, D· loss of $C_6D_{13}H^{+\cdot}$, H· loss of $C_6D_{12}H_2^{+\cdot}$
96	$C_6D_{11}H_2^{+}$	D· loss of $C_6D_{12}H_2^{+\cdot\cdot}$, H· loss of $C_6D_{11}H_3^{+\cdot}$
95	$C_6D_{10}H_3^{+}$	D· loss of $C_6D_{11}H_3^{+}$
82	$C_5D_{11}^{+}$	Cleavage of $C_6D_{14}^{+\cdot}$, Cleavage of $C_6D_{13}H^{+\cdot}$, Cleavage of $C_6D_{12}H_2^{+\cdot}$, Cleavage of $C_6D_{11}H_3^{+\cdot}$
81	$C_5D_{10}H^{+}$	Cleavage of $C_6D_{13}H^{+\cdot}$, Cleavage of $C_6D_{12}H_2^{+\cdot}$, Cleavage of $C_6D_{11}H_3^{+\cdot}$
80	$C_5D_9H_2^{+}$	Cleavage of $C_6D_{12}H_2^{+\cdot}$, Cleavage of $C_6D_{11}H_3^{+\cdot}$
79	$C_5D_8H_3^{+}$	Cleavage of $C_6D_{11}H_3^{+\cdot}$
78	$C_5D_9^{+}$	D_2 loss of $C_5D_{11}^{+}$, HD loss of $C_5D_{10}H^{+}$, H_2 loss of $C_5D_9H_2^{+}$
77	$C_5D_8H^{+}$	D_2 loss of $C_5D_{10}H^{+}$, HD loss of $C_5D_9H_2^{+}$, H_2 loss of $C_5D_8H_3^{+}$
76	$C_5D_7H_2^{+}$	D_2 loss of $C_5D_9H_2^{+}$, HD loss of $C_5D_8H_3^{+}$
66	$C_4D_9^{+}$	Cleavage of $C_6D_{14}^{+\cdot}$, Cleavage of $C_6D_{13}H^{+\cdot}$, Cleavage of $C_6D_{12}H_2^{+\cdot}$, Cleavage of $C_6D_{11}H_3^{+\cdot}$
65	$C_4D_8H^{+}$	Cleavage of $C_6D_{13}H^{+\cdot}$, Cleavage of $C_6D_{12}H_2^{+\cdot}$, Cleavage of $C_6D_{11}H_3^{+\cdot}$
64	$C_4D_7H_2^{+}$	Cleavage of $C_6D_{12}H_2^{+\cdot}$, Cleavage of $C_6D_{11}H_3^{+\cdot}$
63	$C_4D_6H_3^{+}$	Cleavage of $C_6D_{11}H_3^{+\cdot}$
62	$C_4D_7^{+}$	D_2 loss of $C_4D_9^{+}$, HD loss of $C_4D_8H^{+}$, H_2 loss of $C_4D_7H_2^{+}$
61	$C_4D_6H^{+}$	D_2 loss of $C_4D_8H^{+}$, HD loss of $C_4D_7H_2^{+}$, H_2 loss of $C_4D_6H_3^{+}$
60	$C_4D_5H_2^{+}$	D_2 loss of $C_4D_7H_2^{+}$, HD loss of $C_4D_6H_3^{+}$
50	$C_3D_7^{+}$	Cleavage of $C_6D_{14}^{+\cdot}$, Cleavage of $C_6D_{13}H^{+\cdot}$, Cleavage of $C_6D_{12}H_2^{+\cdot}$, Cleavage of $C_6D_{11}H_3^{+\cdot}$
49	$C_3D_6H^{+}$	Cleavage of $C_6D_{13}H^{+\cdot}$, Cleavage of $C_6D_{12}H_2^{+\cdot}$, Cleavage of $C_6D_{11}H_3^{+\cdot}$
48	$C_3D_5H_2^{+}$	Cleavage of $C_6D_{12}H_2^{+\cdot}$, Cleavage of $C_6D_{11}H_3^{+\cdot}$
47	$C_3D_4H_3^{+}$	Cleavage of $C_6D_{11}H_3^{+\cdot}$
46	$C_3D_5^{+}$,	D_2 loss of $C_3D_7^{+}$, HD loss of $C_3D_6H^{+}$, H_2 loss of $C_3D_5H_2^{+}$
45	$C_3D_4H^{+}$	D_2 loss of $C_3D_6H^{+}$, HD loss of $C_3D_5H_2^{+}$, H_2 loss of $C_3D_4H_3^{+}$
44	$C_3D_3H_2^{+}$	D_2 loss of $C_3D_5H_2^{+}$, HD loss of $C_3D_4H_3^{+}$

Supplementary Table 24. Summary of acidities before and after dealumination of LSP-Z100. Data were determined by NH_3-TPD and Pyridine-IR studies.

Catalyst	Total acid[a] ($mmol \cdot g^{-1}$)	L/B acid ratio[b]	L acid ($mmol \cdot g^{-1}$)	B acid ($mmol \cdot g^{-1}$)
LSP-Z100	0.191	1.049	0.098	0.093
De-Al LSP-Z100	0.096	0.195	0.016	0.080

[a]From NH_3-TPD

[b]From Pyridine-IR

Supplementary Table 25. Peak assignment of the INS spectra of solid HDPE, reacted HDPE over LSP-Z100 and possible products adsorbed on LSP-Z100. The assignment is guided by calculation and Ref.[17,18,34,35]

INS Spectrum	Wavenumber (cm^{-1})	Vibrational mode
HDPE	97	Out-of-plane skeletal mode
	130	In-plane skeletal mode
	200	Out-of-plane skeletal mode
	525	In-plane skeletal mode
	725	$-CH_2-$ rock
	1065	C-C stretch
	1187	$-CH_2-$ rock
	1303	Multiple $-CH_2-$ twist/wag modes along molecular axis
	1377	$-CH_2-$ wag
	1449	$-CH_2-$ in-plane HCH deformation
Adsorbed oligomers	235	-CH3 torsion of oligomer
	284	Longitudinal acoustic C-C stretch
	726	$-CH_2-$ rock
	1052	$-CH_2-$ twist
	1304	Multiple $-CH_2-$ twist/wag modes along molecular axis
	1371	$-CH_2-$ wag<br>-CH3 symmetric deformation
	1456	$-CH_2-$ in-plane HCH deformation
	227, 255	-CH3 torsion
	324, 377, 443	C-C-C deformation
	739	$-CH_2-$ rock
	816, 858	Longitudinal acoustic vibrational of skeletal

2-methylpentane	892, 915.958	-CH_3 terminal rock
	1014	-CH_2- twist
	1068	-CH_2- wag<br><br>Longitudinal acoustic vibrational of skeletal
	1157	-CH_2- rock
	1181	-CH_3 terminal rock
	1247, 1280, 1302	Multiple -CH_2- twist/wag modes along molecular axis
	1371	tertiary C-H deformation<br><br>-CH_2- wag
	1456	-CH_2- in-plane HCH deformation
3-methylpentane	212, 266	-CH_3 torsion
	319, 386, 440	C-C-C deformation
	764	-CH_2- rock
	814, 879	Longitudinal acoustic vibrational of skeletal
	952, 980	-CH_3 terminal rock
	1012	-CH_2- twist
	1043	-CH_2- wag<br><br>Longitudinal acoustic vibrational of skeletal
	1160	-CH_2- rock
	1280	Multiple -CH_2- twist/wag modes along molecular axis
	1382	tertiary C-H deformation<br><br>-CH_2- wag
	1458	-CH_2- in-plane HCH deformation

Supplementary Table 26. Predicted total capital investment.

	Purchase cost[36]	Installation cost[36]	Number of equipment[d]		
			Route A (This work)	Route .B (Ref.[28])	Route C (Ref.[3])
Plastic pretreatment line[a] / $	450,000	450,000	8	8*46	8*0.5
Transfer conveyor / $	3,250,000	5,520,000	4	4*46	4*0.5
Tank agitator / $	220,000	439,000	8	8*46	8*0.5
Heat exchanger / $	97,500	214,500	16	16*46	16*0.5
Centrifuge / $	6,380,000	6,380,000	4	4*46	4*0.5
Storage / $	199,000	339,000	8	8*46	8*0.5
Rectification Column System / $	3,350,232	8,040,557	-	-	1
Catalyst recovery furnace / $	30,500	45,700	8	8*46	8*0.5
			**Cost**		
			Route A (This work)	Route B	Route C
**Totals**			**61,211,600**	**2,815,734,000**	**38,646,357**
warehouse / $			2,304,464	106,005,360	1,473,854
Site development / $			5,185,044	238,512,060	3,316,172
Additional piping / $			2,592,522	119,256,030	1,658,086
**Total Direct Costs (TDC) / $**			**71,293,630**	**3,279,507,450**	**45,094,469**
Prorateable expenses / $		10.0% of TDC	7,129,363	327,950,745	4,509,447
Field expenses / $		10.0% of TDC	7,129,363	327,950,745	4,509,447
Home office & Construction fee / $		20.0% of TDC	14,258,726	655,901,490	9,018,894
Project contingency / $		10.0% of TDC	7,129,363	327,950,745	4,509,447

Other costs / $	10.0% of TDC	7,129,363	327,950,745	4,509,447
**Total indirect costs (TIC) / $**		**42,776,178**	**1,967,704,470**	**27,056,682**
**Fixed Capital Investment (FCI)[b] / $**		**114,069,808**	**5,247,211,920**	**72,151,151**
Land / $		1,848,000	85,008,000	924,000
Working capital / $	5.0% of FCI	806,562	37,101,876	3,607,558
**Total capital investment (TCI)[c] / $**		**121,621,298**	**5,594,580,516**	**76,682,709**

[a]Estimated from prices in https://xtingmachine.en.made-in-china.com for a 3000 kg/h PE recycling machine.

[b]FCI = TDC + TIC

[c]TCI = FCI + Land + Working capital

[d]The number of equipment is estimated with concern of reaction time, yield and command of product separation in different work to achieve the same production yield.

Supplementary Table 27. Predicted total variable operational costs.

**Route A (This work)**	Usage (unit/h)	Cost ($/unit)	$/hour	$/MT product
Waste PE	10 MT	130 / MT[a]	1,300	162.5
Price range of waste PE		100-300 /MT	1,000-3,000	125-375
**Feedstock**			**1,300**	**162.5**
**Industrial electricity**	24,000 kW·h	0.09 / kWh[b]	**2,160**	**270**
Catalyst	0.03 MT	5,000 / MT[d]	150	18.75
**Other cost**			**150**	**18.75**
**Total variable operating costs**			**3,610**	**451.25**
Range of Total variable operating costs			3,310 to 5,310	413.75 to 663.75
**Route B**	Usage (unit/h)	Cost ($/unit)	$/hour	$/MT product
Waste PE	14 MT	130 /MT[a]	1,820	227.5
Price range of waste PE		100-300 /MT	1,400-4,200	175-525
Hydrogen for production	35 kg	1.7/kg[e]	59.5	7.44
Hydrogen for catalyst recovery	42 kg	1.7/kg[e]	71.4	8.92
**Feedstock**			**1950.9**	**243.9**
**Industrial electricity**	1,104,000 kW·h	0.09 / kWh[b]	**99,360**	**12,420**
Catalyst Pt	0.024 kg	29,738 / kg[f]	416.4	52.05
Functionalized SiO_2 support	3.3 kg	10 / kg[d]	33	4.12
**Other cost**			**449.4**	**56.17**
**Total variable operating costs**			**101,760.30**	**12,720.07**
Range of Total variable operating costs			101,340.30 to 104,140.30	12,667.54 to 13,017.54
**Route C**	Usage (unit/h)	Cost ($/unit)	$/hour	$/MT product
Waste PE	11 MT	130 / MT[a]	1,430	178.8
Price range of waste PE		100-300 /MT	1,100-3,300	137.5-412.5

Hydrogen for production	19.8 kg	1.7 / kg[d]	33.7	4.2
Hydrogen for catalyst recovery	66 kg	1.7 / kg[d]	112.2	14.0
**Feedstock**			**3,445.9**	**430.7**
**Industrial electricity**	13,000 kW·h	0.09 / kWh[b]	**1,170**	**146.2**
Catalyst Pt	0.07 kg	29,738 / kg[e]	2081.6	260.2
WO_3	2.1 kg	10 / kg[d]	21	2.6
ZrO_2	11.8 kg	10 / kg[d]	118	14.75
Zeolite HY	25 kg	5 / kg[d]	125	17
**Other cost**			**2,345.6**	**293.2**
**Total variable operating costs**			**6961.5**	**870.2**
Range of Total variable operating costs			6191.5 to 8391.5	773.9 to 1048.9

[a] Price of collecting waste PE estimated by Ref.[37] The price varies in a range of 100-300 $/MT because of different PE concentration in plastic wastes.

[b] Estimated from price in https://www.statista.com/ (accessed in June 2022)

[c] Estimated from price in https://www.alibaba.com (accessed in June 2022)

[d] Calculation method of total variable operating costs:

1. The amount of HDPE to be converted and electricity per hour are estimated with concern of reaction temperature, time and yield in different work to achieve the same production yield.
2. The usage of zeolite catalyst in Route A (this work), Route C are calculated with regard of 20 times regeneration cycle, which is below the regeneration times of zeolite catalysts in petrochemical industry[38].
3. The usage of catalyst in Routes B are estimated with regard of 5% catalyst activity loss because of Pt leasing, sintering, redispersion and fouling. The average usage of catalyst= (initial usage + refill usage* refill times)/(1+refill times). After about 4 years of regeneration, the catalyst can't be reused anymore[39].
4. The usage of hydrogen is estimated by the actual need after recovery of remaining fraction.

[e] From data in https://www.iea.org/ (accessed in June 2022)

[f] From data in https://www.bullionvault.com/ (accessed in June 2022)

Supplementary Table 28. Summary economic analysis.

	Route A (This work)	Route B	Route C
Plant life / years	40	40	40
Production / MT day^{-1}	192	192	192 for gasoline<br>4.7 for diesel
Product price / \$ MT^{-1}	1538[a]	990[b]	1538[a] for gasoline<br>1455[a] for diesel
Total capital investment / \$	121,621,298	5,594,580,516	76,682,709
Total variable operating cost / \$ $year^{-1}$	31,623,600	891,420,228	60,982,740
Total cost / \$ $(MT\ product)^{-1}$	494.6	14,716	897.5
**Profit / \$ $(MT\ product)^{-1}$**	**1043.4**	**-13,726**	**638.5**
Error range of profit / \$ $(MT\ product)^{-1}$	830.9 to 1080.9	-13,673 to -14,023	459.7 to 734.7
**Profit / \$ $year^{-1}$**	**73,191,552**	**No revenue**	**44,746,080**
Range of profit / \$ $year^{-1}$	58,226,908 to 75,746,908	No revenue	32,216,272 to 51,488,272

[a]Estimated from data in U.S. energy information administration website.

[b]Estimated from data in https://tradingeconomics.com/ (accessed in June 2022)

Supplementary Table 29. CO_2 emission of liquid fuel production using strategy of this work, PE pyrolysis, and crude oil-based refinery.

**This work**	Carbon emission (kg $_{CO2}$·MT $_{PE}^{-1}$)	Carbon emission (kg $_{CO2}$·MT $_{gasoline}^{-1}$)
PE collection and cleaning	1.7	1.8
**Raw material collection**	**1.7**	**1.8**
Catalytic cracking	301.2	333.5
Catalyst preperation	56.6	62.7
Reaction residue incineration and catalyst regeneration	208.7	231.1
**Subsequent processing**	**566.5**	**627.3**
**Entire production process**	**568.2**	**629.1**
**PE pyrolysis**	Carbon emission (kg $_{CO2}$·MT $_{PE}^{-1}$)	Carbon emission (kg $_{CO2}$·MT $_{liquid\ fuel}^{1}$)
PE collection and cleaning	1.7	1.8
**Raw material collection**	**1.7**	**1.8**
Pyrolysis	150.0	221.9
Pre-hydrogenation	210.0	310.7
Catalytic cracking	220.0	325.4
Hydrogenation of gasoline	10.0	14.8
Hydrogenation of diesel	24.0	35.5
**Subsequent processing**	**614.0**	**908.3**
**Entire production process**	**615.7**	**910.1**
**Crude oil refinery**	Carbon emission (kg $_{CO2}$·MT $_{crude\ oil}^{-1}$)	Carbon emission (kg $_{CO2}$·MT $_{liquid\ fuel}^{1}$)
Crude oil extraction	332.0	488.2
**Raw material collection**	**332.0**	**488.2**

Pre-hydrogenation	44.0	64.7
Atmospheric and vacuum distillation	39.0	57.4
Hydrogenation of slurry oil	63.0	92.6
Hydrogenation of jet fuel	2.0	2.9
Hydrogenation of diesel	40.0	58.8
hydrocracking	56.0	82.3
Catalytic cracking	74.0	108.8
Hydrogenation of gasoline	5.0	7.4
**Subsequent processing**	**323.0**	**475.0**
**Entire production process**	**655.0**	**963.2**

Supplementary references:

1. Duan, J. *et al.* Coking-resistant polyethylene upcycling modulated by zeolite micropore diffusion. *J. Am. Chem. Soc.* **144**, 14269–14277 (2022).
2. Zhang, W. *et al.* Low-temperature upcycling of polyolefins into liquid alkanes via tandem cracking-alkylation. *Science* **379**, 807–811 (2023).
3. Liu, S., Kots, P. A., Vance, B. C., Danielson, A. & Vlachos, D. G. Plastic waste to fuels by hydrocracking at mild conditions. *Sci. Adv.* **7**, eabf8283 (2021).
4. Michael Gytarsky *et al. 2006 IPCC guidelines for National Greenhouse Gas Inventories.* https://www.ipcc.ch/report/2006-ipcc-guidelines-for-national-greenhouse-gas-inventories/.
5. GB/T 2589-2020 General rules for calculation of the comprehensive energy consumption. https://openstd.samr.gov.cn.
6. Parry, E. P. An infrared study of pyridine adsorbed on acidic solids. Characterization of surface acidity. *J. Catal.* **2**, 371–379 (1963).
7. Corma, A. *et al.* 2,6-Di-tert-butyl-pyridine as a probe molecule to measure external acidity of zeolites. *J. Catal.* **179**, 451–458 (1998).
8. Kots, P. A. *et al.* Polypropylene plastic waste conversion to lubricants over Ru/TiO_2 Catalysts. *ACS Catal.* **11**, 8104–8115 (2021).
9. Rorrer, J. E., Troyano-Valls, C., Beckham, G. T. & Román-Leshkov, Y. Hydrogenolysis of polypropylene and mixed polyolefin plastic waste over Ru/C to produce liquid alkanes. *ACS Sustain. Chem. Eng.* **9**, 11661–11666 (2021).
10. Chen, L. *et al.* Disordered, sub-nanometer Ru structures on CeO_2 are highly efficient and selective catalysts in polymer upcycling by hydrogenolysis. *ACS Catal.* **12**, 4618–4627 (2022).
11. Weitkamp, J., Jacobs, P. A. & Martens, J. A. Isomerization and hydrocracking of C_9 through C_{16} n-alkanes on Pt/HZSM-5 zeolite. *Appl. Catal.* **8**, 123–141 (1983).
12. Lin, L. *et al.* Acid strength controlled reaction pathways for the catalytic cracking of 1-pentene to propene over ZSM-5. *ACS Catal.* **5**, 4048–4059 (2015).
13. Li, Q., Tang, Y. & Xiang, J. An on-line NMR chemical shift prediction platform based on density functional theory. *Chinese J. Magn. Reson.* **38**, 22–31 (2021).
14. Chen, S. & Manos, G. Study of coke and coke precursors during catalytic cracking of n-hexane and 1-hexene over ultrastable Y zeolite. *Catal. Lett.* **96**, 195–200 (2004).
15. Corma, A. & Orchillés, A. V. Current views on the mechanism of catalytic cracking. *Microporous Mesoporous Mater.* **35–36**, 21–30 (2000).
16. Dapsens, P. Y., Mondelli, C. & Pérez-Ramírez, J. Design of Lewis-acid centres in zeolitic matrices for the conversion of renewables. *Chem. Soc. Rev.* **44**, 7025–7043 (2015).
17. Hawkins, A. P. *et al.* Onset of propene oligomerization reactivity in ZSM-5 studied by inelastic neutron scattering spectroscopy. *ACS Omega* **5**, 7762–7770 (2020).
18. Hawkins, A. P. *et al.* Investigation of the dynamics of 1-octene adsorption at 293 K in a ZSM-5 catalyst by inelastic and quasielastic neutron scattering. *J. Phys. Chem. C* **123**, 417–425 (2019).
19. Masnadi, M. S. & Brandt, A. R. Climate impacts of oil extraction increase significantly with oilfield age. *Nat. Clim. Change* **7**, 551–556 (2017).
20. Ragusa, A. *et al.* Plasticenta: First evidence of microplastics in human placenta. *Environ. Int.* **146**, 106274 (2021).
21. Rochman, C. M. & Hoellein, T. The global odyssey of plastic pollution. *Science* **368**, 1184–1185 (2020).
22. Christian B., Lynne B. M., Darren B. & Bernd M. *Database of Zeolite Structures*. https://www.iza-structure.org/databases/

23. Conk, R. J. *et al.* Catalytic deconstruction of waste polyethylene with ethylene to form propylene. *Science* **377**, 1561–1566 (2022).
24. Dong, Q. *et al.* Depolymerization of plastics by means of electrified spatiotemporal heating. *Nature* **616**, 488–494 (2023).
25. Sullivan, K. P. *et al.* Mixed plastics waste valorization through tandem chemical oxidation and biological funneling. *Science* **378**, 207–211 (2022).
26. Rorrer, J. E., Beckham, G. T. & Román-Leshkov, Y. Conversion of polyolefin waste to liquid alkanes with Ru-based catalysts under mild conditions. *JACS Au* **1**, 8–12 (2021).
27. Wang, C. *et al.* Polyethylene hydrogenolysis at mild conditions over ruthenium on tungstated zirconia. *JACS Au* **1**, 1422–1434 (2021).
28. Tennakoon, A. *et al.* Catalytic upcycling of high-density polyethylene via a processive mechanism. *Nat. Catal.* **3**, 893–901 (2020).
29. Jia, X., Qin, C., Friedberger, T., Guan, Z. & Huang, Z. Efficient and selective degradation of polyethylenes into liquid fuels and waxes under mild conditions. *Sci. Adv.* **2**, e1501591 (2016).
30. Zhang, F. *et al.* Polyethylene upcycling to long-chain alkylaromatics by tandem hydrogenolysis/aromatization. *Science* **370**, 437–441 (2020).
31. Zhang, Z. *et al.* Recovering waste plastics using shape-selective nano-scale reactors as catalysts. *Nat. Sustain.* **2**, 39–42 (2019).
32. Vollmer, I., Jenks, M. J. F., Mayorga González, R., Meirer, F. & Weckhuysen, B. M. Plastic waste conversion over a refinery waste catalyst. *Angew. Chem. Int. Ed.* **60**, 16101–16108 (2021).
33. Protić-Lovasić, G., Jambrec, N., Deur-Siftar, D. & Prostenik, M. V. Determination of catalytic reformed gasoline octane number by high resolution gas chromatography. *Fuel* **69**, 525–528 (1990).
34. Parker, S. F. Inelastic neutron scattering spectra of polyethylene. *J. Chem. Soc., Faraday Trans.* **92**, 1941 (1996).
35. Frisch, M. J. *et al.* Gaussian 16 Revision C.01. (2016).
36. Humbird, D. *et al. Process Design and Economics for Biochemical Conversion of Lignocellulosic Biomass to Ethanol: Dilute-Acid Pretreatment and Enzymatic Hydrolysis of Corn Stover*. NREL/TP-5100-47764, 1013269 http://www.osti.gov/servlets/purl/1013269/ (2011) doi:10.2172/1013269.
37. Hernández, B., Kots, P., Selvam, E., Vlachos, D. G. & Ierapetritou, M. G. Techno-economic and life cycle analyses of thermochemical upcycling technologies of low-density polyethylene waste. *ACS Sustain. Chem. Eng.* **11**, 7170–7181 (2023).
38. Chiranjeevi, T., Pragya, R., Gupta, S., Gokak, D. T. & Bhargava, S. Minimization of waste spent catalyst in refineries. *Procedia Environ. Sci.* **35**, 610–617 (2016).
39. Padamata, S. K., Yasinskiy, A. S., Polyakov, P. V., Pavlov, E. A. & Varyukhin, D. Yu. Recovery of noble metals from spent catalysts: A review. *Metall. Mater. Trans. B* **51**, 2413–2435 (2020).
